# Composition and Associations of the Infant Gut Fungal Microbiota with Environmental Factors and Childhood Allergic Outcomes

**DOI:** 10.1128/mBio.03396-20

**Published:** 2021-06-01

**Authors:** Rozlyn C. T. Boutin, Hind Sbihi, Ryan J. McLaughlin, Aria S. Hahn, Kishori M. Konwar, Rachelle S. Loo, Darlene Dai, Charisse Petersen, Fiona S. L. Brinkman, Geoffrey L. Winsor, Malcolm R. Sears, Theo J. Moraes, Allan B. Becker, Meghan B. Azad, Piush J. Mandhane, Padmaja Subbarao, Stuart E. Turvey, B. Brett Finlay

**Affiliations:** a University of British Columbia, Vancouver, Canada; b Michael Smith Laboratories, Vancouver, Canada; c Government of British Columbia Ministry of Health, Office of the Public Health Officer, Vancouver, Canada; d Koonkie Inc., Menlo Park, California, USA; e British Columbia Children’s Hospital, Vancouver, Canada; f Simon Fraser University, Vancouver, Canada; g McMaster University, Hamilton, Canada; h Hospital for Sick Children, Toronto, Canada; i University of Manitoba, Winnipeg, Canada; j University of Alberta, Edmonton, Canada; Temple University; Cornell University

**Keywords:** asthma/allergy, atopy, fungi, infant immune development, microbiota, mycobiota

## Abstract

Although often neglected in gut microbiota studies, recent evidence suggests that imbalanced, or dysbiotic, gut mycobiota (fungal microbiota) communities in infancy coassociate with states of bacterial dysbiosis linked to inflammatory diseases such as asthma. In the present study, we (i) characterized the infant gut mycobiota at 3 months and 1 year of age in 343 infants from the CHILD Cohort Study, (ii) defined associations among gut mycobiota community composition and environmental factors for the development of inhalant allergic sensitization (atopy) at age 5 years, and (iii) built a predictive model for inhalant atopy status at age 5 years using these data. We show that in Canadian infants, fungal communities shift dramatically in composition over the first year of life. Early-life environmental factors known to affect gut bacterial communities were also associated with differences in gut fungal community alpha diversity, beta diversity, and/or the relative abundance of specific fungal taxa. Moreover, these metrics differed among healthy infants and those who developed inhalant allergic sensitization (atopy) by age 5 years. Using a rationally selected set of early-life environmental factors in combination with fungal community composition at 1 year of age, we developed a machine learning logistic regression model that predicted inhalant atopy status at 5 years of age with 81% accuracy. Together, these data suggest an important role for the infant gut mycobiota in early-life immune development and indicate that early-life behavioral or therapeutic interventions have the potential to modify infant gut fungal communities, with implications for an infant’s long-term health.

## INTRODUCTION

Early infancy represents a critical window of life during which cross talk between an infant’s developing immune system and maturing gut microbiota has important long-term and wide-ranging consequences for host immune health (1–4). The dynamics of bacterial community organization in the infant gut, environmental or developmental factors contributing to this process, and the consequences of disruptions to “normal” gut bacterial populations for infant health have been well studied (2–5). It is known, for instance, that both immune disease risk and bacterial community composition are influenced by early-life environmental factors such as birth mode (6, 7) and place of delivery (8), diet (9), birth order (10, 11), pet (12, 13) and/or farm (14) exposure, antibiotic use (15, 16), and daycare attendance (17, 18). Weaning and the transition to solid food from breast milk or formula is one of the final major milestones causing dramatic shifts in infant gut bacterial populations toward more adult-like states, with implications for later immunity and health outcomes (19, 20). In contrast, despite knowledge of their existence in the healthy infant gut (5), little is known about potential environmental sources or modifiers of members of the “rare biosphere” within the gut microbiota, including *Archaea*, fungi, and other microeukaryotes, or how these organisms interact with the host and bacteria to ultimately shape immune development and gut microbiota communities, respectively.

Despite several unique challenges associated with the sequencing and analysis of fungal microbiotas (21), studies have recently begun to characterize the fungal communities in the human infant gut. *Candida*, *Rhodotorula*, *Malassezia*, *Saccharomyces*, and *Debaryomyces* are among the most abundant fungal genera commonly identified in infant gut mycobiota studies (22–27). However, the infant gut mycobiota exhibits a high degree of variability both within and between individuals (22, 26), highlighting a need for additional studies with more sample collection time points to fully characterize temporal trends in infant gut fungal community composition (24–30).

A lack of colonization resistance within the infant gut may render the infant intestines susceptible to sporadic fungal colonization derived from early-life environmental exposures, including diet and birth mode. Breast milk and neonatal intensive care unit surfaces have recently been described as potential sources (31) or modifiers of the presence (30, 32, 33) of fungi within the infant gut mycobiota. Fungi have also been detected in breast milk of mothers in multiple studies (31, 34, 35), including the CHILD Cohort Study (36). Diet has also been described as the primary determinant of fungal community composition within the gut microbiota of adult humans (37, 38). Diet and breastfeeding practices therefore have the potential to influence infant gut mycobiota communities, but this has yet to be specifically investigated. Furthermore, it is possible that vertical transmission of vaginal *Candida* (39, 40) or skin-associated *Malassezia* (41, 42) occurs during or after birth and that birth mode may affect the composition of the infant gut mycobiota (22, 26, 29).

Characterizing the effects of environmental exposures on infant gut microbiota populations has important implications for guiding human behavior, as early-life factors associated with reduced microbial exposures (hygiene and urbanization), impaired gut microbiota maturation, and dysbiotic infant gut microbiota communities have been implicated in a wide range of autoimmune and allergic disorders later in life (43). Asthma and atopy are among the best-characterized diseases that have been shown in multiple birth cohort and animal studies to be associated with a dysbiotic community of gut microbes (bacteria) within the first 100 days of life (24, 25, 44–50). Two studies have recently shown that fungal communities in the infant gut differ dramatically according to asthma and atopy risk and in association with bacterial dysbiosis (24, 25). Notably, severe and remission-resistant asthma is often associated with sensitization to inhaled allergens containing fungi, including house dust mites (HDM) (51, 52) and *Alternaria* (53). Moreover, single nucleotide polymorphisms in the genes encoding the fungal recognition receptors dectin-1 (*CLEC7A*) and mannose-binding lectin (*MBL2*) have been associated with allergic asthma susceptibility (54). Thus, fungi may be a crucial but overlooked piece of the puzzle linking dysbiosis in the infant gut to childhood asthma and atopy.

Here, we describe shifts in community composition of the gut mycobiota during the first year of life, identify environmental factors associated with variation in gut fungal communities, and define the role of fungi in the dysbiosis-asthma/atopy paradigm in a subset of children from the well-characterized CHILD Cohort Study (55). Specifically, we profiled the fungal communities present in stool samples collected at visits scheduled for 3 months and 1 year of age from a subset of 343 children by amplifying the internal transcribed spacer 2 (ITS-2) region of fungal DNA isolated from these samples. These data were integrated with 16S rRNA gene amplicon sequencing data from the same samples and comprehensive prospectively collected data on environmental exposures to identify factors that may shape the fungal community composition. Fungal sequencing data were also combined with health outcomes to identify features and microbial signatures of fungal dysbiosis associated with an infants’ risk of developing IgE-mediated allergic sensitization (or atopy) to inhalant allergens at age 5 years. Finally, we used a machine learning logistic regression model to select key early-life environmental factors in combination with fungal community composition at 1 year of age and predict inhalant atopy status at age 5 years.

## RESULTS

### Cohort characteristics.

This study involved 343 subjects in the CHILD Cohort Study who had previously had 16S rRNA gene sequencing completed on stool DNA (15, 56). Because little is known about the dynamics of fungal communities in the infant gut across the first year of life, what early-life factors influence the composition of the early-life mycobiota, or how bacterial and fungal communities change relative to one another over time, we performed ITS-2 rRNA gene sequencing and quantitative PCR (qPCR)-based total fungal load analyses on DNA isolated from fecal samples collected at both 3 months and 1 year of age. Importantly, this subset of 343 children was representative of the full CHILD cohort (overall cohort) and of the cohort with data available on inhalant atopy status at age 5 years (CHILD Study) (Table 1), except that, as expected based on preselection of samples with the goal of enriching for samples from children with available stool and asthma at age 5 years (15), we had more subjects from Vancouver and more subjects with inhalant atopy and asthma at age 5 years.

**TABLE 1:** Cohort characteristics^*a*^

Variable	Overall CHILD	CHILD study 5y	*P* value^*b*^	ITS-2	*P* value^*c*^
No. of patients	3,264	2,539		308	
Institution, no. (%)			<0.001		<0.001
Edmonton	769 (23.6)	544 (21.4)		32 (10.4)	
Toronto	770 (23.6)	519 (20.4)		77 (25)	
Vancouver	730 (22.4)	601 (23.7)		87 (28.2)	
Winnipeg	995 (30.5)	875 (34.5)		112 (36.4)	
Asthma at yr 5, no. (%)			<0.001		0.0022
Asthma	164 (5)	160 (6.3)		33 (10.7)	
No asthma	2,235 (68.5)	2,138 (84.2)		239 (77.6)	
No phenotype available	620 (19)	5 (0.2)		3 (1)	
Possible asthma	245 (7.5)	236 (9.3)		33 (10.7)	
Inhalant atopy at yr 5, no. (%)		,	<0.001		0.0013
Inhalant atopy (ever)	438 (13.4)	438 (17.3)		77 (25)	
No inhalant atopy	2,101 (64.4)	2,101 (82.7)		231 (75)	
Antibiotic use by age 3 mo, no. (%)			0.89		0.87
	586 (18)	452 (17.8)		53 (17.2)	
Antibiotic use by age 1 yr, no. (%)			0.065		0.75
	520 (15.9)	451 (17.8)		57 (18.5)	
Antifungal use by 3 mo, no. (%)			0.76		1
Confirmed	24 (0.7)	24 (0.9)		24 (7.8)	
Unknown	2,921 (89.5)	2,231 (87.9)			
Antifungal use by age 1 yr, no. (%)			0.77		1
Confirmed	26 (0.8)	26 (1)		26 (8.4)	
Unknown	2,921 (89.5)	2,231 (87.9)			
Gestational age at delivery (wks)			0.67		0.47
Median (range)	39.7 (34, 42.9)	39.7 (34, 42.9)		39.9 (34.4, 42.7)	
IQR (Q1, Q3)	38.9, 40.4	38.9, 40.6		38.9, 40.6	
Unknown, no. (%)	154 (4.7)	127 (5)		14 (4.5)	
Mode of delivery, no. (%)			0.95		0.76
Vaginal	2,408 (73.8)	1,879 (74)		228 (74)	
C-section with labor	425 (13)	324 (12.8)		42 (13.6)	
C-section without labor	390 (11.9)	306 (12.1)		33 (10.7)	
Unknown	41 (1.3)	30 (1.2)		5 (1.6)	
Having older sibling, no. (%)			0.98		0.14
Confirmed	1,548 (47.4)	1,206 (47.5)		132 (42.9)	
Unknown	59 (1.8)	45 (1.8)		7 (2.3)	
Male, no. (%)			0.67		0.28
	1,715 (52.5)	1,350 (53.2)		174 (56.5)	
Birth wt Z score			0.64		0.49
Median (range)	−0.1 (−5.9, 15.9)	−0.1 (−5.9, 15.8)		0 (−2.2, 15.1)	
IQR (Q1, Q3)	−0.7, 0.6	−0.7, 0.6		−0.6, 0.6	
Unknown	154 (4.7)	127 (5)		14 (4.5)	
Parental atopy, no. (%)			0.81		0.31
Confirmed	2,447 (75)	1,997 (78.7)		251 (81.5)	
Unknown	246 (7.5)	68 (2.7)		7 (2.3)	
Duration of exclusive breastfeeding (mo)			0.32		0.56
Median (range)	4 (0, 9)	4 (0, 9)		4.5 (0, 6)	
IQR (Q1, Q3)	0.5, 5	0.5, 5		0.6, 5	
Unknown	209 (6.4)	62 (2.4)		1 (0.3)	
Tobacco smoke exposure to age 1 yr, no. (%)			0.044		0.13
Confirmed	808 (24.8)	610 (24)		64 (20.8)	
Unknown	530 (16.2)	276 (10.9)		25 (8.1)	
Season of birth, no. (%)			0.85		0.62
Spring	889 (27.2)	711 (28)		93 (30.2)	
Summer	829 (25.4)	624 (24.6)		80 (26)	
Fall	754 (23.1)	581 (22.9)		69 (22.4)	
Winter	788 (24.1)	621 (24.5)		66 (21.4)	
Unknown	4 (0.1)	2 (0.1)			
Area type, no. (%)			1		0.54
Rural	83 (2.5)	74 (2.9)		22 (7.1)	
Urban	870 (26.7)	775 (30.5)		272 (88.3)	
Unknown	2,311 (70.8)	1,690 (66.6)		14 (4.5)	
Breastfeeding status at 3 mo, no. (%)			0.61		0.28
None	446 (13.7)	334 (13.2)		31 (10.1)	
Partial	821 (25.2)	657 (25.9)		85 (27.6)	
Exclusive	1,884 (57.7)	1,528 (60.2)		192 (62.3)	
Unknown	113 (3.5)	20 (0.8)			
Breastfeeding status at 12 mo, no. (%)	,		0.23		0.81
Confirmed	1358 (41.6)	1,164 (45.8)		141 (45.8)	
Unknown	311 (9.5)	97 (3.8)		7 (2.3)	
Solid food by age 3 mo, no. (%)			0.22		0.91
Confirmed	243 (7.4)	173 (6.8)		22 (7.1)	
Unknown	261 (8)	126 (5)		9 (2.9)	
Presence of mold in home, no. (%)			0.66		0.24
Confirmed	1,238 (37.9)	979 (38.6)		130 (42.2)	
Unknown	21 (0.6)	13 (0.5)			
Oral thrush by age 1 yr, no. (%)			1		0.57
Confirmed	34 (1)	29 (1.1)		2 (0.6)	
Unknown	675 (20.7)	345 (13.6)		23 (7.5)	
Ethnicity of child, no. (%)			0.89		0.042
Caucasian	2,046 (62.7)	1,628 (64.1)		187 (60.7)	
East Asian	102 (3.1)	80 (3.2)		16 (5.2)	
Multiracial	745 (22.8)	582 (22.9)		72 (23.4)	
South Asian	78 (2.4)	58 (2.3)		4 (1.3)	
Southeast Asian	82 (2.5)	66 (2.6)		16 (5.2)	
Other	140 (4.3)	94 (3.7)		11 (3.6)	
Unknown	71 (2.2)	31 (1.2)		2 (0.6)	

a Overall CHILD, all CHILD Cohort Study participants; CHILD Study 5y, the subset of subjects for which inhalant atopy status at age 5 years is known; ITS-2, the subset of subjects with processed stool ITS-2 rRNA gene sequencing data; IQR, interquartile range.

b *P* values represent comparison between left and middle columns.

c *P* values represent comparison between middle and right columns. Wilcoxon rank sum test and Fisher’s exact test were used for continuous and categorical variables, respectively.

### Fungal communities shift dramatically over the first year of life.

After sample and sequence filtering, we identified 1,100 unique fungal amplicon sequence variants (ASVs) in the full data set, representing 6 phyla, 22 classes, 54 orders, 129 families, and 175 genera. The most prevalent ASVs detected included those annotated as Saccharomyces cerevisiae (ASV1; 76% of samples), Candida parapsilosis (ASV3; 55% of samples), an unclassified fungus (ASV2; 42% of samples), another Saccharomyces cerevisiae strain (ASV5; 35% of samples), a second unclassified fungus (ASV37; 32% of samples), and Rhodotorula mucilaginosa (ASV19; 28% of samples).

**FIG 1:**
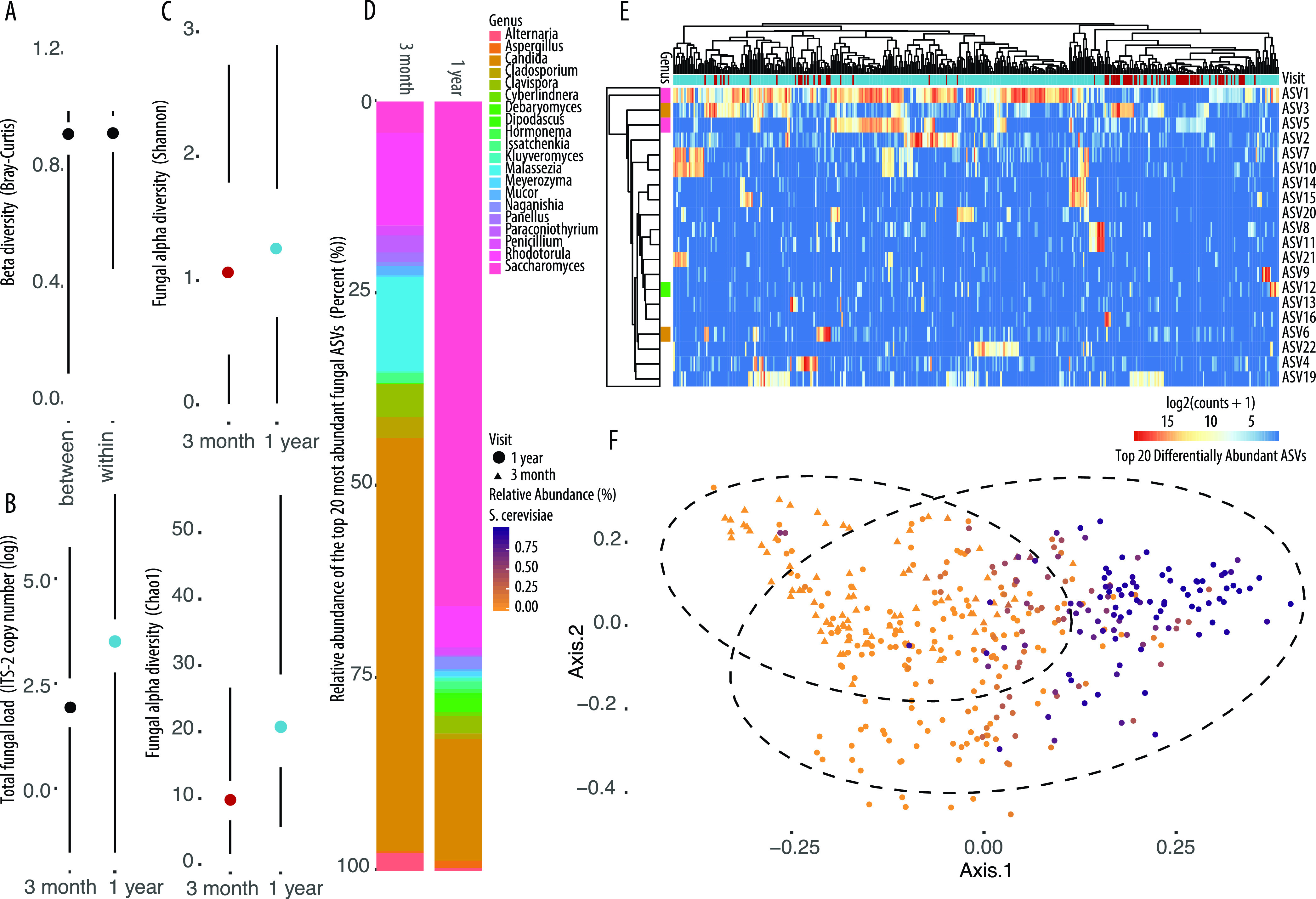
Differences in fungal diversity and total fungal load among stool samples collected at 3 months and 1 year of age from infants in the CHILD Cohort Study determined using ITS-2 amplicon sequencing and qPCR, respectively. (A) Beta diversity within subjects over time is no more similar than beta diversity between samples at the same time point based on Bray-Curtis dissimilarity. (B) Total fungal load in stool at 3 months and 1 year of age shown as ITS-2 copy number on a log scale. (C) Fungal alpha diversity (top, Shannon; bottom, Chao1) at 3 months and 1 year of age. (D) Relative abundances of the top 20 most abundant fungal ASVs annotated to genus level in stool samples collected at 3 months and 1 year of age from infants in the CHILD Cohort Study (ASVs not annotated to the level shown were removed). (E) Heat map of top 20 differentially abundant (by *P* value) ASVs found between 3-month and 1-year stool samples. Genus colors correspond to labels in the key in panel D. (F) Principal-coordinate plot of all samples based on Bray-Curtis dissimilarity of variance stabilized ASV count data and colored according to the relative abundance of Saccharomyces cerevisiae in the sample. Shapes indicate age at sample collection (*R*^2^ = 0.0253; *P* = 0.001). Dots and lines represent the sample mean and range, respectively, in all Tufte plots.

At both 3 months and 1 year of age, infant fungal communities contained few fungal taxa overall (average number of observed ASVs in full data set, 20; at 3 months, 11; at 1 year, 23) and were highly variable across samples. Indeed, fungal communities within infants over time were not more similar than communities between infants (*P* = 0.21 [Fig. 1A]). Despite this variability, shifts in fungal community composition from 3 months to 1 year of age were evidenced by significant increases in total fungal load (Fig. 1B) and in the alpha (Shannon, Chao1, and Faith’s phylogenetic diversity [PD]) diversity of the fungal communities present (Fig. 1C; see also Table S1B in the supplemental material). Moreover, 3-month and 1-year samples could be clearly distinguished in a principal-coordinate analysis (PCoA) plot based on Bray-Curtis dissimilarity (permutational multivariate analysis of variance [PERMANOVA]; *P* = 0.001 [Fig. 1F]).

10.1128/mBio.03396-20.8 TABLE S1 (A) Univariate analysis of differences between gut microbiota fungal community composition at 3 months and 1 year of age according to a subset of early-life exposures based on principal-coordinate analysis using unweighted UniFrac and determined by permutational analysis of variance. *R*^2^ and *P* values are adjusted for sequencing batch (batch *R*^2^ = 0.0104; *P* = 0.003 in entire dataset). Single asterisks indicate *P* values of <0.1, and double asterisks indicate *P* values of <0.05. (B) *P* values of univariate analysis of differences between gut microbiota fungal community alpha diversity and total fungal load at 3 months and 1 year of age according to early-life exposures and determined by Wilcoxon rank sum or Kruskal-Wallis test where applicable. Download Table S1, DOCX file, 0.02 MB. Copyright © 2021 Boutin et al. 2021 Boutin et al. https://creativecommons.org/licenses/by/4.0/ This content is distributed under the terms of the Creative Commons Attribution 4.0 International license.

After observing global differences in fungal communities according to infant age at sample collection, we next examined the specific taxa making up the 3-month and 1-year gut mycobiota communities. From 3 months to 1 year of age, fungal communities exhibit striking differences in the taxa dominating in relative abundance (Fig. 1D and Fig. S1). In early infancy (3 months of age), fungal communities are dominated in relative abundance by species of *Candida*, whereas at 1 year of age, *Saccharomyces* becomes the most frequently detected fungus.

10.1128/mBio.03396-20.2 FIG S1 Relative abundance of fungal phyla identified in individual stool samples collected at 3 months (A) and 1 year (B) of age from infants in the CHILD Cohort Study determined using ITS-2 amplicon sequencing. Download FIG S1, PDF file, 0.2 MB. Copyright © 2021 Boutin et al. 2021 Boutin et al. https://creativecommons.org/licenses/by/4.0/ This content is distributed under the terms of the Creative Commons Attribution 4.0 International license.

To determine whether 3-month and 1-year samples contained statistically significant differences in the relative abundance of specific fungi, we used DESeq2 (57). The top 20 differentially abundant (by *P* value) ASVs were visualized in a heat map of log_2_ ASV counts and confirmed the observation that whereas 3-month-old infants harbor an increase in the relative abundance of *Candida* species, 1-year samples contain an increased relative abundance of *Saccharomyces* (Fig. 1E). Multilevel pattern analysis further confirmed these findings, identifying ASV1 (S. cerevisiae), ASV2 (unclassified fungus), and ASV37 (unclassified fungus) as indicator species associated with 1-year samples. It is notable that no indicator species were identified to be specifically associated with 3-month samples, highlighting the high interindividual variability in gut fungal community composition at this time point.

Given the striking differences in fungal community composition observed according to infant age at sample collection, we aimed to establish the importance of fungi in determining a sample’s “microbial age.” To this end, we used a random forest regressor model trained using merged bacterial and fungal sequencing data to predict microbiota age. This model revealed that nine fungal ASVs were among the top 25 most important taxa contributing to determining the age of an infant at the time of sample collection based on the composition of the gut microbiota (Table 2). Notably, S. cerevisiae was the most important ASV contributing to microbiota age prediction among both bacterial and fungal ASVs, and samples on a PCoA plot based on Bray-Curtis dissimilarity clearly separated along the first axis according to the relative abundance of S. cerevisiae in the sample (Fig. 1F). Together, these data indicate that S. cerevisiae may act as a keystone or marker taxon of particular importance in determining the overall state of the gut mycobiota over time.

**TABLE 2:** Fungal ASVs identified to be among the top 25 most important taxa contributing to determining the age of a sample according to the bacterial and fungal organisms present in the CHILD Cohort Study^*a*^

Fungal ASV taxonomy	Prevalence (%)	Rank of importance for age prediction
ASV1: k__Fungi;p__Ascomycota;c__Saccharomycetes;o__Saccharomycetales;f__Saccharomycetaceae;g__Saccharomyces;s__cerevisiae	76	1
ASV 49: k__Fungi;p__Ascomycota;NA;NA;NA;NA;NA	28	3
ASV 52: k__Fungi;p__Ascomycota;c__Dothideomycetes;o__Capnodiales;f__Cladosporiaceae;g__Cladosporium;s__delicatulum	24	4
ASV 37: k__Fungi;NA;NA;NA;NA;NA;NA	32	5
ASV 27: k__Fungi;p__Ascomycota;NA;NA;NA;NA;NA	23	9
ASV 7: k__Fungi;p__Ascomycota;NA;NA;NA;NA;NA	28	15
ASV 14: k__Fungi;p__Ascomycota;NA;NA;NA;NA;NA	10	22
ASV 6: k__Fungi;p__Ascomycota;c__Saccharomycetes;o__Saccharomycetales;f__Saccharomycetales_fam_Incertae_sedis;g__Candida;s__albicans	23	23
ASV 3: k__Fungi;p__Ascomycota;c__Saccharomycetes;o__Saccharomycetales;f__Saccharomycetales_fam_Incertae_sedis;g__Candida;s__parapsilosis	55	25

a Prevalence of each ASV among samples included in the final fungal ASV table used in the present study (filtered to remove low-abundance taxa and samples with fewer than 1,000 reads) is also indicated (*n* = 389 samples).

### Fungal community functions differ according to infant age.

Functional redundancy among bacterial taxa in the gut often results in similar functional capacities of bacterial communities that differ in the identity and relative abundances of the specific microbes present (58, 59). To determine whether a similar phenomenon occurs within fungal communities, we performed a PICRUSt2 (60) analysis to infer the metagenomes and functional capacities of the 3-month and 1-year stool sample fungal communities using the MetaCyc database (61). Although nearest-sequenced taxon index (NSTI) values were high (maximum weighted NSTI value per sample = 1.70395 and mean = 0.50054, indicating that study sequences were highly distant from reference sequences used in PICRUSt2), 3-month and 1-year samples differed significantly in the metabolic pathway functions of the fungal communities based on PCoA analysis of Bray-Curtis dissimilarity (PERMANOVA; *P* = 0.001 [Fig. S2A]). We report that at 1 year of age, fungal communities in the infant gut demonstrate an increased capacity for functions related to energy metabolism and a decrease in degradation pathways relative to communities at 3 months of age. These findings may be reflective of environmental/niche-specific changes within the gut related to dietary shifts, which may be relevant to overall gut microbiota community structures (62) but warrants further investigation using metagenomic methods (Fig. S2B and C).

10.1128/mBio.03396-20.3 FIG S2 PICRUSt2-predicted functional capacities of fungal communities found in 3-month and 1-year stool samples from infants in the CHILD Cohort Study. (A) Principal-coordinate plot of samples colored by sample collection time point (*R*^2^ = 0.0671; *P* = 0.001) based on a Bray-Curtis dissimilarity matrix using variance stabilized pathway abundance data. (B and C) DESeq2-identified significantly differentially abundant pathways between 3-month and 1-year gut fungal communities annotated according to the MetaCyc database. (B) Heat map showing the log2 abundance of the top 20 differentially abundant functional pathways between 3-month and 1-year samples. Download FIG S2, PDF file, 0.6 MB. Copyright © 2021 Boutin et al. 2021 Boutin et al. https://creativecommons.org/licenses/by/4.0/ This content is distributed under the terms of the Creative Commons Attribution 4.0 International license.

### Early-life environmental factors explain a small proportion of variation in fungal community composition in 3-month and 1-year stool samples.

Early-life factors have been previously shown to influence bacterial communities within the infant gut microbiota. To determine whether these factors similarly explain a significant proportion of variation in fungal communities at 3 months and 1 year of age, we used a series of tests to initially examine whether these factors influence overall fungal community composition (alpha and beta diversities and total fungal load) at each time point. We then used DESeq2 to identify specific fungi that differ in relative abundance according to environmental exposures. Early-life factors examined included antibiotic exposure in the first year of life, birth mode (vaginal, Caesarean section [C-section] with labor, or C-section without labor), residential area type (rural or urban), older siblings, breastfeeding, perinatal pet exposure, season of birth, study center (city), visible mold exposure in the home, the introduction of solid foods by 3 months of age, antibiotic exposure, and antifungal exposure.

### Beta diversity.

PCoA plots based on pairwise Bray-Curtis dissimilarity distances at each time point and single-exposure PERMANOVA revealed associations between older siblings, season of birth, mold in the home at 3 months, and study center with 3-month gut mycobiota composition, whereas breastfeeding, season of birth, household mold at 3 months, study center, and antifungal use explained a significant portion of variance in beta diversity in 1-year samples using a *P* value threshold of *P* = 0.1 (Table 3). Covariates that were significantly associated with gut mycobiota composition in individual analyses at each time point were then included in a multicomponent PERMANOVA to investigate their relative contribution to the fungal community at each time point. For 3-month stool samples, birth season (*R*^2^ = 0.0451; *P* = 0.041), study center (*R*^2^ = 0.0483; *P* = 0.013), and visible mold in the home at 3 months (*R*^2^ = 0.0174; *P* = 0.040) remained significant. At 1 year of age, antifungal use in the first year of life approached significance (*R*^2^ = 0.00458; *P* = 0.078), and both breastfeeding at 1 year (*R*^2^ = 0.00525; *P* = 0.020) and study center (*R*^2^=0.00163; *P* = 0.0010) were significantly associated with differences in mycobiota community composition. Similar effects were observed when beta diversity was examined based on unweighted UniFrac analysis, although associations between early-life factors and unweighted UniFrac beta diversity were generally weaker (Table S1A).

**TABLE 3:** Univariate analysis of differences between gut microbiota fungal community composition at 3 months and 1 year of age according to early-life exposures based on principal-coordinate analysis using Bray-Curtis dissimilarity and determined by permutational analysis of variance^*a*^

Early-life exposure	3 mo		1 yr	
	*R* ^2^	*P* value	*R* ^2^	*P* value
Birth mode	0.0244	0.54	0.00651	0.52
Antibiotic exposure in first 3 mo of life	0.0122	0.52		
Antibiotic exposure in first yr of life			0.00326	0.44
Area type	0.0134	0.34	0.00426	0.11
Older sibling	0.0162	0.094*	0.00411	0.14
Breastfeeding at 3 mo	0.0108	0.72		
Breastfeeding at 1 yr			0.00505	0.019**
Pet exposure	0.0126	0.58	0.00230	0.98
Birth season	0.0457	0.047**	0.0115	0.068*
Study center	0.0473	0.024**	0.0159	0.0010**
Mold exposure	0.0217	0.006**	0.00416	0.088*
Solid food at 3 mo	0.0131	0.47	0.00251	0.91
Bacterial microbiota Chao1 alpha diversity	0.0119	0.55	0.00493	0.034**
Antifungal use in first 3 mo of life	0.0148	0.19		
Antifungal use in first yr of life			0.000459	0.040**

a *R*^2^ and *P* values are adjusted for sequencing batch (batch *R*^2^ = 0.00921; *P* = 0.001 in entire data set). Single asterisks indicate *P* values of <0.1, and double asterisks indicate *P* values of <0.05. *R*^2^ for visit = 0.0253; *P* = 0.0010.

### Alpha diversity and total fungal load.

**FIG 2:**
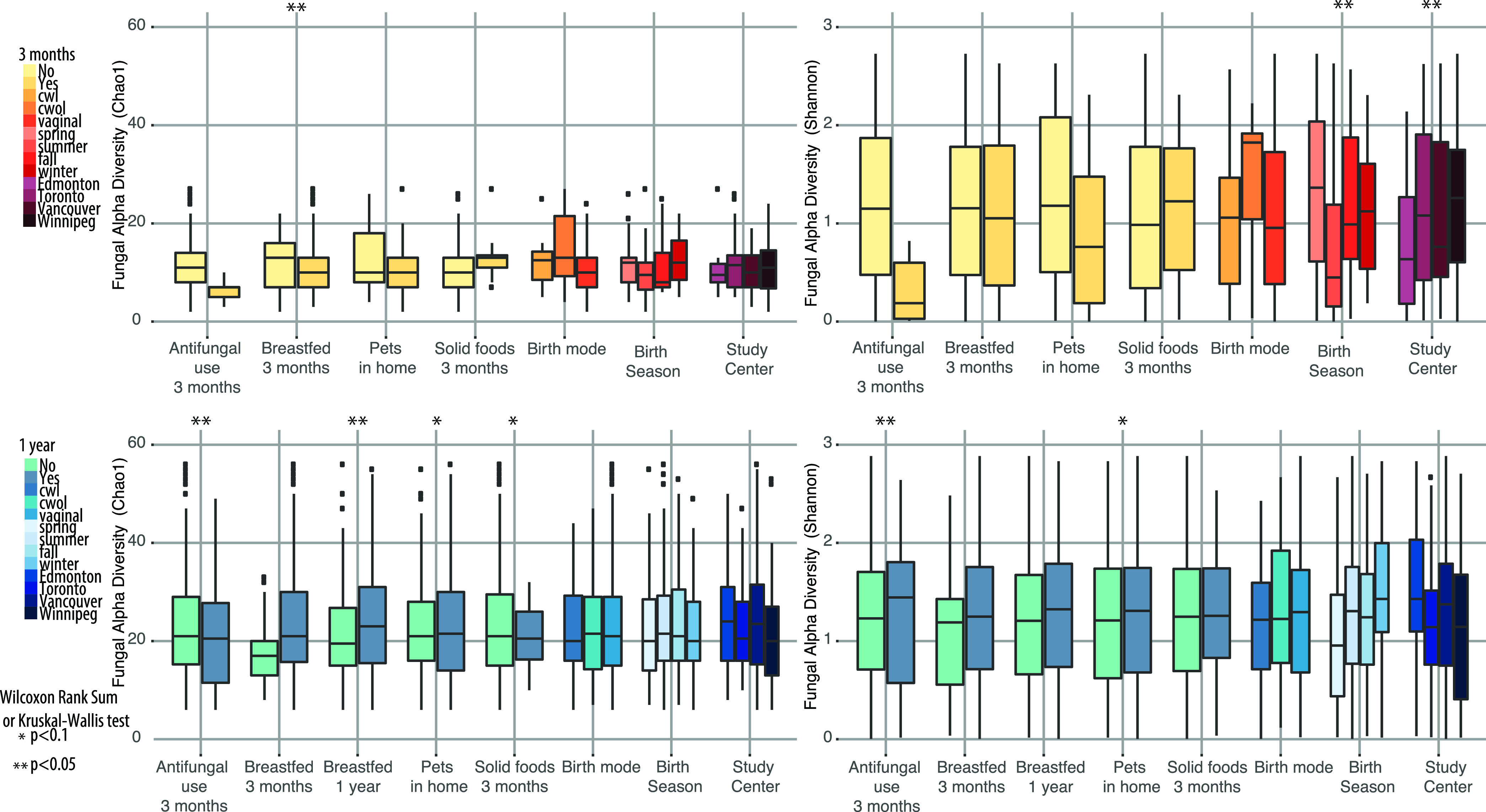
Boxplots showing differences in fungal alpha diversity (Chao1) of the gut mycobiota at 3 months (top) and 1 year (bottom) of age in the CHILD Cohort according to antifungal use, breastfeeding status, pet exposure, solid food at 3 months, birth mode, birth season, and study center. cwl, Caesarean section with labor; cwol, Caesarean section without labor.

We next examined associations between early-life factors and alpha diversity of the gut mycobiota at 3 months and 1 year of age using Chao1, Shannon diversity, and Faith’s PD. At 3 months of age, birth mode trended toward being associated with differences in Faith’s PD of the gut mycobiota (Fig. S3A). Vaginal delivery, perinatal exposure to pets, and lack of solid foods at 3 months were associated with trends toward reduced fungal alpha diversity in the 3-month gut mycobiota (Fig. 2 and Fig. S3B). Antifungal use was also associated with decreased fungal alpha diversity at 3 months (Fig. 2 and Fig. S3B). At 1 year of age, breastfeeding significantly increased the alpha diversity of fungi in the gut (Fig. 2 and Fig. S3B), and both birth season (Fig. 2 and Fig. S3B) and study center (Fig. 2 and Fig. S3B) were associated with gut mycobiota alpha diversity. All other early-life factors were not significantly associated with differences in alpha diversity (Table S1B).

10.1128/mBio.03396-20.4 FIG S3 (A) Tufte plots showing differences in fungal alpha diversity (Faith’s phylogenetic diversity [PD]) of the gut mycobiota at 3 months and 1 year of age in the CHILD Cohort according to birth mode, antibiotic exposure before 3 months of age, antibiotic exposure before 1 year of age, area type, having older siblings, breastfeeding status at 3 months of age, breastfeeding status at 1 year of age, pet exposure, season of birth, study center, solid food at 3 months, mold in the home at 3 months, antifungal use before 3 months of age, and antifungal use before 1 year of age. Cwl, Caesarean section with labor; cwol, Caesarean section without labor. (B) Tufte plots showing differences in fungal alpha diversity (Shannon diversity) of the gut mycobiota at 3 months and 1 year of age in the CHILD Cohort according to birth mode, antibiotic exposure before 3 months of age, antibiotic exposure before 1 year of age, area type, having older siblings, breastfeeding status at 3 months of age, breastfeeding status at 1 year of age, pet exposure, season of birth, study center, solid food at 3 months, mold in the home at 3 months, antifungal use before 3 months of age, and antifungal use before 1 year of age. Download FIG S3, PDF file, 0.4 MB. Copyright © 2021 Boutin et al. 2021 Boutin et al. https://creativecommons.org/licenses/by/4.0/ This content is distributed under the terms of the Creative Commons Attribution 4.0 International license.

Similarly, total fungal load showed little variation according to early-life environmental factors at each time point (Table S1B and Fig. S4) but was significantly increased in 3-month stool samples in infants whose homes contained visible mold at the time of stool sample collection and trended toward being increased in infants with older siblings. Surprisingly, antifungal use in the first 3 months of life was also associated with increased total fungal load in 3-month samples (Fig. S4). At 1 year, children with older siblings tended to have a lower fungal load, whereas breastfeeding was associated with an increased fungal load (Table S1B and Fig. S4). As expected, fungal load and fungal alpha diversity (Chao1) were positively correlated (*R*^2^ = 0.064; *P* = 4.1e−07).

10.1128/mBio.03396-20.5 FIG S4 Differences in total fungal load in stool samples collected at 3 months and 1 year of age in the CHILD Cohort according to environmental factors and health outcomes. The *y* axis indicates ITS-2 copy number determined using qPCR and is on a log scale. Download FIG S4, PDF file, 0.2 MB. Copyright © 2021 Boutin et al. 2021 Boutin et al. https://creativecommons.org/licenses/by/4.0/ This content is distributed under the terms of the Creative Commons Attribution 4.0 International license.

Since few differences were observed in global diversity measures of the gut mycobiota according to environmental factors, we next used DESeq2 to identify whether the relative abundances of specific microbes differed among groups. At both 3 months and 1 year of age, all early-life environmental factors examined were associated with significant differences in the relative abundances of specific taxa found in the infant gut (Text S1). Unsurprisingly given their age-associated dominance, the most frequently affected taxa were ASVs annotated as *Candida*, *Malassezia*, *Debaryomyces*, and *Saccharomyces*. Notably, living in an urban setting was the only variable associated almost exclusively with increases in the relative abundance of certain taxa, whereas very few or no taxa were depleted in association with urban living at both time points.

10.1128/mBio.03396-20.1 TEXT S1 Log fold change of fungal ASVs identified to show significant (false-discovery rate < 0.05) differences in relative abundance in 3-month and 1-year stool samples from infants in the CHILD Cohort Study according to the indicated environmental factor using DESeq2. Error bars indicate standard errors, and taxonomic annotations of ASVs are shown on the *y* axis. Page 1, 1-year stool, asthma at age 5 years versus no asthma at age 5 years. Page 2, 3-month stool, asthma at age 5 years versus no asthma at age 5 years. Page 3, 1-year stool, antifungal use in first year of life versus no antifungal use. Page 4, 3-month stool, antifungal use in first 3 months of life versus no antifungal use. Page 5, 1-year stool, urban residence versus rural residence. Page 6, 3-month stool, urban residence versus rural residence. Page 7, 1-year stool, antibiotic use in first year of life versus no antibiotic use. Page 8, 3-month stool, antibiotic use in first year of life versus no antibiotic use. Page 9, 3-month stool, antibiotic use in first 3 months of life versus no antibiotic use. Page 10, 1-year stool, atopy at age 5 years versus no atopy at age 5 years. Page 11, 3-month stool, atopy at age 5 years versus no atopy at age 5 years. Page 12, 3-month stool, breastfeeding at age 3 months versus no breastfeeding at age 3 months. Page 13, 1-year stool, breastfeeding at age 1 year versus no breastfeeding at age 1 year. Page 14, 1-year stool, C-section delivery versus vaginal delivery without labor. Page 15, 3-month stool, C-section delivery versus vaginal delivery without labor. Page 16, 1-year stool, inhalant allergen sensitization at age 5 years versus no inhalant allergen sensitization at age 5 years. Page 17, 3-month stool, inhalant allergen sensitization at age 5 years versus no inhalant allergen sensitization at age 5 years. Page 18, 1-year stool, mold in the home versus no mold in the home. Page 19, 3-month stool, mold in the home versus no mold in the home. Page 20, 1-year stool, older siblings versus no older siblings. Page 21, 3-month stool, older siblings versus no older siblings. Page 22, 1-year stool, pets versus no pets. Page 23, 3-month stool, pets versus no pets. Page 24, 1-year stool, summer birth versus spring birth. Page 25, 3-month stool, summer birth versus spring birth. Page 26, 1-year stool, fall birth versus spring birth. Page 27, 3-month stool, fall birth versus spring birth. Page 28, 1-year stool, winter birth versus spring birth. Page 29, 3-month stool, winter birth versus spring birth. Page 30, 1-year stool, solid foods at 3 months versus no solid foods at 3 months. Page 31, 3-month stool, solid foods at 3 months versus no solid foods at 3 months. Page 32, 1-year stool, Toronto versus Edmonton. Page 33, 3-month stool, Toronto versus Edmonton. Page 34, 1-year stool, Vancouver versus Edmonton. Page 35, 3-month stool, Vancouver versus Edmonton. Page 36, 1-year stool, Winnipeg versus Edmonton. Page 37, 3-month stool, Winnipeg versus Edmonton. Page 38, 1-year stool, thrush at age 1 year versus no thrush. Download Text S1, PDF file, 0.1 MB. Copyright © 2021 Boutin et al. 2021 Boutin et al. https://creativecommons.org/licenses/by/4.0/ This content is distributed under the terms of the Creative Commons Attribution 4.0 International license.

### Fungal communities in the 3-month-old infant gut associate with inhalant atopy at age 5 years.

In the case of early-onset, allergic asthma, the majority of patients with severe disease exhibit sensitization to inhaled allergens (51). Sensitization (IgE-mediated immune responses) to allergens can be objectively and quantitatively assessed by skin prick testing (sensitization) to inhalant allergens at age 5 years, when asthma cannot be reliably diagnosed by objective measurements of lung function, and is thus a relevant clinical endpoint to assess when considering the relationship between fungal community composition and atopic disease/allergic asthma. In the CHILD Cohort Study, we observed that children with atopy (any positive skin prick test) at age 5 years were predominantly sensitized to inhalant, rather than food, allergens. Overall, children with inhalant atopy at age 5 years were predominantly male, more often born to atopic mothers, exposed to antibiotics in the first year of life, and born by C-section (Table 4 and Table S2A and B).

**TABLE 4:** Demographic and clinical characteristics of CHILD cohort subjects with and without inhalant atopy (inhalant allergen sensitization) at age 5 years^*a*^

Variable	Cohort	Inhalant atopy	No inhalant atopy	*P* value
No. of patients	2,539	438	2,101	
Institution, no. (%)				<0.001
Edmonton	544 (21.4)	89 (20.3)	455 (21.7)	
Toronto	519 (20.4)	136 (31.1)	383 (18.2)	
Vancouver	601 (23.7)	147 (33.6)	454 (21.6)	
Winnipeg	875 (34.5)	66 (15.1)	809 (38.5)	
Antibiotic use by age 3 mo, no. (%)				0.055
	452 (17.8%)	64 (14.6%)	388 (18.5%)	
Antibiotic use by age 1 yr, no. (%)				0.039
	451 (17.8)	93 (21.2)	358 (17)	
Antifungal use by 3 mo, no. (%)				0.22
Confirmed	24 (0.9)	3 (0.7)	21 (1)	
Unknown	2,231 (87.9)	361 (82.4)	1,870 (89)	
Antifungal use by age 1 yr, no. (%)				0.15
Confirmed	26 (1)	3 (0.7)	23 (1.1)	
Unknown	2231 (87.9)	361 (82.4)	1,870 (89)	
Gestational age at delivery (wks)				0.76
Median (range)	39.7 (34, 42.9)	39.7 (34.3, 42)	39.7 (34, 42.9)	
IQR (Q1, Q3)	38.9, 40.6	38.9, 40.4	38.9, 40.6	
Unknown, no. (%)	127 (5)	16 (3.7)	111 (5.3)	
Mode of delivery, no. (%)				0.0011
Vaginal	1,879 (74)	294 (67.1)	1,585 (75.4)	
C-section with labor	324 (12.8)	69 (15.8)	255 (12.1)	
C-section without labor	306 (12.1)	70 (16)	236 (11.2)	
Unknown	30 (1.2)	5 (1.1)	25 (1.2)	
Having older sibling, no. (%)				0.34
Confirmed	1,206 (47.5)	198 (45.2)	1,008 (48)	
Unknown	45 (1.8)	9 (2.1)	36 (1.7)	
Male, no. (%)				<0.001
	1,350 (53.2)	285 (65.1)	1,065 (50.7)	
Birth wt Z score				0.17
Median (range)	−0.1 (−5.9, 15.8)	−0.1 (−5.9, 14.9)	−0.1 (−3.1, 15.8)	
IQR (Q1, Q3)	−0.7, 0.6	−0.7, 0.5	−0.7, 0.6	
Unknown, no. (%)	127 (5)	16 (3.7)	111 (5.3)	
Parental atopy, no. (%)				<0.001
Confirmed	1,997 (78.7)	384 (87.7)	1,613 (76.8)	
Unknown	68 (2.7)	11 (2.5)	57 (2.7)	
Duration of exclusive breastfeeding (mo)				0.33
Median (range)	4 (0, 9)	4 (0, 6)	4 (0, 9)	
IQR (Q1, Q3)	0.5, 5	0.5, 5	0.5, 5	
Unknown, no. (%)	62 (2.4)	7 (1.6)	55 (2.6)	
Tobacco smoke exposure to age 1 yr, no. (%)				0.029
Confirmed	610 (24)	89 (20.3)	521 (24.8)	
Unknown	276 (10.9)	43 (9.8)	233 (11.1)	
Season of birth, no. (%)				0.89
Spring	711 (28)	118 (26.9)	593 (28.2)	
Summer	624 (24.6)	108 (24.7)	516 (24.6)	
Fall	581 (22.9)	106 (24.2)	475 (22.6)	
Winter	621 (24.5)	106 (24.2)	515 (24.5)	
Unknown	2 (0.1)		2 (0.1)	
Area type, no. (%)				<0.001
Rural	74 (2.9)	3 (0.7)	71 (3.4)	
Urban	775 (30.5)	171 (39)	604 (28.7)	
Unknown	1,690 (66.6)	264 (60.3)	1,426 (67.9)	
Breastfeeding status at 3 mo, no. (%)				0.72
None	334 (13.2)	60 (13.7)	274 (13)	
Partial	657 (25.9)	118 (26.9)	539 (25.7)	
Exclusive	1,528 (60.2)	256 (58.4)	1,272 (60.5)	
Unknown	20 (0.8)	4 (0.9)	16 (0.8)	
Breastfeeding status at 12 mo, no. (%)				0.42
Confirmed	1,164 (45.8)	195 (44.5)	969 (46.1)	
Unknown	97 (3.8)	13 (3)	84 (4)	
Solid food by age 3 mo, no. (%)				0.92
Confirmed	173 (6.8)	29 (6.6)	144 (6.9)	
Unknown, n (%)	126 (5)	20 (4.6)	106 (5)	
Presence of mold in home, no. (%)				<0.001
Confirmed	979 (38.6)	214 (48.9)	765 (36.4)	
Unknown	13 (0.5)	2 (0.5)	11 (0.5)	
Oral thrush by age 1 yr, no. (%)				0.81
Confirmed	29 (1.1)	4 (0.9)	25 (1.2)	
Unknown	345 (13.6)	53 (12.1)	292 (13.9)	
Ethnicity of child, no. (%)				<0.001
Caucasian	1,628 (64.1)	236 (53.9)	1,392 (66.3)	
East Asian	80 (3.2)	28 (6.4)	52 (2.5)	
Multiracial	582 (22.9)	128 (29.2)	454 (21.6)	
South Asian	58 (2.3)	12 (2.7)	46 (2.2)	
Southeast Asian	66 (2.6)	21 (4.8)	45 (2.1)	
Other	94 (3.7)	12 (2.7)	82 (3.9)	
Unknown	31 (1.2)	1 (0.2)	30 (1.4)	

a Wilcoxon rank sum test and Fisher’s exact test were used for continuous and categorical variables, respectively.

10.1128/mBio.03396-20.9 TABLE S2 (A) Demographic and clinical characteristics of CHILD cohort subjects with and without inhalant atopy (inhalant allergen sensitization) at age 5 years with available 3-month stool ITS-2 ribosomal DNA (rDNA) sequencing data. Wilcoxon rank sum test and Fisher’s exact test were used for continuous and categorical variables, respectively. (B) Demographic and clinical characteristics of CHILD cohort subjects with and without inhalant atopy (inhalant allergen sensitization) at age 5 years with available 1-year stool ITS-2 rDNA sequencing data. Wilcoxon rank sum test and Fisher’s exact test were used for continuous and categorical variables, respectively. Download Table S2, DOCX file, 0.02 MB. Copyright © 2021 Boutin et al. 2021 Boutin et al. https://creativecommons.org/licenses/by/4.0/ This content is distributed under the terms of the Creative Commons Attribution 4.0 International license.

### Beta and alpha diversities.

**FIG 3:**
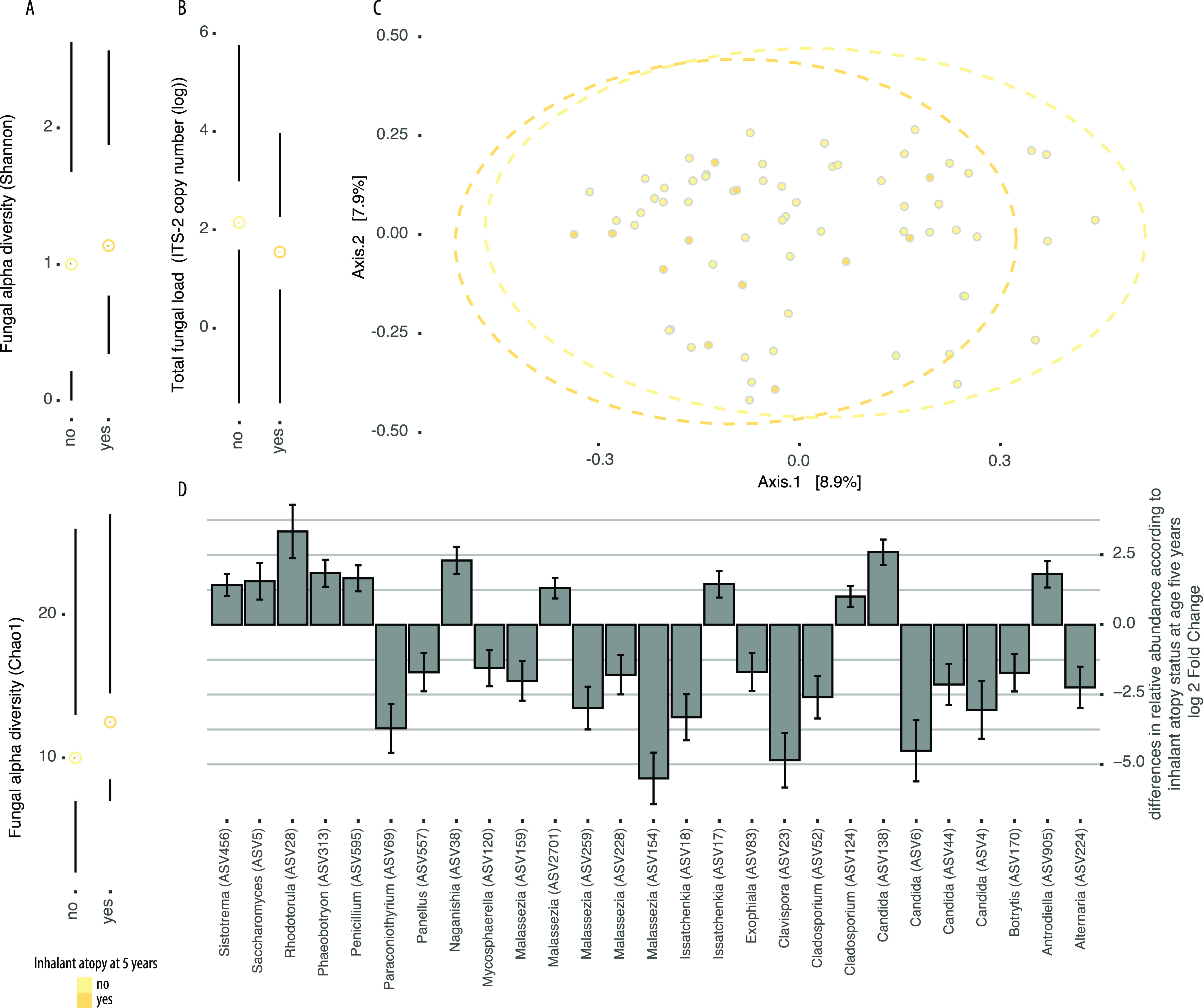
Differences in gut mycobiota communities in stool samples collected at 3 months of age among infants in the CHILD Cohort Study who developed inhalant atopy at age 5 years or remained healthy. (A) Fungal alpha diversity (top: Shannon; bottom: Chao1). (B) Total fungal load shown as ITS-2 copy number on a log scale. (C) Principal-coordinate plot of samples based on Bray-Curtis dissimilarity of variance stabilized ASV count data. (D) Log fold change of fungal ASVs identified to show significant (false-discovery rate < 0.05) differences in relative abundance according to inhalant atopy status at age 5 years determined using DESeq2. Error bars indicate standard errors, and taxonomic annotations of ASVs are shown on the *y* axis. Positive numbers indicate that ASV was increased in abundance in cases relative to controls. Dots and lines represent the sample mean and range, respectively, in all Tufte plots.

**FIG 4:**
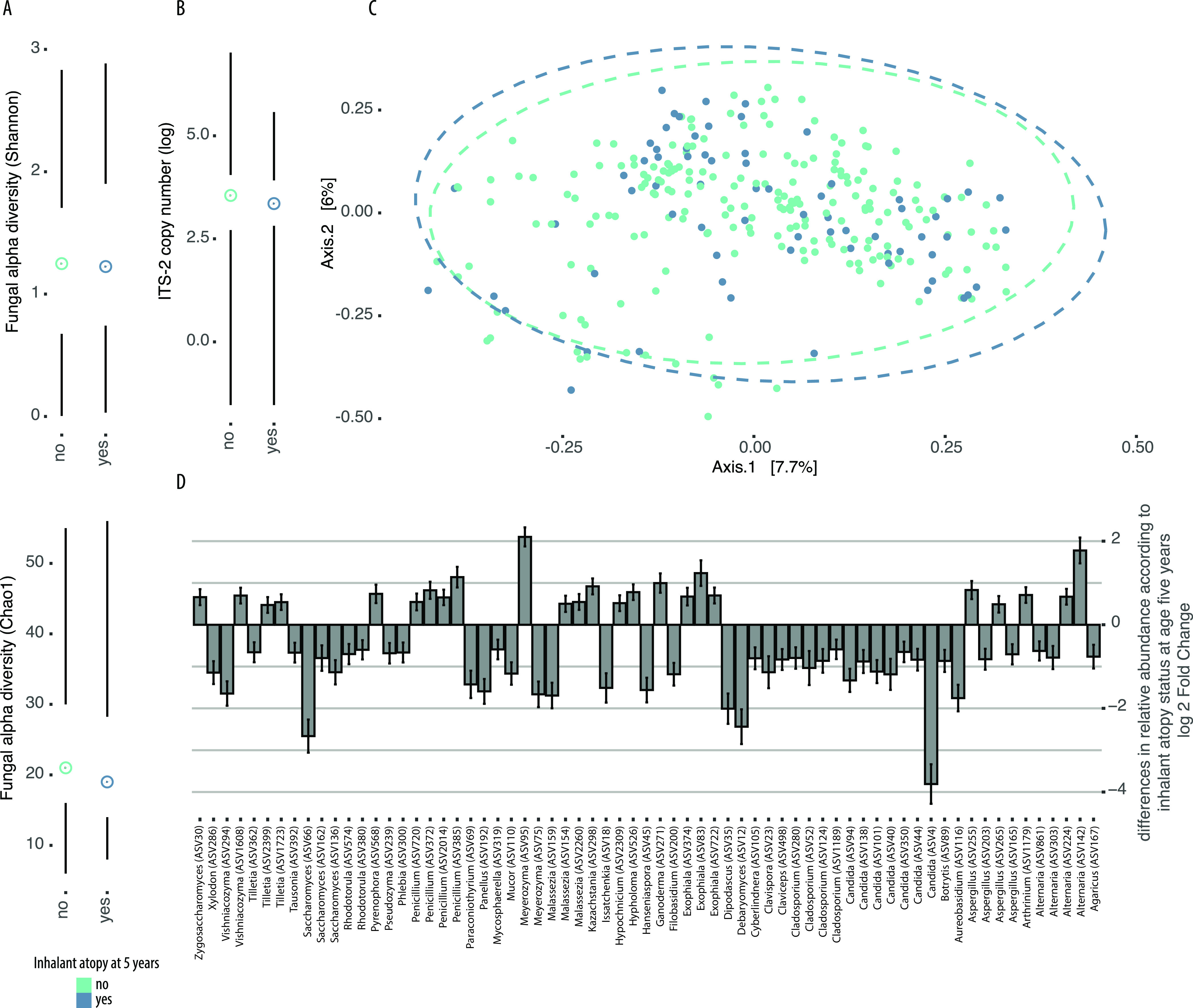
Differences in gut mycobiota communities in stool samples collected at 1 year of age among infants in the CHILD Cohort Study who developed inhalant atopy at age 5 years or remained healthy. (A) Fungal alpha diversity (top: Shannon; bottom: Chao1). (B) Total fungal load shown as ITS-2 copy number on a log scale. (C) Principal coordinate plot of samples based on Bray-Curtis dissimilarity of variance stabilized ASV count data. (D) Log fold change of fungal ASVs identified to show significant (false-discovery rate < 0.05) differences in relative abundance according to inhalant atopy status at age 5 years determined using DESeq2. Error bars indicate standard errors, and taxonomic annotations of ASVs are shown on the *y* axis. Positive numbers indicate that ASV was increased in abundance in cases relative to controls. Dots and lines represent the sample mean and range, respectively, in all Tufte plots.

Significant changes in beta diversity of the mycobiota in 3-month stool samples was associated with sensitization to inhalant allergens at age 5 years (Fig. 3C and Table 5), but this relationship was not seen with 1-year samples (Fig. 4C, Table 5, and Table S3). Gut mycobiota community alpha diversities were also associated differently with health outcomes according to the time of fecal sample collection (Fig. 3 and 4, Table 6, and Fig. S5 and S6). At 3 months of age, increased alpha diversity trended toward being associated with inhalant atopy (Chao1, *P* = 0.16; Shannon, *P* = 0.21) (Fig. 3A and B and Table 6), whereas decreased alpha diversity in 1-year samples was associated with inhalant atopy (Fig. 4A and B). Total fungal load was not significantly associated with health outcomes (Fig. 3B, Fig. 4B, and Table 6).

**TABLE 5:** Univariate analysis of differences between gut microbiota fungal community composition at 3 months and 1 year of age according to health outcomes assessed at age 5 years based on principal-coordinate analysis using Bray-Curtis dissimilarity and determined by permutational analysis of variance^*a*^

Health outcome	3 mo		1 yr	
	*R* ^2^	*P* value	*R* ^2^	*P* value
Inhalant atopy	0.0215	0.024**	0.00425	0.19
Atopy	0.0232	0.0070**	0.00444	0.13
Asthma	0.0120	0.71	0.00512	0.040**

a *R*^2^ and *P* values are adjusted for sequencing batch (batch *R*^2^ = 0.00921; *P* = 0.001 in entire data set). Single asterisks indicate *P* values of <0.1, and double asterisks indicate *P* values of <0.05.

**TABLE 6:** *P* values of univariate analysis of differences between gut microbiota fungal community alpha diversity and total fungal load at 3 months and 1 year of age according to health outcomes assessed at age 5 years and determined by Wilcoxon rank sum test^*a*^

Health outcome	Chao1		Shannon		Faith’s phylogenetic diversity		Total fungal load	
	3 mo	1 yr	3 mo	1 yr	3 mo	1 yr	3 mo	1 yr
Inhalant atopy	0.16	0.20	0.21	0.66	0.87	0.63	0.10	0.46
Atopy	0.53	0.028**	0.78	1	0.89	0.28	0.32	0.22
Asthma	0.32	0.07*	0.80	0.43	0.69	0.99	0.40	0.27

a Single asterisks indicate *P* values of <0.1, and double asterisks indicate *P* values of <0.05.

10.1128/mBio.03396-20.6 FIG S5 Differences in gut mycobiota communities in stool samples collected at 3 months of age among infants in the CHILD Cohort study who developed asthma at age 5 years or remained healthy. (A) Fungal alpha diversity. (B) Total fungal load shown as ITS-2 copy number on a log scale. (C) Principal-coordinate plot of samples based on Bray-Curtis dissimilarity of variance stabilized ASV count data. (D) Log fold change of fungal ASVs identified to show significant (false-discovery rate < 0.05) differences in relative abundance according to asthma status at age 5 years determined using DESeq2. Error bars indicate standard errors, and taxonomic annotations of ASVs are shown on the *y* axis. Positive numbers indicate that ASV was increased in abundance in cases relative to controls. Dots and lines represent the sample mean and range, respectively, in all Tufte plots. Download FIG S5, PDF file, 0.5 MB. Copyright © 2021 Boutin et al. 2021 Boutin et al. https://creativecommons.org/licenses/by/4.0/ This content is distributed under the terms of the Creative Commons Attribution 4.0 International license.

10.1128/mBio.03396-20.7 FIG S6 Differences in gut mycobiota communities in stool samples collected at 1 year of age among infants in the CHILD Cohort study who developed asthma at age 5 years or remained healthy. (A) Fungal alpha diversity. (B) Total fungal load shown as ITS-2 copy number on a log scale. (C) Principal-coordinate plot of samples based on Bray-Curtis dissimilarity of variance stabilized ASV count data. (D) Log fold change of fungal ASVs identified to show significant (false-discovery rate < 0.05) differences in relative abundance according to asthma status at age 5 years determined using DESeq2. Error bars indicate standard errors, and taxonomic annotations of ASVs are shown on the *y* axis. Positive numbers indicate that ASV was increased in abundance in cases relative to controls. Dots and lines represent the sample mean and range, respectively, in all Tufte plots. Download FIG S6, PDF file, 0.7 MB. Copyright © 2021 Boutin et al. 2021 Boutin et al. https://creativecommons.org/licenses/by/4.0/ This content is distributed under the terms of the Creative Commons Attribution 4.0 International license.

10.1128/mBio.03396-20.10 TABLE S3 Univariate analysis of differences between gut microbiota fungal community composition at 3 months and 1 year of age according to health outcomes assessed at age 5 years based on principal-coordinate analysis using unweighted UniFrac and determined by permutational analysis of variance. *R*^2^ and *P* values are adjusted for sequencing batch (batch R^2^ = 0.0104; *P* = 0.003 in entire dataset). Single asterisks indicate *P* values of <0.1, and double asterisks indicate *P* values of <0.05. Download Table S3, DOCX file, 0.01 MB. Copyright © 2021 Boutin et al. 2021 Boutin et al. https://creativecommons.org/licenses/by/4.0/ This content is distributed under the terms of the Creative Commons Attribution 4.0 International license.

### Differentially abundant ASVs.

To next identify specific ASVs associated with inhalant atopy and/or asthma at age 5 years, we performed an indicator species analysis and identified S. cerevisiae (ASV1) as an indicator of all 1-year samples and 3-month samples from infants who developed inhalant atopy at age 5 years (stat = 0.882; *P* = 0.005), atopy at age 5 years (stat = 0.879; *P* = 0.005), or asthma at age 5 years (stat = 0.892; *P* = 0.005). DESeq2-determined taxa that differed significantly in relative abundance between cases and controls with or without inhalant atopy at age 5 years, respectively, were consistent with and extended these findings (Fig. 3D and Fig. 4D). Specifically, for inhalant atopy at age 5 years, taxa typically associated with 1-year samples were increased in relative abundance in cases relative to controls at 3 months of age, and vice versa. Decreases in the relative abundance of *Malassezia* were especially striking in cases of inhalant atopy relative to controls at 3 months of age, whereas ASVs annotated as *Rhodotorula* and a non-*albicans Candida* were increased in relative abundance in 3-month stool samples from infants who developed inhalant atopy at age 5 years relative to those who did not (Fig. 3D). Furthermore, ASV124 (*Cladosporium*) was consistently increased in relative abundance across atopic outcomes (Fig. 3D and Fig. S5 and S6). At 1 year of age, 168 ASVs differed in relative abundance between samples from children with inhalant atopy at age 5 years and controls and included increases in allergy-associated fungi such as *Alternaria* and decreases in the relative abundance of *Debaryomyces*, *Saccharomyces*, and *Candida* in cases relative to controls (Fig. 4D).

### Environmental factors associated with gut mycobiota composition and fungal communities in the infant gut help to predict infants who develop inhalant atopy at age 5 years.

Finally, to determine whether fungal community composition along with other known factors is associated with atopic outcomes and/or microbiota composition can be used to predict which infants will go on to develop inhalant atopy at age 5 years, we used the fungal ASV table to perform partitioning around medoids (PAM) clustering on all of the samples using Jensen-Shannon distances and the Calinski-Harabasz (CH) index to select the optimum number of clusters (63). Using these methods, samples clustered into four different groups according to their fungal community composition.

**FIG 5:**
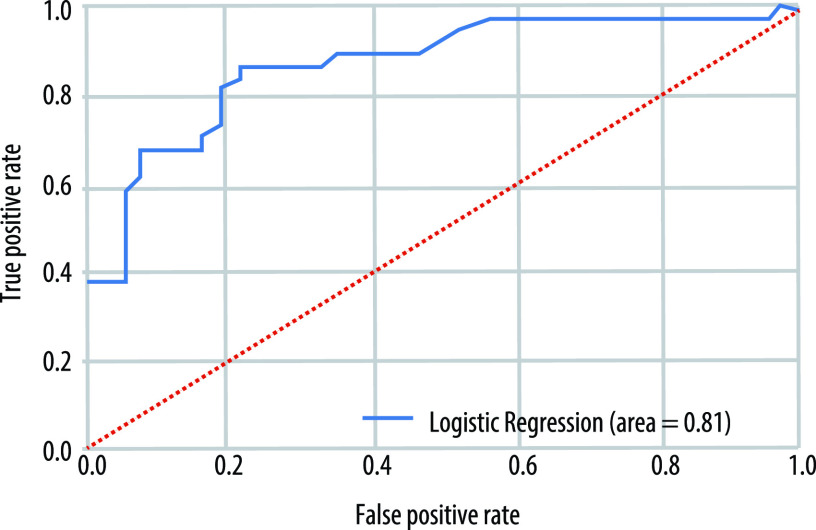
Representative ROC curve of the logistic regression model developed using machine learning for predicting a subject’s inhalant atopy status at age 5 years using information on environmental factors and the composition of the fungal microbiota at 1 year of age.

We then used recursive feature selection and machine learning logistic regression models trained on 70% of the data to determine whether the early-life environmental factors (bacterial Chao1 alpha diversity, antibiotic exposure in the first year of life, birth mode, area type, older siblings, breastfeeding, perinatal pet exposure, season of birth, study center [city], mold exposure in the home, and solid foods at 3 months of age) in combination with knowledge of a subject’s 1-year stool sample PAM cluster could be used to predict whether an infant developed inhalant atopy at age 5 years. This analysis revealed PAM cluster (clusters 2 and 4), antibiotic use, older siblings, season (spring, summer, and winter), and study center (Winnipeg) to be the most important factors for predicting inhalant atopy status at age 5 years. As a predictive model, a logistic regression classifier trained using this information classified samples into the correct class with 80% accuracy (average AUC for 5× cross-validation = 0.80 [Fig. 5]).

To determine whether information on the 3-month stool sample PAM cluster could be used to predict health outcomes despite our small sample size at this time point, we next performed the same modeling approach using only fungal microbiota sequencing information (no environmental or bacterial sequencing data) to determine whether a subject’s PAM cluster at 3 months could be used to predict inhalant atopy status at age 5 years. Using only the 3-month stool sample cluster, we found that the PAM cluster (cluster 1) was associated with inhalant atopy status at age 5 years (*P* = 0.027) and a logistic regression classifier including only information on a sample’s PAM cluster classification (cluster 1) had an accuracy of 78%. Future work should consider imputing missing values, thereby increasing the number of sample points, which would allow us to consider more independent variables as well as PAM cluster at both 3 months and 1 year of age.

## DISCUSSION

### Fungal community composition over the first year of life.

To date, only a limited number of studies have begun to characterize the infant gut mycobiota (22–25, 27, 28), and even fewer studies have examined the bacterial and fungal communities of the gut microbiota simultaneously at multiple time points within the first year of life. Given the instability of the gut microbiota in early life, the importance of colonization succession in determining how this microbial community becomes established (64), and the “critical window” of development during which the composition of the microbiota is suggested to have important consequences for immune development (1), we sought to address some of these unknowns. Our aim was to use data from the CHILD Cohort Study to characterize the composition of the normal and disease-associated infant gut mycobiota at 3 months and 1 year of age.

Despite the high inter- and intraindividual variability observed in gut fungal communities, fungi appear to exhibit an age-dependent shift in community composition in these infants born in Canada. Fungal load and alpha diversity were overall lower than those of infant gut bacteria, but they increased over the first year of life in parallel to bacterial communities. These changes were associated with increases in the relative abundance of S. cerevisiae, which ultimately replaced Candida parapsilosis and Candida albicans as the dominant fungi detected in relative abundance in stool samples from 3 months to 1 year of age. For the first time, we have also observed that shifts in fungal community composition over the first year of life are associated with potential concurrent shifts in fungal community functional capacities.

Similar to a study conducted using data from a birth cohort in Norway (27), we found that a greater proportion of samples collected at 3 months of age had undetectable levels of fungi relative to 1-year samples and that fungal alpha diversity increased over the first year of life. Overall, our findings of ∼20 ASVs per sample, high inter- and intraindividual variabilities in fungal communities, an increased relative abundance of *Candida* at 3 months of age, and dominance of *Saccharomyces* at 1 year of age are consistent with findings in other studies from birth cohorts in industrialized regions such as Norway (27), the United States (24), and Italy (28). However, in contrast to the infants from Norway, for whom 3-month samples were dominated by Debaryomyces hansenii, we found that this organism was increased in relative abundance at 1 year of age relative to 3 months of age, and infants in Italy had a much higher overall relative abundance of *Penicillium* and Aspergillus in the gut than we observed (28). Among other factors, these differences may be due to differences in study sample sizes, the ITS region sequenced, and country-specific breastfeeding practices or other early-life factors.

Notably, the overall fungal community profiles in samples from the first 100 days of life from infants in the CHILD Cohort Study were similar to those in the WHEALS cohort based in Detroit, MI (24), but differed quite strikingly from those of infants in Ecuador. In fact, *Candida* organisms were relatively minor members of the gut mycobiota and *Malassezia* organisms were not even detected among the top 100 operational taxonomic units (OTUs) in Ecuadorian infants when gut mycobiota profiles were characterized using 18S rRNA gene sequencing methods (25). Although sequencing primers and other factors likely played a role in driving these differences between studies, geographical location has been found to be an important factor affecting the presence and composition of breast milk mycobiota (including in the CHILD study) (34, 36), and differences in gut mycobiota composition according to country are consistent with our findings that study center, season of birth, and mold exposure were associated with significant differences in the beta diversity of fungal communities in the guts of Canadian children. Study center, population density, and birth season were also identified to be major factors associated with fungal community presence and composition in human milk samples in the CHILD cohort (36). Climate, time spent outdoors, diet, and cultural practices are likely some of the factors associated with geographical location that influence both what types of microbes are found in certain regions and how infants are exposed to them.

### Fungal communities and early-life environmental exposures/factors.

In addition to geography-related variables, we found that antibiotic exposure in the first year of life, perinatal pet exposure, and breastfeeding were associated with differences in gut mycobiota alpha and/or beta diversity at 3 months and/or 1 year of age. Possibly owing to the low diversity of the samples and highlighting the particular vulnerability of the unstable gut microbiota during the first 100 days of life to environmental influences, all early-life factors examined explained a greater portion of variation (*R*^2^) in beta diversity in 3-month relative to 1-year samples. All early-life factors examined were also associated with differences in the relative abundance of specific fungal taxa at each time point, indicating that environmental exposures may be sources of specific fungi that seed the infant gut. Consistent with studies suggesting vertical transmission of C. albicans from the vaginal tract to the infant gut during vaginal delivery (65), we observed persistent increases in the relative abundance of this microbe in vaginally delivered infants relative to infants delivered by C-section without labor over the first year of life. Although preliminary, these data suggest that early exposure to C. albicans at birth may play an important role in determining the trajectory of gut fungal (and potentially overall gut microbiota) community composition. Indeed, precolonization of the gut with *Candida* has been shown to promote subsequent colonization by certain bacteria (62), and early colonization by these fungi may therefore contribute to the establishment of gut niches necessary for the normal early-life temporal succession of microbial communities necessary for infant health.

Notably, the influences of many of the factors identified to associate with differences in overall fungal community alpha and beta diversities, including having older siblings, often differed according to the age of the infant at the time of sample collection. These results highlight the need to precisely define the age-related immunological consequences of both specific and overall communities of gut fungi over the course of the first year of life. Associations between breastfeeding and gut mycobiota alpha diversity and total fungal load also differed according to an infant’s age at the time of sample collection; at 3 months, breastfeeding decreased alpha diversity and had no effect on total fungal load, whereas at 1 year, breastfeeding increased alpha diversity and total fungal load. Overall and in contrast to many of the other early-life environmental factors examined, breastfeeding was more significantly associated with gut mycobiota communities at 1 year of age, when more dietary heterogeneity existed among subjects, in line with studies showing diet to be a major driver of gut fungal communities (37, 38). These results were further corroborated by observation of a trend toward increased alpha diversity and increased relative abundance of S. cerevisiae in 3-month samples from infants who had already begun to consume solid food. Consistent with our findings, breastfeeding and weaning have been associated in other cohorts with a transition from a Debaryomyces hansenii-dominated mycobiota to a mycobiota dominated by Saccharomyces cerevisiae (27). In piglets, weaning is also associated with an expansion of *Saccharomycetaceae* (66). Given that breast milk mycobiota has been found to be primarily composed of C. albicans, C. parapsilosis, and S. cerevisiae (31), three of the most dominant members of the 3-month (C. albicans and C. parapsilosis) and 1-year (S. cerevisiae) samples in the present study and among that taxa found to differ in relative abundance according to breastfeeding status, it is tempting to speculate that breast milk (or the skin contact involved in the act of breastfeeding) may be an important source of fungi that seed the infant gut. This occurred in the case of bacteria for infants in the CHILD Cohort Study (67) and also for fungi (especially *Candida*, *Alternaria*, and *Rhodotorula*) which were detected in breast milk at 3 months postpartum (36). Whether these breast milk fungi transfer to the infant gut, however, will need to be rigorously tested in future work. Further work is also needed to determine if feeding mode influences gut mycobiota communities, as has been seen in breast milk (36).

Breastfeeding may also promote the growth of certain fungi, as we found that CHILD participants breastfeeding at 1 year of age exhibited an increase in the relative abundance of *D. hansenii*, a food-associated microbe frequently found on dairy products (68) and also found to be associated with breastfeeding in other cohorts (27). In contrast, certain milk components, such as human milk oligosaccharides (HMOs), may inhibit the growth of certain gut fungi, as has been suggested following observations of negative associations between breast milk fungal presence and certain HMOs in breast milk from mothers in the CHILD study (36). These data, together with findings of associations among fungal community composition and both health-associated bacterial community compositions and inflammatory health outcomes, suggest an important role for breastfeeding and/or nutritional practices in determining gut fungal community profiles, with possible consequences for health outcomes.

### Three-month fungal communities and atopic outcomes.

Identifying the makeup of gut mycobiota communities over the first year of life, putative sources of gut fungi, and early-life factors with the potential to shape infant gut mycobiota communities has important therapeutic implications for diseases associated with early-life fungal dysbiosis. Recently, two studies in infant birth cohorts have identified fungal dysbiosis to be an important feature of the dysbiosis-asthma paradigm in nonindustrialized and socioeconomically diverse populations (24, 25). Moreover, antibiotic use is often associated with fungal overgrowth and increased atopy/asthma risk, indicating that interkingdom interactions in the gut may have an underappreciated role in the dysbiosis-asthma/atopy paradigm and that fungal dysbiosis may represent an easily detectable “universal” feature of dysbiosis associated with negative long-term immune consequences. In support of this idea, fungal community beta diversity at 3 months and 1 year was significantly associated with inhalant atopy and asthma, respectively, at age 5 years in the CHILD cohort, whereas such dramatic differences in community beta diversity according to health outcomes have not been observed in the case of bacterial beta diversity in this cohort (44).

A role for fungal dysbiosis in inhalant atopy and allergic asthma is especially intriguing given that fungi are important allergens and share within their surface structures many immunogenic antigens with common inhaled allergens (69–73). Tantalizingly, human Th17 cells generated against common gut commensals were recently shown to be able to cross-react with unrelated fungi in the lungs, causing allergic inflammation (74). Furthermore, we identified several allergy-associated taxa to be enriched in the gut mycobiota of infants with inhalant atopy, including ASVs annotated as *Alternaria* (enriched at 1 year) and *Cladosporium* (enriched at 3 months).

Although our sample size was small and restricts us from drawing broad conclusions, our data suggest that a reduction of the relative abundance of the dominant age-associated fungal genus and/or an increase in fungal diversity in the first 3 months of life increases an infant’s risk of atopic sensitization to inhalant allergens at age 5 years. Notably, infants who developed inhalant sensitization at age 5 years also demonstrated an increase in the relative abundance of S. cerevisiae in 3-month stool samples relative to non-inhalant-atopic controls. S. cerevisiae was also the dominant microbe in 1-year stool samples and was an indicator species associated with microbiota communities from all 1-year-old infants and 3-month-old infants with inhalant sensitization at age 5 years. Surprisingly, knowledge of a subject’s fungal community composition (PAM cluster) at 3 months alone was sufficient to predict inhalant atopy risk at age 5 years with an accuracy of 78%. In stark contrast to bacteria, for which decreased microbiota maturity for age, decreased diversity, and a reduction in the relative abundance of “beneficial” microbes are associated with atopic disease, together these data suggest that premature age-dependent shifts in fungal communities might have negative effects on host immune development. These findings call for a review of the dominant dogma that gut microbiota diversity is widely beneficial; in the case of nonbacterial components of the gut microbiota, the situation may be more nuanced. Moreover, often-overlooked fungi may be especially pertinent early markers of a larger state of gut microbial dysbiosis, including reduced bacterial diversity, occurring prior to the onset of allergic disease manifestations.

In line with our findings of overall gut fungal community composition, we found that features of inhalant atopy-associated fungal dysbiosis before 1 year of age were similar to those identified in the only other study done looking at associations between the mycobiota and childhood atopy in North America (24) but differed from those observed in Ecuadorian infants. In contrast to the increased total fungal load and dramatically increased abundance of Pichia kudriavzevii observed in the 3-month mycobiota of Ecuadorian infants who developed atopy and wheeze at age 5 years, we found that fungal load trended toward being decreased in association with inhalant atopy and that two ASVs annotated as *P. kudriavzevii* (Issatchenkia orientalis) showed different patterns of association with inhalant atopy at age 5 years in 3-month stool samples. These subtle differences between studies could be due to geographical factors as discussed above, different underlying biological mechanisms driving these associations, or differences in sequencing techniques and phenotype definitions, among other factors. Regardless, findings of different signatures of dysbiosis in different regions of the world highlight the need for population-specific studies when designing putative microbiota-based therapeutics or behavioral interventions.

Many of the fungal taxa found to exhibit differential abundances in the 3-month gut microbiota of infants who developed inhalant allergen sensitization at age 5 years relative to controls were also differentially affected by early-life exposures with the potential to influence a child’s contact with microbes. These findings may be used to inform future studies aimed at identifying at-risk infants and testing underlying mechanisms through the use of interventions with potential for therapeutic benefit, and they also suggest that an infants’ risk of atopic disease can be modified by simple, inexpensive behaviors. Introduction of solid foods by 3 months, for instance, was associated with an increase in alpha diversity and the relative abundance of S. cerevisiae in the gut, whereas breastfeeding was associated with trends toward reduced alpha diversity. Exclusive breastfeeding for the first 6 months of life is currently the recommended “gold standard” suggested by the World Health Organization, and modulation of fungal community composition may be a mechanism by which breastfeeding confers health benefits and specifically protects against asthma and allergies, as has been shown previously (75, 76).

### One-year fungal communities and atopic outcomes.

Although many studies have suggested a “critical window” within the first 100 days of life during which the gut microbiota has a unique ability to influence immune-mediated health outcomes, our data suggest a more complex picture when fungi are considered. At 1 year of age, features of fungal dysbiosis were evident in association with inhalant atopy and asthma at age 5 years but differed from the signature of dysbiosis observed at 3 months. Increased fungal load and fungal alpha diversity at 1 year trended toward being protective against atopic disease. Highlighting the importance of precisely defining temporal associations of dysbiosis with health outcomes, the signature of dysbiosis observed at 1 year supports the “hygiene” (43, 77) and “microflora” (78, 79) hypotheses suggesting that reduced microbial exposures (including the microbiota) in early life are associated with atopic disease. Similarly, reduced bacterial alpha diversity was significantly associated with asthma at age 5 years at 1 year, but not 3 months, of age (15). Interventions beyond the first 3 months of life may therefore remain a viable option for meaningfully altering the composition and associated immunomodulatory properties of the gut microbiota.

A primary goal of this work was to determine whether knowledge of fungal community composition and early-life environmental factors associated with both fungal community composition and health outcomes could be used to identify at-risk patients prior to the onset of allergic symptoms. We addressed this question using 1-year samples and a machine learning approach, and we were able to identify 5 features (independent of maternal factors) that predicted an infant’s inhalant atopy status at age 5 years with an accuracy of 81%. Consistent with our findings of early-life factors explaining a significant portion of fungal beta diversity and differences in fungal alpha diversity, an infant’s birth order, breastfeeding status at 1 year, and season and location of birth remained key factors associated with health outcomes at age 5 years in the models. Notably, fungal community composition (PAM cluster), but not bacterial alpha diversity, in the first year of life was a key factor associated with having an allergic diagnosis at age 5 years. Early-life gut fungal community composition may therefore serve as a novel biomarker of later disease onset that can be used to identify at-risk infants and also inform the development of microbiota-based preventative asthma/allergy therapeutics or lifestyle recommendations. Our machine learning model, by taking an unbiased approach, underscores the importance of considering the often-overlooked fungal communities within the infant gut. Fungi may be especially important in the dysbiosis-asthma/atopy paradigm on account of both the robust fungal community at this age and the immunological mechanisms underlying atopic disease.

### Conclusions and future directions.

While our study provides unprecedented insights into fungal communities in the infant gut and how these relate to both health outcomes and bacterial communities, it also has limitations. Some of these limitations are inherent to working with fungi, and others should be addressed in future work. We cannot exclude the possibility that some fungi were not sampled due to their tough cell walls resistant to the DNA extraction method used in this study, primer bias, ITS-2 rRNA gene region length variation, and other methodological factors (21). Some detected fungi were also likely transient passengers in the gut, rather than true colonizers.

Early-life biomarkers of allergy and asthma are currently lacking, preventing the administration of therapeutics or recommendations for lifestyle modifications with the potential to prevent the onset of disease. Future studies in humans with additional and earlier sampling time points, with larger sample sizes, and from populations with diverse demographics are needed to determine whether our findings can be replicated in other cohorts, more precisely define the role of fungal dysbiosis in health and disease, and characterize the timing of important age-dependent shifts in mycobiota community composition. External validation of our machine learning model in other birth cohorts will be essential to determining the generalizability of our findings. With larger sample sizes and metagenomic sequencing, future work should also focus on using more highly sophisticated definitions of “cases” and “controls” for atopic disease that consider the full atopic and genetic history of a subject (56) and specific disease endotypes, as well as whether fungal dysbiosis differentially is associated with sensitization to specific inhaled allergens. Finally, future work using metagenomics sequencing and culture-based methods is needed to validate and further characterize the classification of infant gut fungi at the species and strain levels.

Fungi likely represent an underappreciated component of the microbial biomass within the gut, as tools limited to DNA quantification do not factor total cell size, which are up to 100-fold greater than for bacteria (80). Fungi may affect host immune health either through direct fungal surface antigen- or metabolite-mediated interactions with host immune cells or via indirect effects on immunomodulatory gut bacterial populations. Furthermore, interactions between bacteria and fungi in the gut may have important consequences for successional community dynamics in the infant gut (64) as well as for known time-dependent immune-bacterial microbiota interactions that ultimately determine immune calibration relevant to allergic disease outcomes. Here, we have demonstrated the existence of a dynamic fungal community in the gut microbiota of infants from an industrialized country. We have also shown that fungi influenced by early-life factors, such as diet and mold or antifungal exposure, are associated with inhalant atopy outcomes later in life. Going forward, this raises the exciting possibility of designing/implementing inexpensive behavioral and/or dietary interventions with the potential to influence gut microbiota community composition and long-term health. Altogether, this work lays the foundation for future studies looking to compare gut mycobiotas across birth cohorts and indicates a critical role for fungi as mediators of microbiota-associated changes in immune function. Future work *in vivo* and *in vitro* will seek to validate our findings in animal models, establish a causal role for early-life gut fungal dysbiosis in atopic outcomes, and identify the mechanisms mediating associations between fungal dysbiosis and atopic health outcomes.

## MATERIALS AND METHODS

### Study population.

The CHILD Cohort Study recruited 3,621 pregnant women from four sites (Vancouver, Edmonton, Winnipeg, and Toronto) across Canada (55, 81). Of the 3,621 pregnant mothers recruited to the CHILD study, 216 in a Vanguard cohort were excluded from this analysis due to the change of data collection method. Of the 3,405 recruited to the general cohort, 3,264 eligible infants stayed in the study at birth, had no congenital abnormalities, and were born at a minimum of 34 weeks of gestation. CHILD Cohort Study children were followed prospectively, and detailed information on environmental exposures and clinical measurements and assessments were collected using a combination of questionnaires and in-person clinical assessments at 11 time points from birth until age 5 years.

### Definitions of early-life factors and health outcomes.

**(i) Antibiotic and antifungal use.** Medication data were obtained from questionnaires completed by study families. Drugs were manually curated, assigned standardized generic names, and then linked to ontology terms in the Chemical Entities of Biological Interest database (82, 83). Antibacterial drugs were also linked to high-level antimicrobial resistance ontology drug classes at the Comprehensive Antibacterial Resistance Database (84). Antibiotic use was defined as at least one antibiotic course within the first year of life after hospital discharge, with the exclusion of topical antibiotics (e.g., eye drops, ear drops, and ointment). Antifungal use was defined as at least one systemic antifungal drug use (assessed at the 3-month, 6-month, and 1-year visits). Amoxicillin represented 55% of prescriptions, and the most common antifungal used by study participants was nystatin, at 75% of all systemic antifungals used.

**(ii) Birth mode.** Delivery type was recorded on the birth chart and used to define three modes: vaginal, planned C-section (i.e., without labor), and C-section with labor.

**(iii) Residential area type.** A dichotomous variable for area type, defined as “urban” or “rural” and assigned at the postal code level of each participant residential home, was derived from the 2011 Statistics Canada census following its validated definition whereby all territory within a census metropolitan area (CMA) or census agglomeration (CA) that is not classified as a core (minimum of 50,000 people in CMA) or fringe (minimum of 10,000 people in CA) is classified as rural.

**(iv) Older siblings.** Each mother’s parity was determined from the birth chart and used to determine the birth order of the child. “Yes” was defined as having one or more older siblings.

**(v) Breastfeeding (at 3 months and 1 year of age).** Breastfeeding was defined as none, partial, or exclusive as previously described (75). For the present study, breastfeeding was defined as a dichotomous variable of “any” or “none” by combining partial and exclusive breastfeeding as “any.”

**(vi) Pets.** Pet exposure was defined as “yes” if a child was exposed to either a cat or a dog in pregnancy and/or in the first year of life.

**(vii) Season.** Season was defined based on the date of birth of the child. Spring was defined as March to May, summer was June to August, fall was September to November, and winter was December to February (inclusive).

**(viii) Mold exposure.** Mold exposure (yes/no) was determined from home environment inspection completed by CHILD study staff at the 3-month home visit. Specifically, children were considered to have been exposed to mold if visible mold was recorded anywhere in the home. If no data were collected on mold in the home, exposure was treated as “no” to provide a conservative estimate without losing power.

**(ix) Solid food at 3 months.** Introduction of solid foods (yes/no) by 3 months of age was determined from a nutrition questionnaire administered at 3 months of age.

**(x) Skin prick test (atopy) outcomes.** Children were diagnosed with atopy (IgE-mediated allergic sensitization) based on standardized skin prick testing to food and environmental inhalant allergens at age 5 years. Average wheal sizes of ≥2 mm relative to the negative control (glycerin) were considered to represent a positive test, and histamine was used as a positive control. Inhalant allergens tested included fungi (Alternaria alternata, Aspergillus fumigatus, *Cladosporium*, and *Penicillium*), house dust mites (Dermatophagoides pteronyssinus and Dermatophagoides farinae), cat hair, dog epithelium, pollens (tree mix, grass mix, weed mix and ragweed mix), and German cockroach. Food allergens tested included cow’s milk, peanut, egg white, and soybean. Atopic burden was defined as the weighted average of the skin prick test wheal sizes to inhalant allergens (total sum of all wheal sizes/total number of positive tests).

**(xi) Asthma at age 5 years.** Asthma was diagnosed at age 5 years based on a study subspecialist pediatrician response to the question “In your opinion, does this child have asthma? (Yes/Possible/No)” as previously described (56). This decision was based on a structured interview with the accompanying parent or guardian. Definite asthma (“yes”) was recorded if the parent reported physician-diagnosed asthma, use of a bronchodilator prescribed for episodes of coughing or wheezing, use of a prescribed daily controller medication, or frequent wheezing (three or more distinct episodes over the previous year) with no alternative diagnosis. Possible asthma (“possible”) was recorded if there were less frequent episodes of wheeze or coughing without colds and no report of medication use. In the present study, “no” and “possible” asthma were grouped as noncases.

### Sample collection.

Soiled diapers from infants were collected at a home visit by CHILD Cohort Study staff scheduled at 3 months of life, and an additional stool sample was obtained at the clinical assessment scheduled at age 1 year, as previously described (81).

### DNA extraction and 16S rRNA gene amplicon sequencing and processing.

DNA extraction from stool samples from the CHILD Cohort Study and 16S rRNA gene amplicon sequencing were performed as previously described (15, 56). Sequencing was performed using primers F515/R806 targeting the V4 hypervariable region of the 16S rRNA gene. Paired-end sequencing reads were processed using DADA2 (85) within QIIME2 v.2018.6 (86). Taxonomy was assigned and aligned to the Greengenes reference database (2013 release) (87) at 99% sequence similarity. Downstream filtering and processing of the ASV table was done in R. Filtering steps included removal of samples containing fewer than 1,000 sequences and taxa present at <0.5% relative abundance in at least three samples or <5% relative abundance in at least one sample.

### DNA extraction and ITS-2 rRNA gene amplicon sequencing.

Five hundred forty-five stool samples from 343 subjects were selected for DNA isolation and ITS-2 rRNA gene sequencing based on 16S rRNA gene sequencing data availability from the same sample or subject and stool availability. All samples had a minimum DNA yield of 8 ng/μl following the DNA extraction. Where possible, samples collected at both 3 months (*n* = 206 samples) and 1 year (*n* = 339 samples) of age from the same subject were chosen. DNA was extracted from frozen stool samples using the Qiagen QIAamp PowerFecal DNA extraction kit according to the manufacturer’s instructions, with minor modifications. Briefly, approximately 250 mg of frozen stool was used for each extraction and samples were homogenized twice using an MP Biomedicals FastPrep machine at speed 5.5 for 60 s. DNA from was subjected to 300-bp paired-end sequencing on an Illumina MiSeq using the ITS3/ITS4 primer pair (ITS3, 5′-GCATCGATGAAGAACGCAGC-3′; ITS4, 5′-TCCTCCGCTTATTGATATGC-3′) and V3 chemistry.

### ITS-2 rRNA gene sequencing processing.

Cutadapt was used to trim primers from the sequences and then the DADA2 ITS pipeline workflow (https://benjjneb.github.io/dada2/ITS_workflow.html) was used to process raw sequences from forward reads into amplicon sequence variants (ASVs) within R Studio (88) (v.1.2.5019) and R (v.3.5.1) (89). In order to use a paired-end error model, forward reads were electronically reverse transcribed (preserving the adapters and ensuring that the quality scores were maintained). Sequences with ambiguous bases were removed and the remaining sequences were quality filtered (maximum of expected errors = 2; minimum sequence length = 50 bp) and denoised to generate ASVs. Reads were not trimmed to a fixed length in order to accommodate differences in amplicon length of the ITS-2 region (21). Chimeras were removed in DADA2 and ASV taxonomy was assigned using the UNITE (release_dynamic_02.02.2019) database (90, 91) and a naive Bayesian classifier. The resulting ASV tables and representative sequences from each run were then merged and duplicate samples were removed, generating an ASV table containing 4,221 features in 520 samples (3 months, *n* = 193; 1 year, *n* = 327). Representative sequences were also searched in the SILVA database to ensure that ASVs annotated only to kingdom level did not match to prokaryotic organisms. Key ASVs lacking annotation below kingdom level were further searched using BLAST to improve taxonomic resolution where possible. Extraction and PCR controls contained only 48 to 129 total reads across 7 ASVs and were filtered out of the analysis during subsequent filtering steps.

All subsequent filtering steps were done in R to generate the final table used in downstream analyses. Filtering steps included removal of samples containing fewer than 1,000 sequences and taxa present at <0.05% relative abundance in at least three samples or <0.5% relative abundance in at least one sample. The final table contained 1,100 taxa in 389 samples (3 months, *n* = 81; 1 year, *n* = 308).

*Ghost-tree* was used for computing phylogeny-based diversity metrics for fungal amplicon sequencing data (92) (https://forum.qiime2.org/t/q2-ghost-tree-plugin-community-tutorial-for-creating-hybrid-gene-phylogenetic-trees/6139). Briefly, closed-reference clustering of DADA2-generated ASVs at 99% identity to the UNITE developer reference database (ver8_99_02.02.2019) was done using VSEARCH (93) within QIIME2 (86) v.2019.10 and the resulting table was filtered to contain feature identifiers (IDs) present in the tree ghost_tree_100_qiime_ver8_99_02.02.2019. Clustering was performed against a reference database clustered at the same percent identity according to the QIIME2 developer recommendations. The resulting ASV table was then filtered to remove samples with fewer than 1,000 features and ASVs present in fewer than two samples (112 ASVs in 304 samples retained). The ghost_tree_100_qiime_ver8_99_02.02.2019 was then used for computing phylogenetic metrics after being midpoint rooted in QIIME2.

### ITS-2 and 16S rRNA gene sequencing analysis.

Faith’s phylogenetic diversity (PD), a measure of alpha diversity that reflects the sum of the branch lengths of a phylogenetic tree containing the ASVs found in a sample (94), was computed using the *picante* package (95) in R. Additional alpha (Shannon and Chao1) and beta (unweighted UniFrac [96] and Bray-Curtis) diversities were computed in R using the *phyloseq* (97) and *vegan* (98) packages, respectively. Shannon diversity incorporates information on ASV richness and evenness (99), whereas Chao1 is a nonparametric estimate of community richness that accounts for rare taxa (100). Beta diversities were visualized in principal-coordinate analysis (PCoA) plots based on Bray-Curtis dissimilarity or unweighted UniFrac after variance stabilizing the filtered ASV tables to generate a count matrix of values with constant variance around mean values using DESeq2 (57, 101). Bray-Curtis dissimilarity considers differences between samples based on the relative abundance of ASVs, whereas unweighted UniFrac uses a phylogenetic tree to determine dissimilarities between samples based on the presence/absence of ASVs.

Pattern analysis of associations between “indicator” fungal ASVs and environmental variables or health outcomes were determined using the “multipatt” function in the *indicspecies* package (102), the Indval.g association function (to correct for unequal sample sizes), a significance level of 0.05, and a minimum indicator statistic value cutoff 0.6, as has been used previously (103, 104). Indicator species are used in ecology studies to identify species whose abundance reflects particular community states and can be measured in lieu of measuring the abundance of all species in a community (102). The indicator value is computed by estimating the specificity (i.e., whether a sample belongs to a specified group if the species is found in that sample) and the sensitivity of an indicator species (i.e., most samples in the specified group will contain that species).

### Merging bacterial and fungal sequencing data to predict microbial age.

Filtered bacterial and fungal ASV tables were merged in *phyloseq* and then imported into QIIME2 v.2019.10 to identify bacterial and fungal ASVs contributing most significantly to determining the “age” of an infant according to the composition of the gut microbiota. Samples with fewer than 1,000 ASVs and ASVs present fewer than three times in the merged data set were removed from the analysis. Using the exact date of stool sample collection for each sample (75 to 594 days of life), 60-day bins were then created and passed with the ASV table to the *qiime sample-classifier* (105, 106). This method uses a supervised random forest learning regressor to predict the age of a sample according to the composition of the gut microbiota and provides information on the importance of each ASV to determining a sample’s “microbial age.” Half of the samples were used for training, and 100 estimators were used.

### PICRUSt2 analysis of ITS rRNA gene sequencing data.

The functional capacities of the fungal communities in stool samples collected at 3 months and 1 year of age from infants in the CHILD cohort were investigated using PICRUSt2 (Phylogenetic Investigation of Communities by Reconstruction of Unobserved States) (60) (https://github.com/picrust/picrust2/wiki/Full-pipeline-script). Within PICRUSt2, HMMER (http://hmmer.org/) is used for reference sequence alignment, and then EPA-NG (107) maps sequences to a reference phylogeny. The final object is converted to newick format using GAPPA (108), and the *castor* R package (109) is used for hidden-state prediction of gene family abundances. Finally, MetaCyc (61) and MinPath (110) are used to predict metabolic pathway functions based on enzyme commission numbers.

### Quantitative PCR.

Total fungal load was assessed using the FungiQuant quantitative PCR (qPCR) assay on all samples submitted for ITS-2 rRNA gene sequencing (111). Specifically, sample DNA concentrations were determined by Qubit analysis and concentrations were normalized to 1 ng/μl, 10 ng/μl, or 100 ng/μl. Two microliters of template DNA was added to a reaction mixture containing iTaq universal probe supermix (Bio-Rad), H_2_O, 6-carboxyfluorescein (FAM) probe (1 μM; Applied Biosystems), forward primer (10 μM; GGRAAACTCACCAGGTCCAG), and reverse primer (10 μM; GSWCTATCCCCAKCACGA) for a total reaction volume of 10 μl in 100-μl MicroAmp 96-well plates (Applied Biosystems). This assay uses primers specific for the more highly conserved 18S rRNA gene of the fungal genome, which exhibits less length variability than the ITS-2 region and is therefore more suitable for qPCR assays. Reactions were run in duplicate at standard ramp speed, and qPCR was performed on a 7500 fast real-time system (Applied Biosystems, Foster City, CA) machine using the following cycling protocol: an initial enzyme activation step at 95°C for 2 min followed by 45 cycles of a denaturation (95°C for 15 s) step and then a combined annealing/extension step (60°C for 1 min). Amplicon DNA concentration was determined using a standard curve generated using 10-fold dilutions of a 0.1-ng/μl stock of 18S rRNA gene amplicons purified from a PCR completed using the FungiQuant primers and purified C. parapsilosis template DNA. C. parapsilosis template DNA was extracted from a pure culture of C. parapsilosis (ATCC 22019) grown at 30°C for 24 h using the Quick-DNA fungal/bacterial microprep kit (Zymo Research) kit. Using the calculated DNA concentration, 18S rRNA gene copy number was determined based on the anticipated size of the PCR amplicon (311 bp) and the following formula:

$$\text{number of copies }(\text{molecules})=\frac{X\text{ ng}\times 6.0221\;\times \;10^{23}\text{ molecules}/\text{mole}}{\left(N\times 660\text{ g/mole}\right)\;1\;\times \;10^{9}\text{ng}/\text{g}}$$

where *X* is amount of amplicon, *N* is length of double-stranded DNA (dsDNA) amplicon, and 660 g/mole is average mass of 1 bp of dsDNA.

Any samples with a large discrepancy between duplicate readings were rerun in triplicates. All samples were standardized to 10 ng/μl of total DNA for final comparative analysis. If DNA was not detected or the threshold value (*C_T_*) was above the negative controls, a value of 10^−11^ ng/μl of DNA was used (the limit of detection of the qPCR assay). Any quantity of DNA detected in the negative controls was subtracted from all samples of the corresponding plate.

### Predicting health outcomes using fungal ASV and environmental data.

Fungal ASV data were used to cluster samples based on Jensen-Shannon distance and partitioning around medoids (PAM) clustering using previously described methods (63). The Calinski-Harabasz (CH) index was used to select the optimum number of clusters (63). Recursive feature elimination and machine learning logistic regression models were then used to identify the most important features for predicting a subject’s inhalant atopy or asthma status at age 5 years using the scikit learn machine learning library within Python (106, 112, 113). We also visually inspected the independent variables by plotting their relative frequencies in the sample. Specifically, using PAM cluster at 1 year of age, sample bacterial alpha diversity (Chao1), sex, and the early-life factors of antibiotic exposure in the first year of life, birth mode, area type, older siblings, breastfeeding status, perinatal pet exposure, season of birth, study center (city), mold exposure in the home, and the introduction of solid foods at 3 months of age, the Synthetic Minority Oversampling Technique (SMOTE) (114) algorithm was used for mitigating the imbalance of sample point with respect to the values of the dependent variables. Antifungal use was not included in the model, as only four children with inhalant allergen sensitization and asthma at age 5 years were exposed to antifungals within the first year of life. Due to the limited number of samples available, which was less than 500, compared with the large number of candidate explanatory variables, we applied an algorithmic method to pick the most essential (predictive) variables. Recursive feature elimination, using the feature selection submodule in the scikit learn library, was used to select features that best predicted health outcomes, and 70% of the data were used as the training set. The model accuracy was then calculated using the remaining 30% of the data as the test set and visualized in a receiver operating characteristic (ROC) curve with 5-fold cross-validation, using the roc_curve function in the metrics submodule in the scikit learn library. Using only information on PAM clusters, the same methods were used to determine whether PAM cluster at age 3 months was associated with inhalant atopy at age 5 years.

### Statistical analyses.

Statistical tests were performed in R (version 3.5.1). Alpha diversity metrics were tested for normality using the Shapiro-Wilk normality test, and pairwise group comparisons were then done using a Wilcoxon rank sum test. Multiple-group comparisons were done using a Kruskal-Wallis test based on the distribution of the data.

Associations between environmental factors and health outcomes with fungal beta diversity were compared by permutational analysis of variance (PERMANOVA) using the *adonis* function from the package *vegan* (98) with 999 permutations, and all results are reported as marginal effects after controlling for batch effects.

Differential abundance tests were done using DESeq2 (57), correcting for batch and using a significance threshold of a *P* value of <0.05 after false-discovery rate (FDR) correction. Differences in total fungal load between groups were calculated using the Wilcoxon rank sum (Mann-Whitney) or Kruskal-Wallis test. Only samples used in the final fungal ASV table were used for total fungal load comparisons.
