# Supplementary material for: Composition and Associations of the Infant Gut Fungal Microbiota with Environmental Factors and Childhood Allergic Outcomes
Source: mBio. 2021 Jun 1;12(3):e03396-20. doi: 10.1128/mBio.03396-20 (PMC8263004; doi:10.1128/mBio.03396-20)
Supplement: TABLE S1 [file mbio.03396-20-st001.docx]

Table S1A Univariate analysis of differences between gut microbiota fungal community composition at three months and one year of age according to a subset of early life exposures based on principal coordinate analysis using unweighted Unifrac and determined by permutational analysis of variance. R^2^ and p-values are adjusted for sequencing batch (batch R^2^=0.0104, p=0.003 in entire dataset). Single stars indicate p<0.1, double stars indicate p<0.05.

| **Early life exposure** | **Three months** | | **One year** | |
| --- | --- | --- | --- | --- |
|  | **R^2^** | **p-value** | **R^2^** | **p-value** |
| Birth mode | 0.0340 | 0.44 | 0.00817 | 0.47 |
| Antibiotic exposure in first three months of life | 0.0128 | 0.75 | - | - |
| Antibiotic exposure in first year of life | - | - | 0.00440 | 0.36 |
| Area type | 0.0132 | 0.69 | 0.00488 | 0.28 |
| Older sibling | 0.0245 | 0.17 | 0.00293 | 0.78 |
| Breastfeeding at three months | 0.00995 | 0.83 | - | - |
| Breastfeeding at one year | - | - | 0.00794 | 0.018** |
| Pet exposure | 0.00878 | 0.92 | 0.00532 | 0.24 |
| Birth season | 0.0399 | 0.77 | 0.00972 | 0.84 |
| Study center | 0.0493 | 0.48 | 0.0201 | 0.0090** |
| Mould exposure | 0.0244 | 0.16 | 0.00360 | 0.56 |
| Solid food at three months | 0.0157 | 0.51 | 0.0030 | 0.75 |
| Antifungal use in first three months of life | 0.0621 | 0.001** | - | - |
| Antifungal use in first year of life | - | - | 0.00272 | 0.84 |

*R^2^ for visit=0.0219, p=0.0010

Table S1B. P-values of univariate analysis of differences between gut microbiota fungal community alpha diversity (Chao1, Shannon, Faith’s Phylogenetic Diversity) and total fungal load at three months and one year of age according to early life exposures and determined by Wilcoxon Rank Sum or Kruskal-Wallis test where applicable. Single stars indicate p<0.1, double stars indicate p<0.05.

| **Early life exposure** | **Chao1** | | **Shannon** | | **Faith’s Phylogenetic Diversity** | | **Total fungal load** | |
| --- | --- | --- | --- | --- | --- | --- | --- | --- |
|  | **Three months** | **One year** | **Three months** | **One year** | **Three months** | **One year** | **Three months** | **One year** |
| Visit | 2.2e-16** | | 0.12 | | 0.00013** | | 2.2e-16** | |
| Birth mode | 0.29 | 0.89 | 0.49 | 0.7 | 0.12 | 0.80 | 0.43 | 0.84 |
| Antibiotic exposure | 0.21 | 0.52 | 0.40 | 0.62 | 0.2 | 0.33 | 0.99 | 0.80 |
| Area type | 0.49 | 0.46 | 0.74 | 0.21 | 0.58 | 0.93 | 0.31 | 0.68 |
| Older sibling | 0.74 | 0.28 | 0.55 | 0.56 | 0.33 | 0.87 | 0.066* | 0.097* |
| Breastfeeding at three months | 0.31 | 0.0055** | 0.75 | 0.16 | 0.82 | 0.0014** | 0.85 | 0.053* |
| Breastfeeding at one year | NA | 0.022** | 0.12 | 0.17 | NA | 0.7 | NA | 0.15* |
| Pet exposure | 0.08* | 0.86 | 0.051* | 0.81 | 0.72 | 0.51 | 0.27 | 0.88 |
| Birth season | 0.27 | 0.48 | 0.18 | 0.0039** | 0.61 | 0.70 | 0.26 | 0.81 |
| Study center | 0.85 | 0.17 | 0.58 | 0.036** | 0.61 | 0.57 | 0.52 | 0.92 |
| Mould exposure | 0.48 | 0.61 | 0.70 | 0.71 | 0.96 | 0.59 | 0.0092** | 0.94 |
| Solid food at three months | 0.058* | 0.82 | 0.64 | 0.89 | 0.36 | 0.4 | 0.95 | 0.71 |
| Antifungal use in first three months of life | 0.00052** | 0.59 | 0.0013** | 0.69 | 0.00059** | 0.41 | 0.02** | 0.04** |
| Antifungal use in first year of life | NA | 0.40 | NA | 0.82 | NA | 0.029** | NA | 0.096* |
