# Supplementary figures and images for: Composition and Associations of the Infant Gut Fungal Microbiota with Environmental Factors and Childhood Allergic Outcomes
Source: mBio. 2021 Jun 1;12(3):e03396-20. doi: 10.1128/mBio.03396-20 (PMC8263004; doi:10.1128/mBio.03396-20)

A

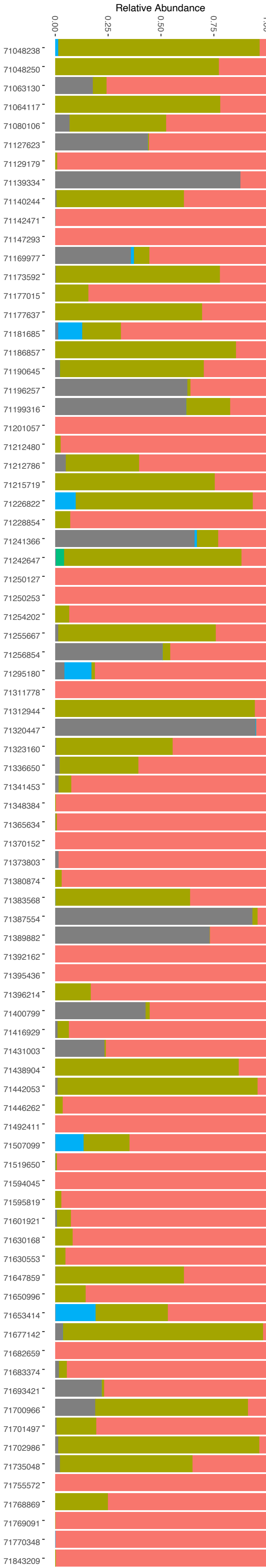

B

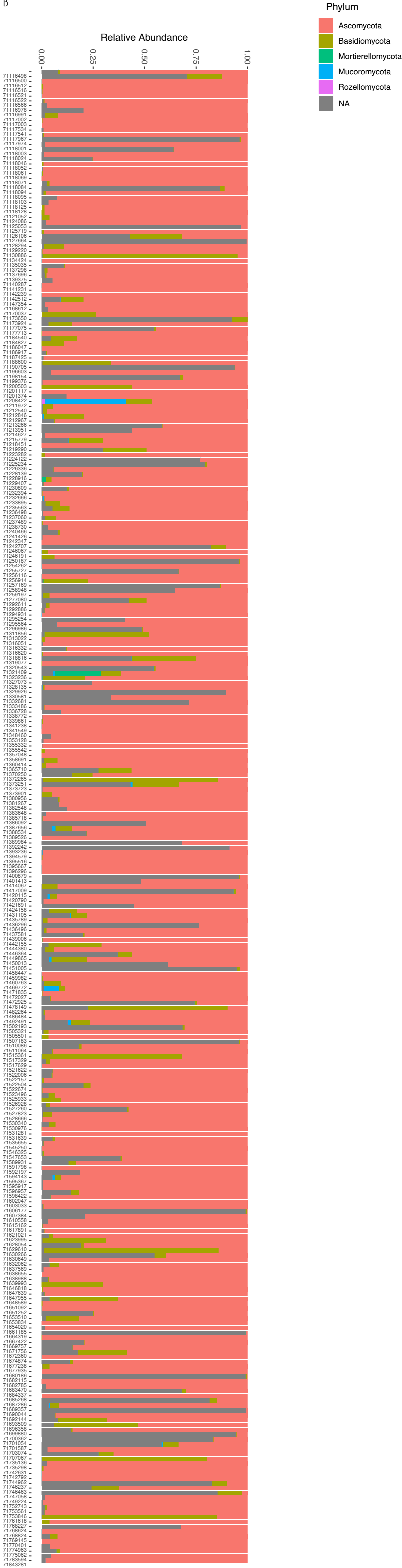

Supplement: FIG S1 [file mbio.03396-20-sf001.pdf]

A

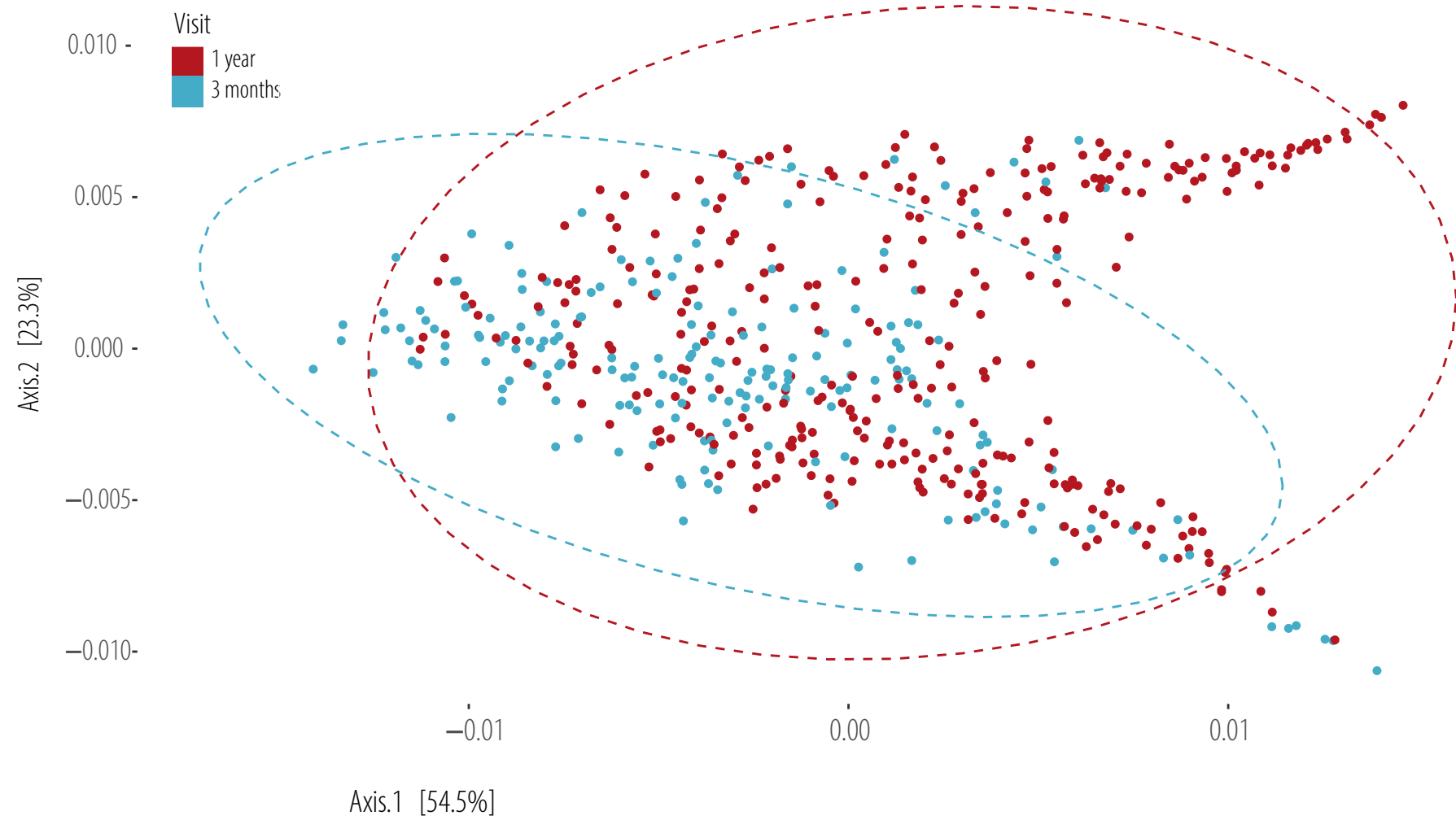

B

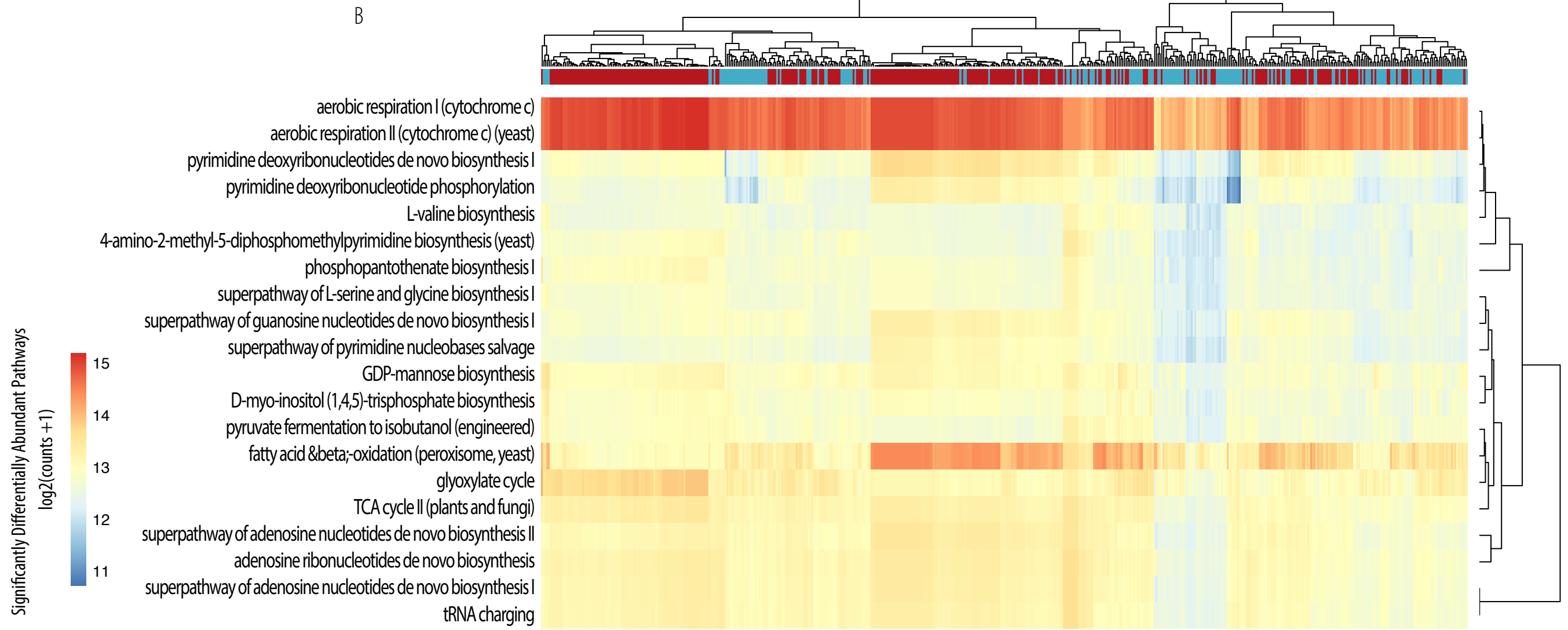

C

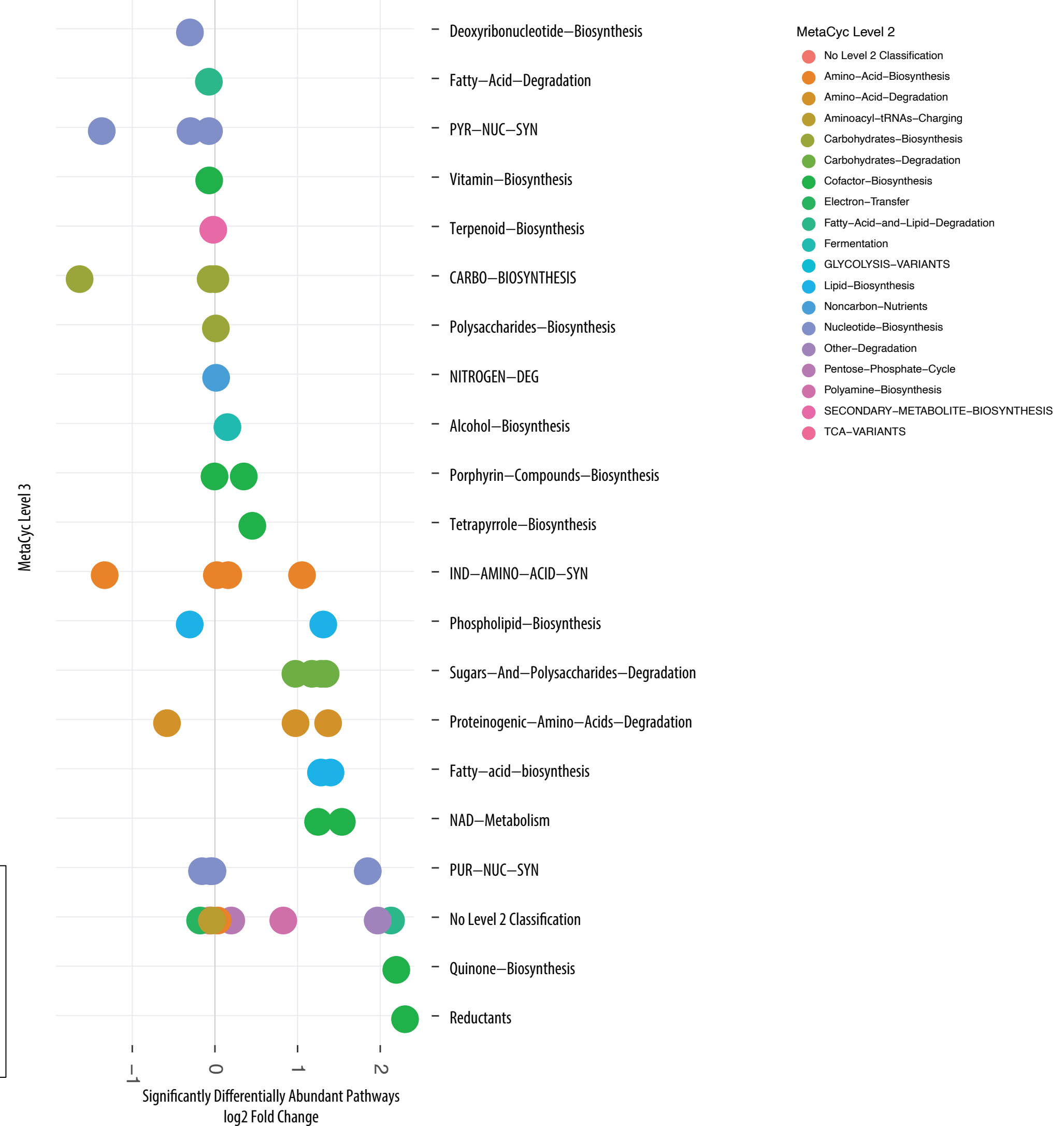

Supplement: FIG S2 [file mbio.03396-20-sf002.pdf]

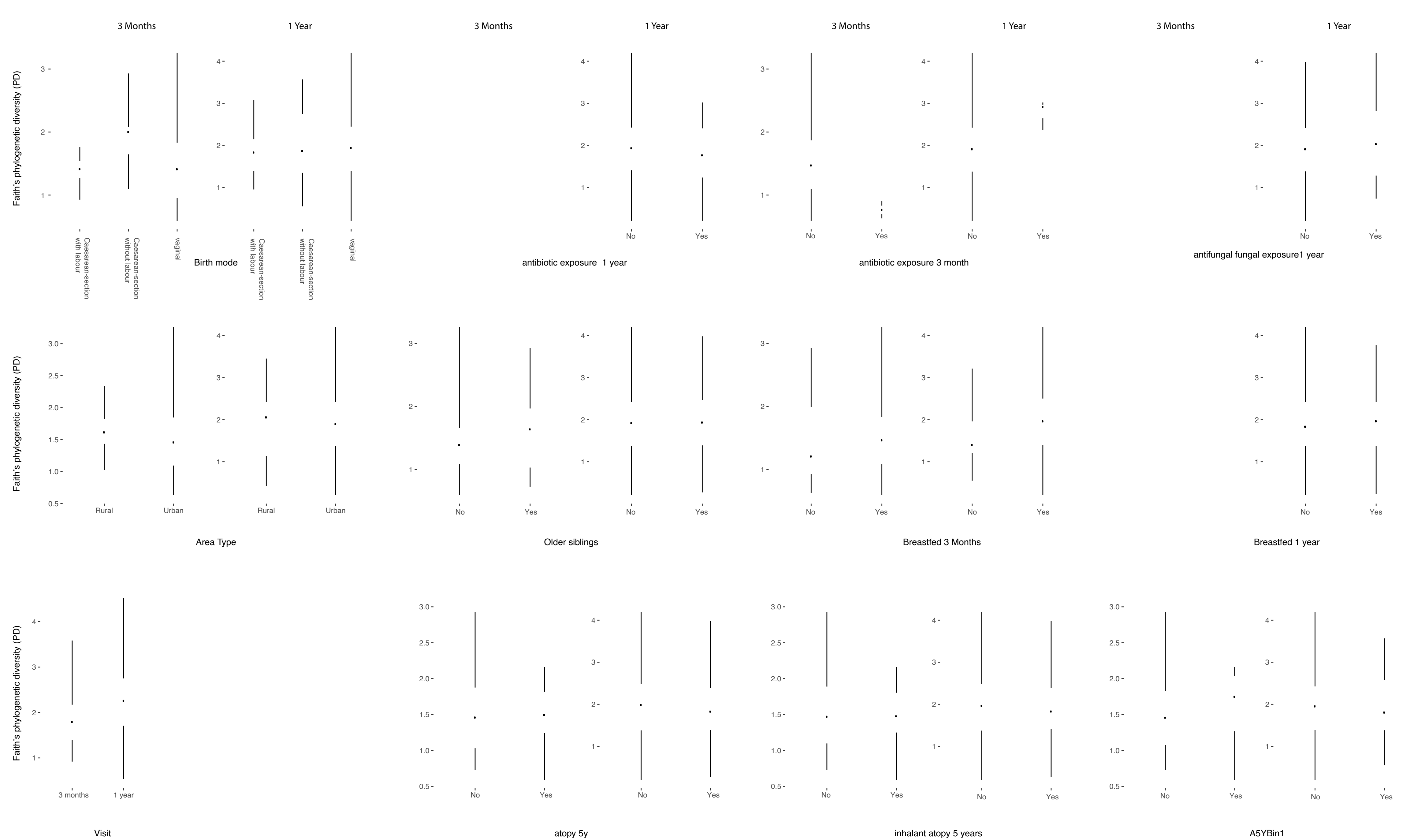

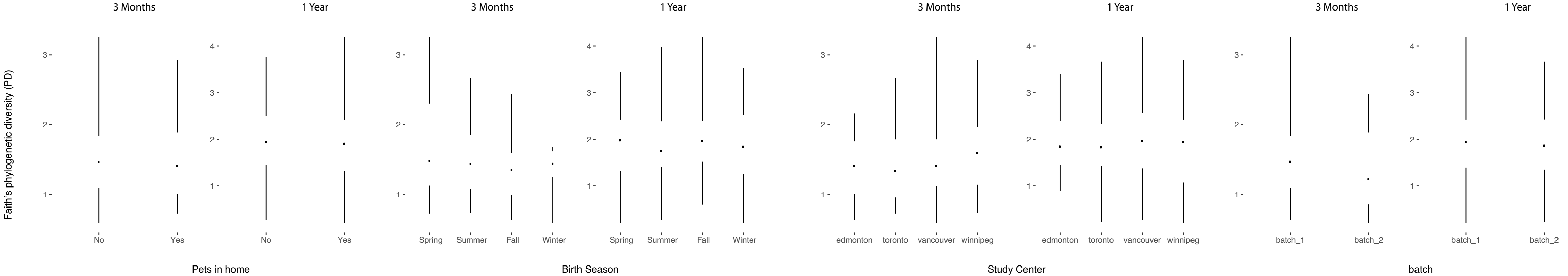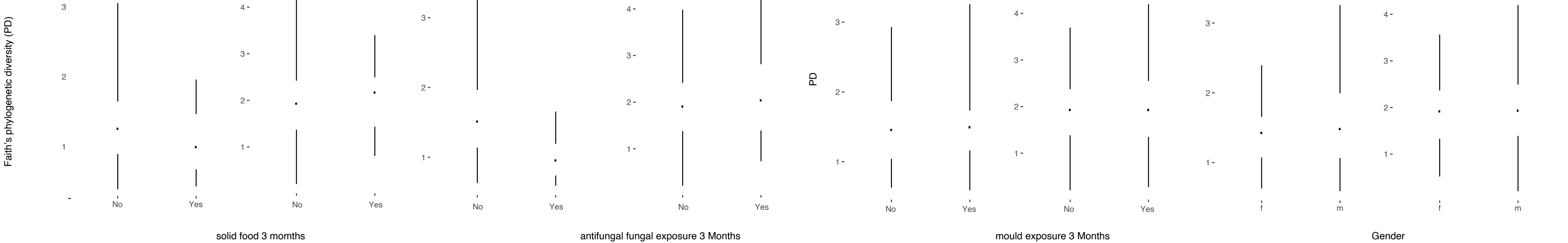

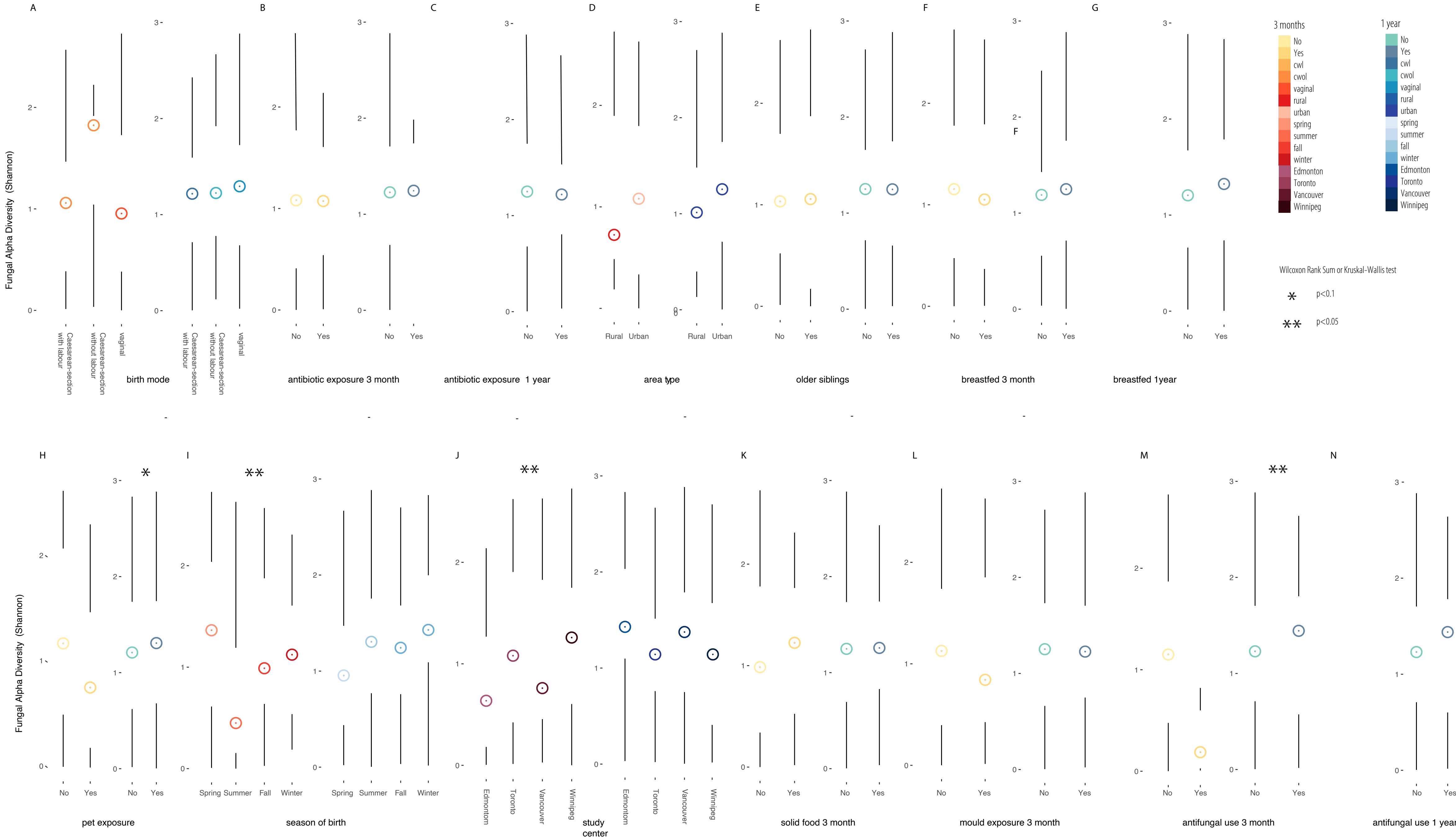

Supplement: FIG S3 [file mbio.03396-20-sf003.pdf]

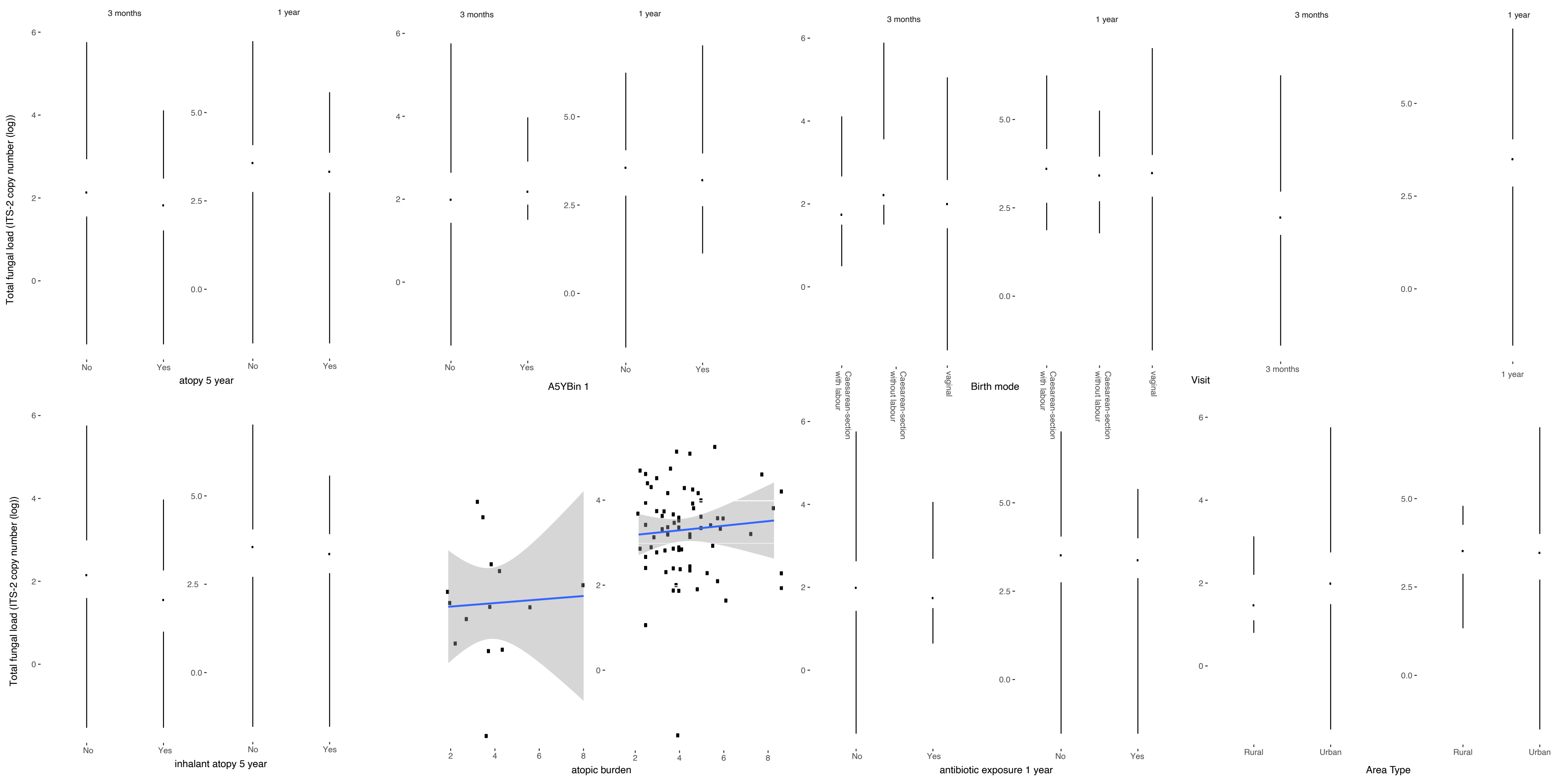

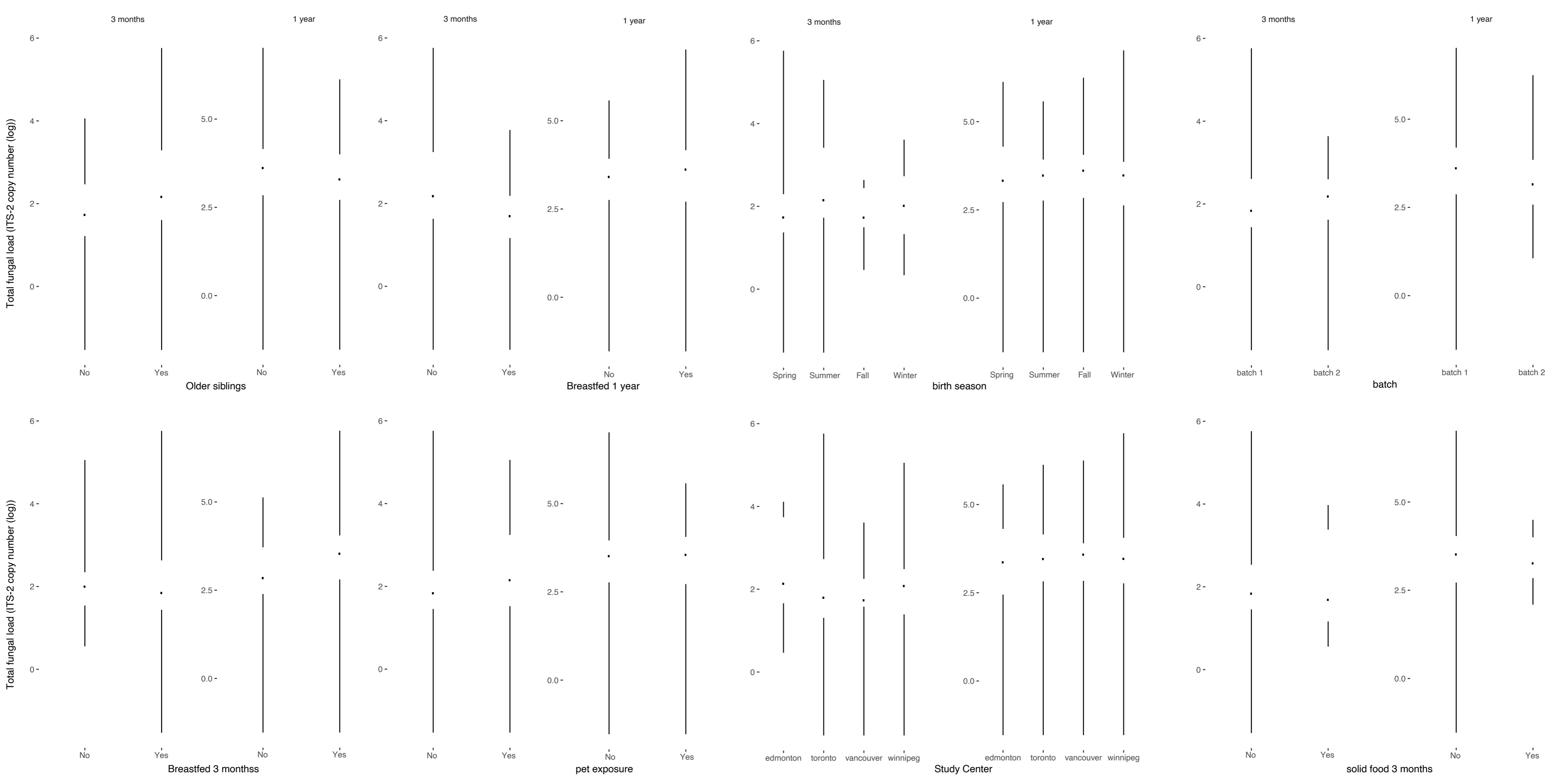

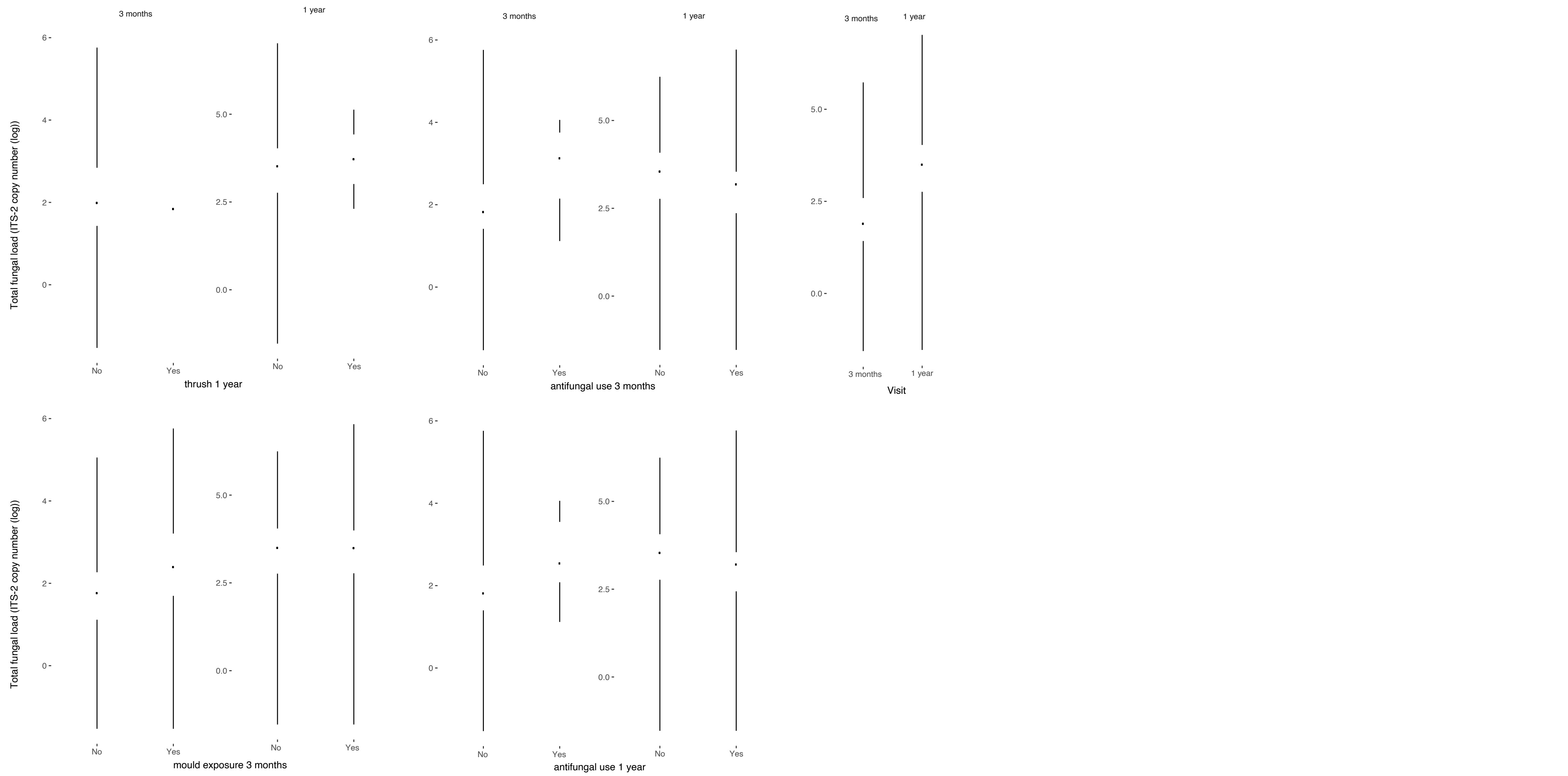

Supplement: FIG S4 [file mbio.03396-20-sf004.pdf]

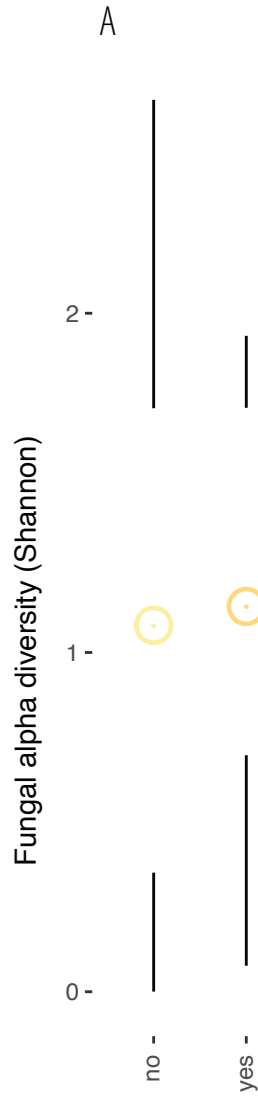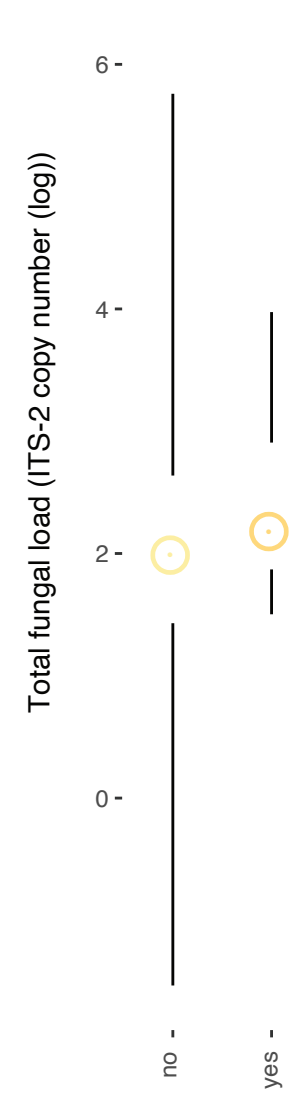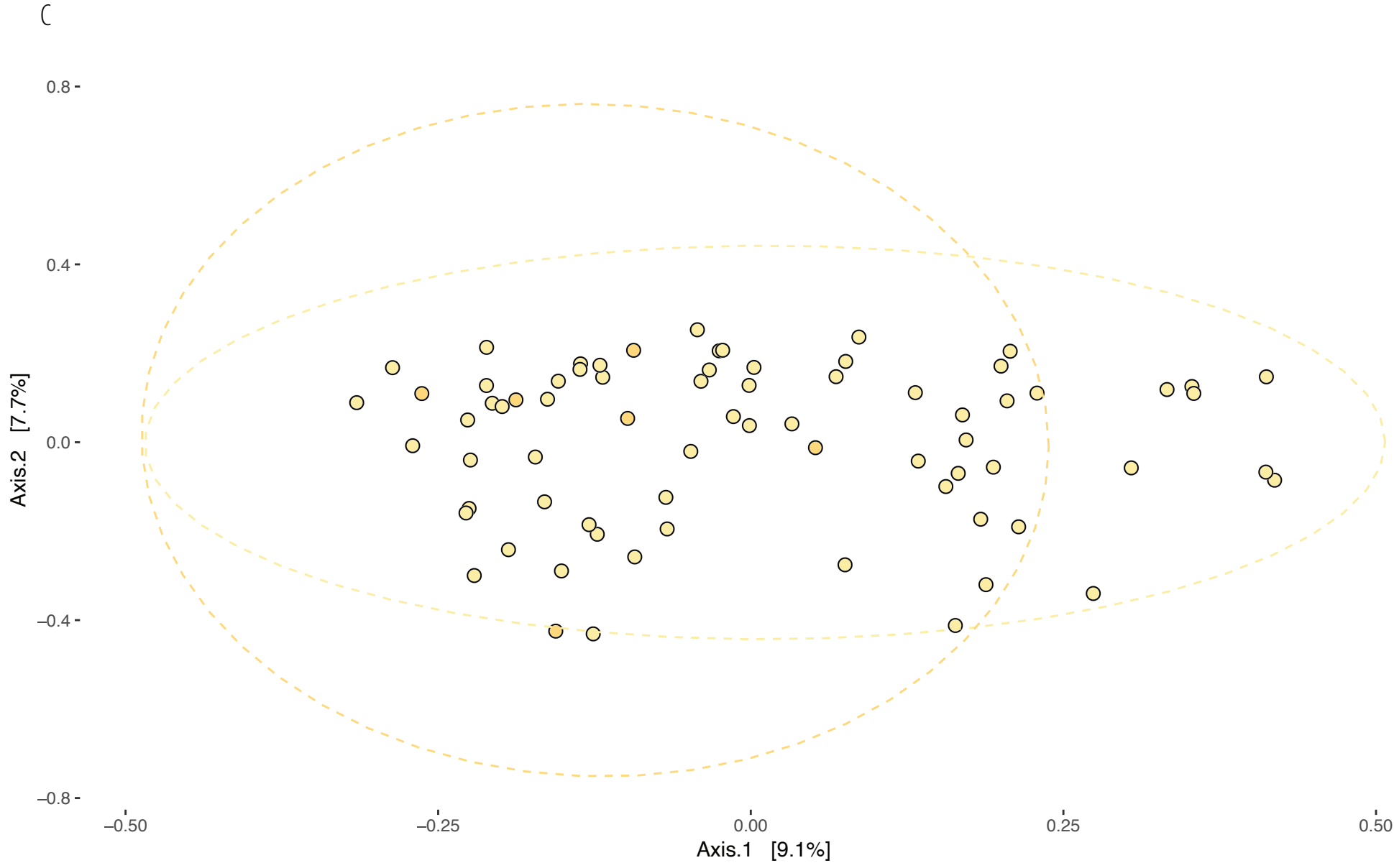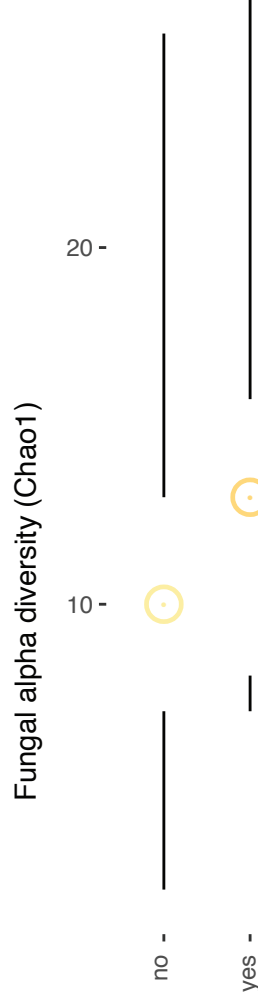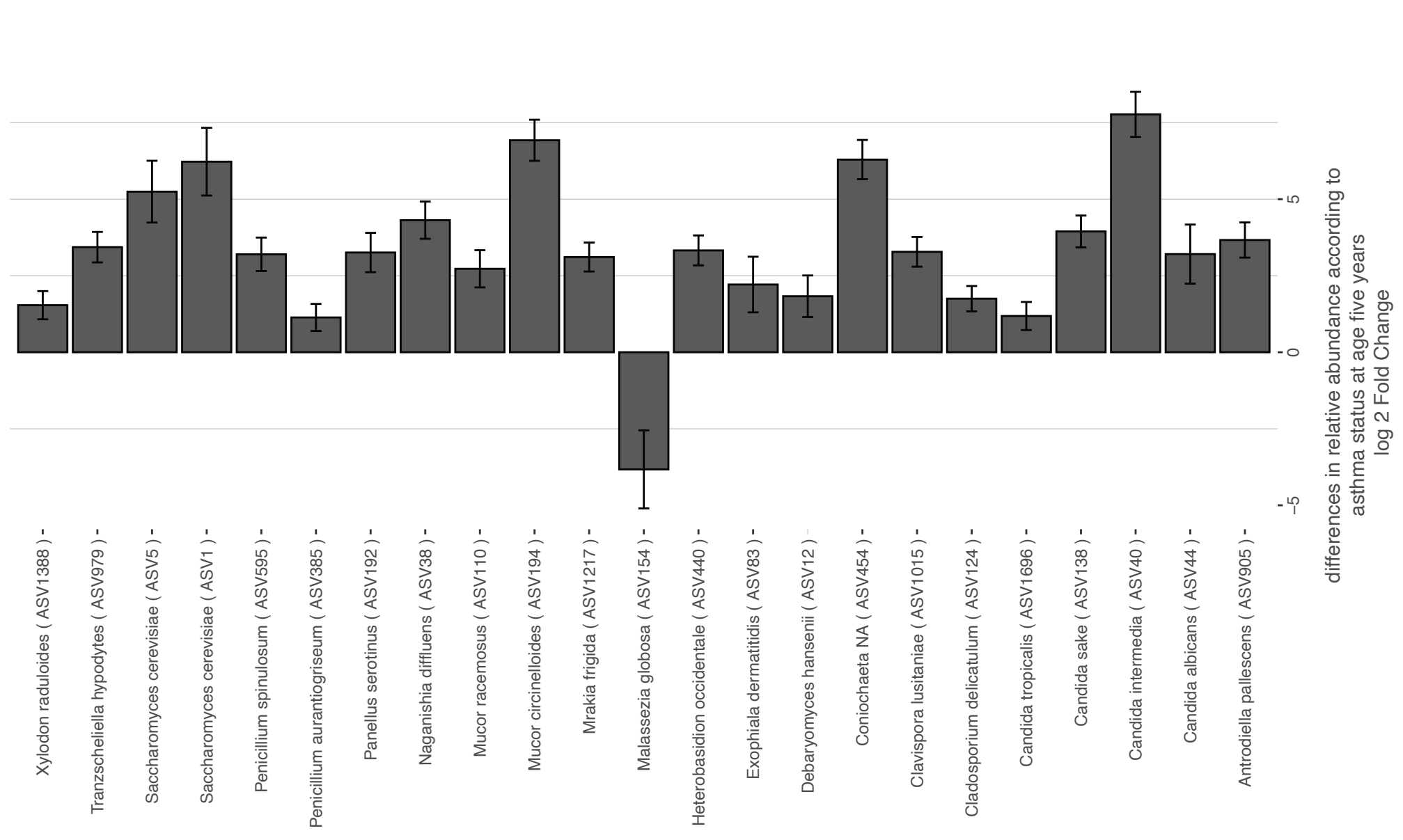

Inhalant atopy at 5 years

No  
Yes

Supplement: FIG S5 [file mbio.03396-20-sf005.pdf]

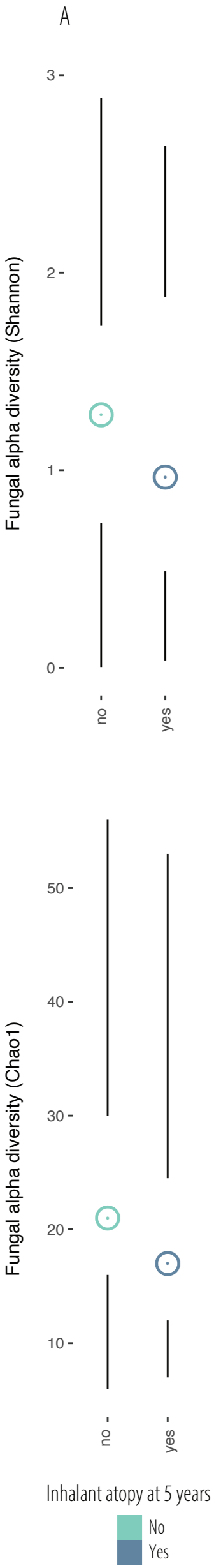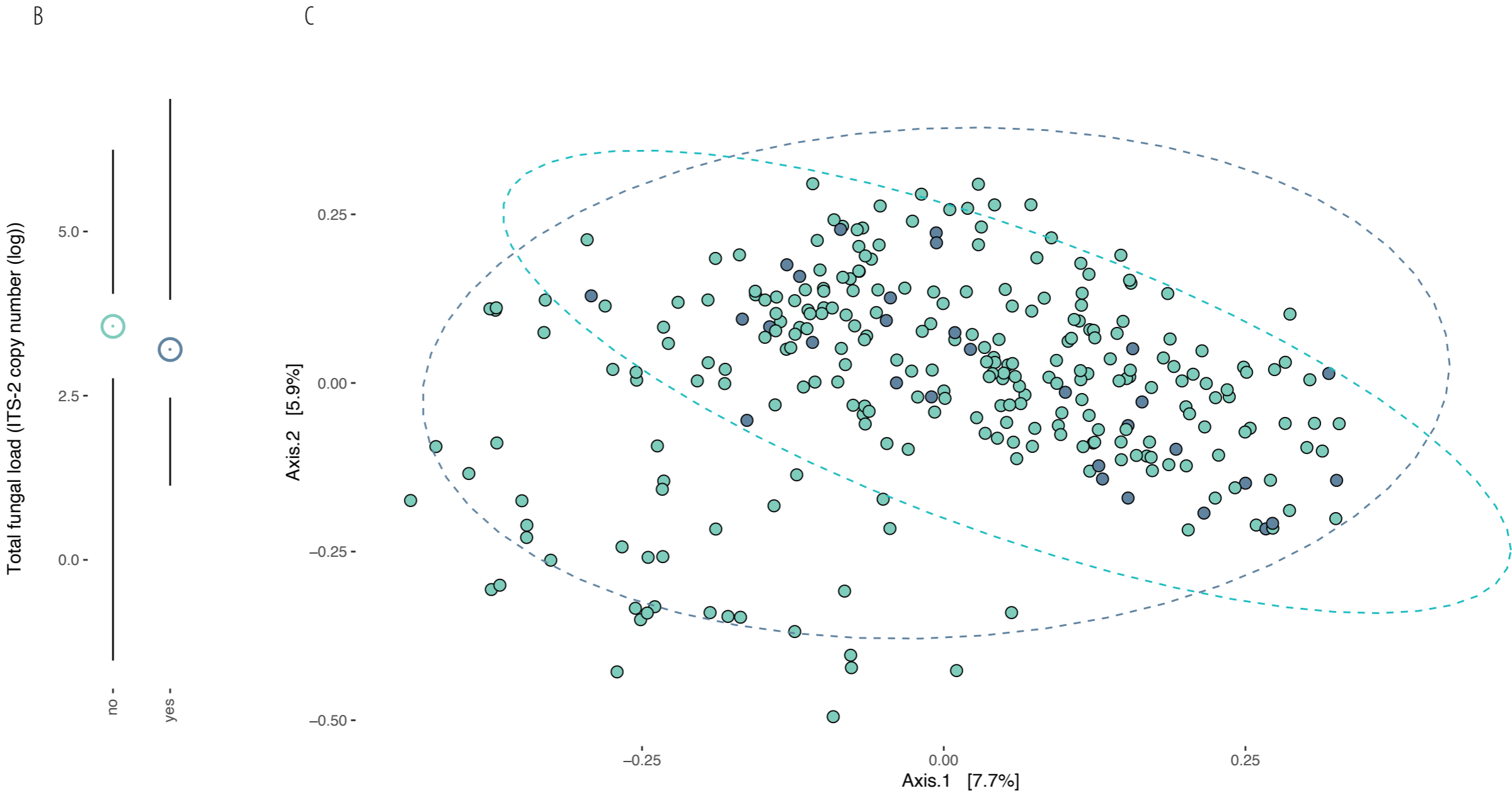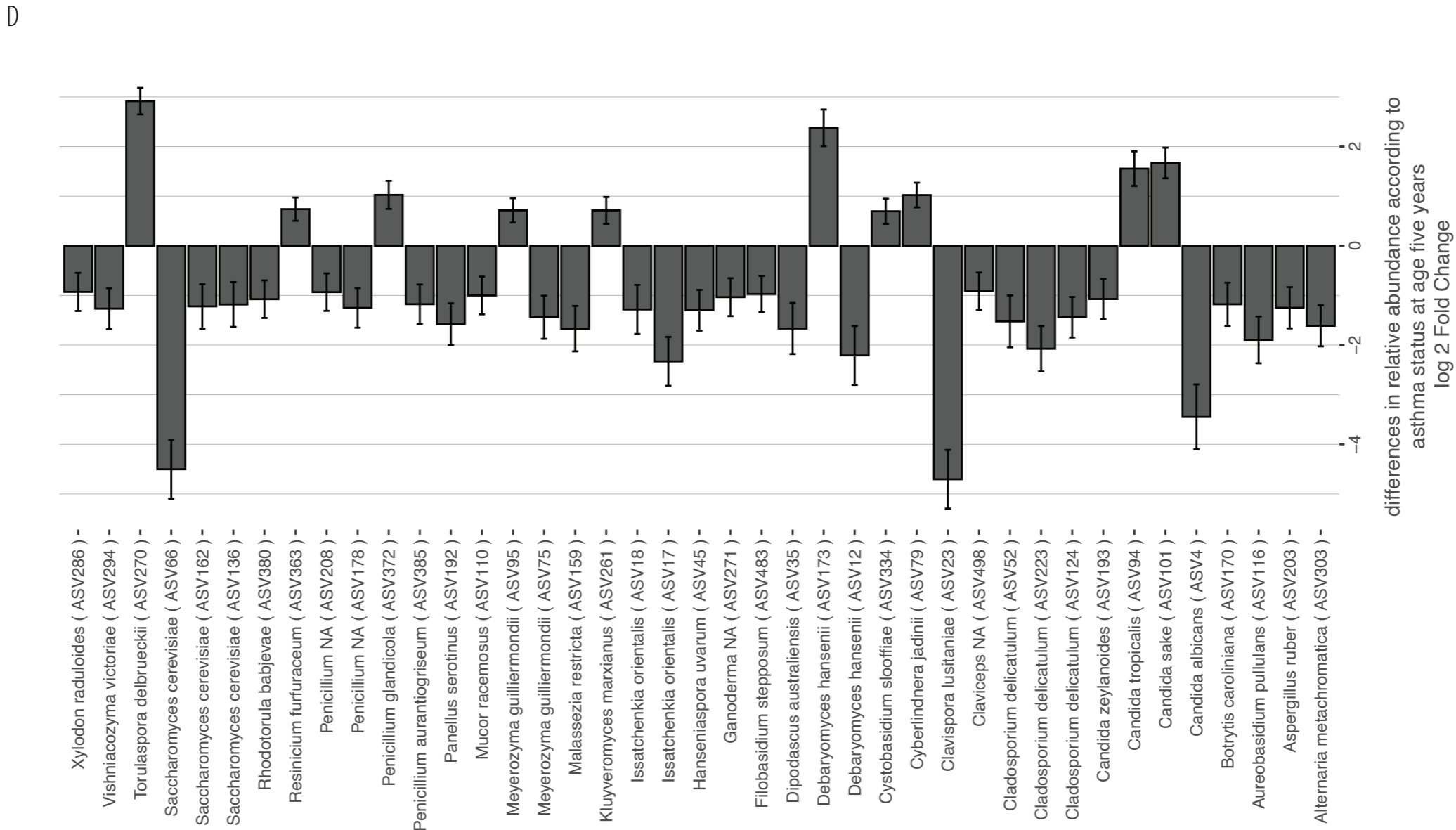

Supplement: FIG S6 [file mbio.03396-20-sf006.pdf]
