# Supplementary material for: Composition and Associations of the Infant Gut Fungal Microbiota with Environmental Factors and Childhood Allergic Outcomes
Source: mBio. 2021 Jun 1;12(3):e03396-20. doi: 10.1128/mBio.03396-20 (PMC8263004; doi:10.1128/mBio.03396-20)
Supplement: TEXT S1 [file mbio.03396-20-t0001.pdf]

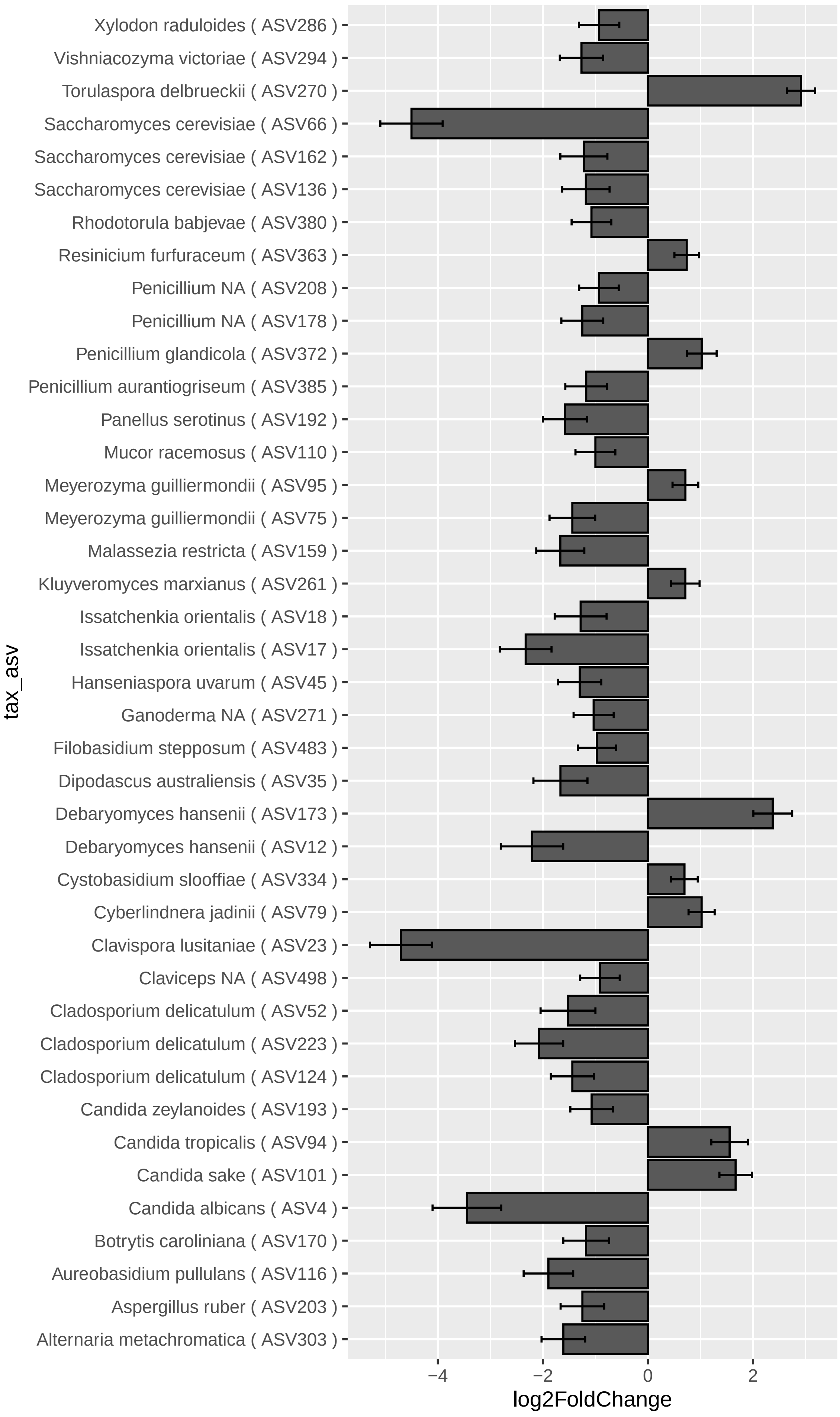
Xylodon raduloides (ASV286)
Vishniacozyma victoriae (ASV294)
Torulaspora delbrueckii (ASV270)
Saccharomyces cerevisiae (ASV66)
Saccharomyces cerevisiae (ASV162)
Saccharomyces cerevisiae (ASV136)
Rhodotorula babjevae (ASV380)
Resinicium furfuraceum (ASV363)
Penicillium NA (ASV208)
Penicillium NA (ASV178)
Penicillium glandicola (ASV372)
Penicillium aurantiogriseum (ASV385)
Panellus serotinus (ASV192)
Mucor racemosus (ASV110)
Meyerozyma guilliermondii (ASV95)
Meyerozyma guilliermondii (ASV75)
Malassezia restricta (ASV159)
Kluyveromyces marxianus (ASV261)
Issatchenkia orientalis (ASV18)
Issatchenkia orientalis (ASV17)
Hanseniaspora uvarum (ASV45)
Ganoderma NA (ASV271)
Filobasidium stepposum (ASV483)
Dipodascus australiensis (ASV35)
Debaryomyces hansenii (ASV173)
Debaryomyces hansenii (ASV12)
Cystobasidium slooffiae (ASV334)
Cyberlindnera jadinii (ASV79)
Clavispora lusitaniae (ASV23)
Claviceps NA (ASV498)
Cladosporium delicatulum (ASV52)
Cladosporium delicatulum (ASV223)
Cladosporium delicatulum (ASV124)
Candida zeylanoides (ASV193)
Candida tropicalis (ASV94)
Candida sake (ASV101)
Candida albicans (ASV4)
Botrytis caroliniana (ASV170)
Aureobasidium pullulans (ASV116)
Aspergillus ruber (ASV203)
Alternaria metachromatica (ASV303)
tax_asv
−4
−2
0
2
log2FoldChange

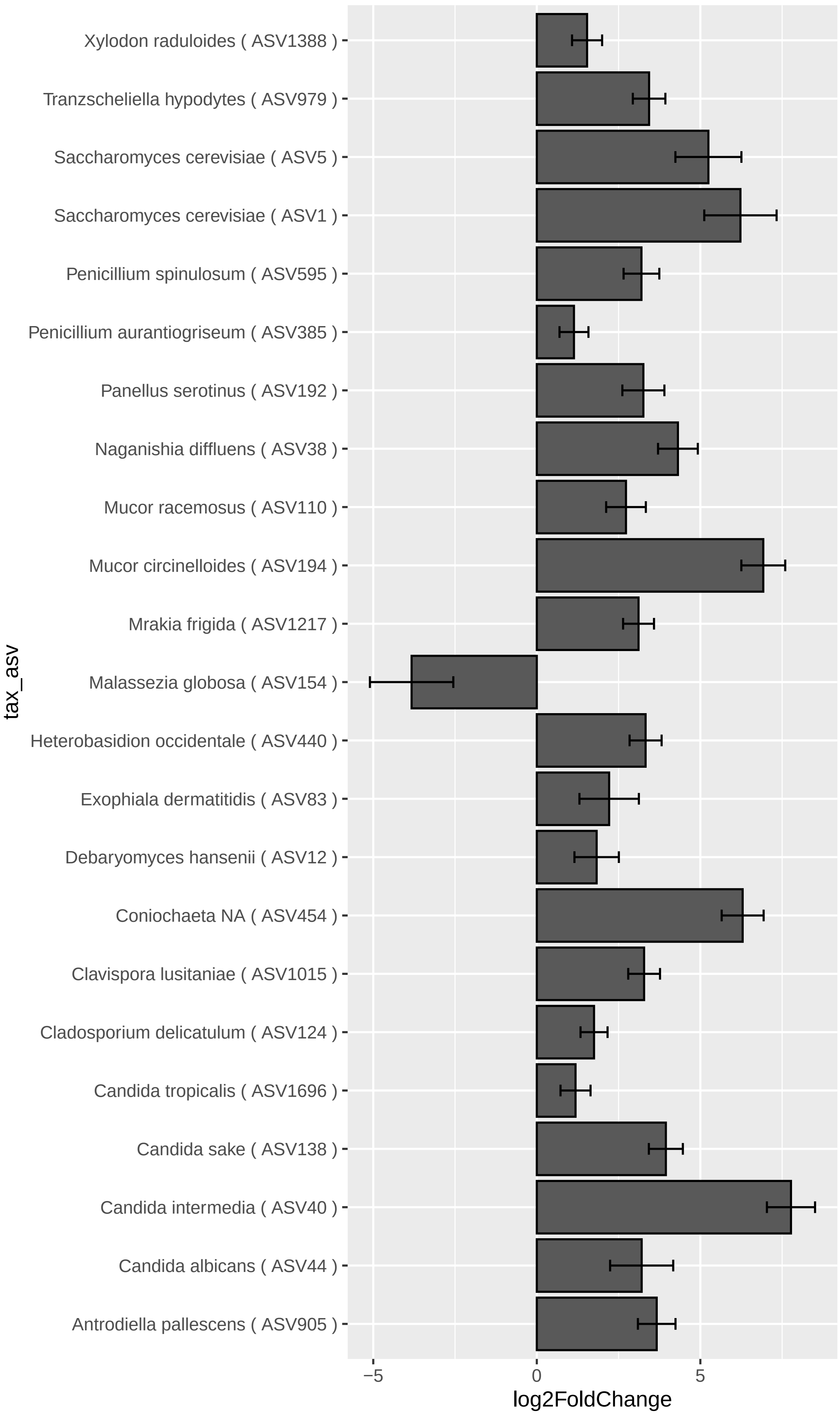
Xylodon raduloides (ASV1388)
Tranzscheliella hypodytes (ASV979)
Saccharomyces cerevisiae (ASV5)
Saccharomyces cerevisiae (ASV1)
Penicillium spinulosum (ASV595)
Penicillium aurantiogriseum (ASV385)
Panellus serotinus (ASV192)
Naganishia diffluens (ASV38)
Mucor racemosus (ASV110)
Mucor circinelloides (ASV194)
Mrakia frigida (ASV1217)
Malassezia globosa (ASV154)
Heterobasidion occidentale (ASV440)
Exophiala dermatitidis (ASV83)
Debaryomyces hansenii (ASV12)
Coniochaeta NA (ASV454)
Clavispora lusitaniae (ASV1015)
Cladosporium delicatulum (ASV124)
Candida tropicalis (ASV1696)
Candida sake (ASV138)
Candida intermedia (ASV40)
Candida albicans (ASV44)
Antrodiella pallescens (ASV905)
tax_asv
−5
0
5
log2FoldChange

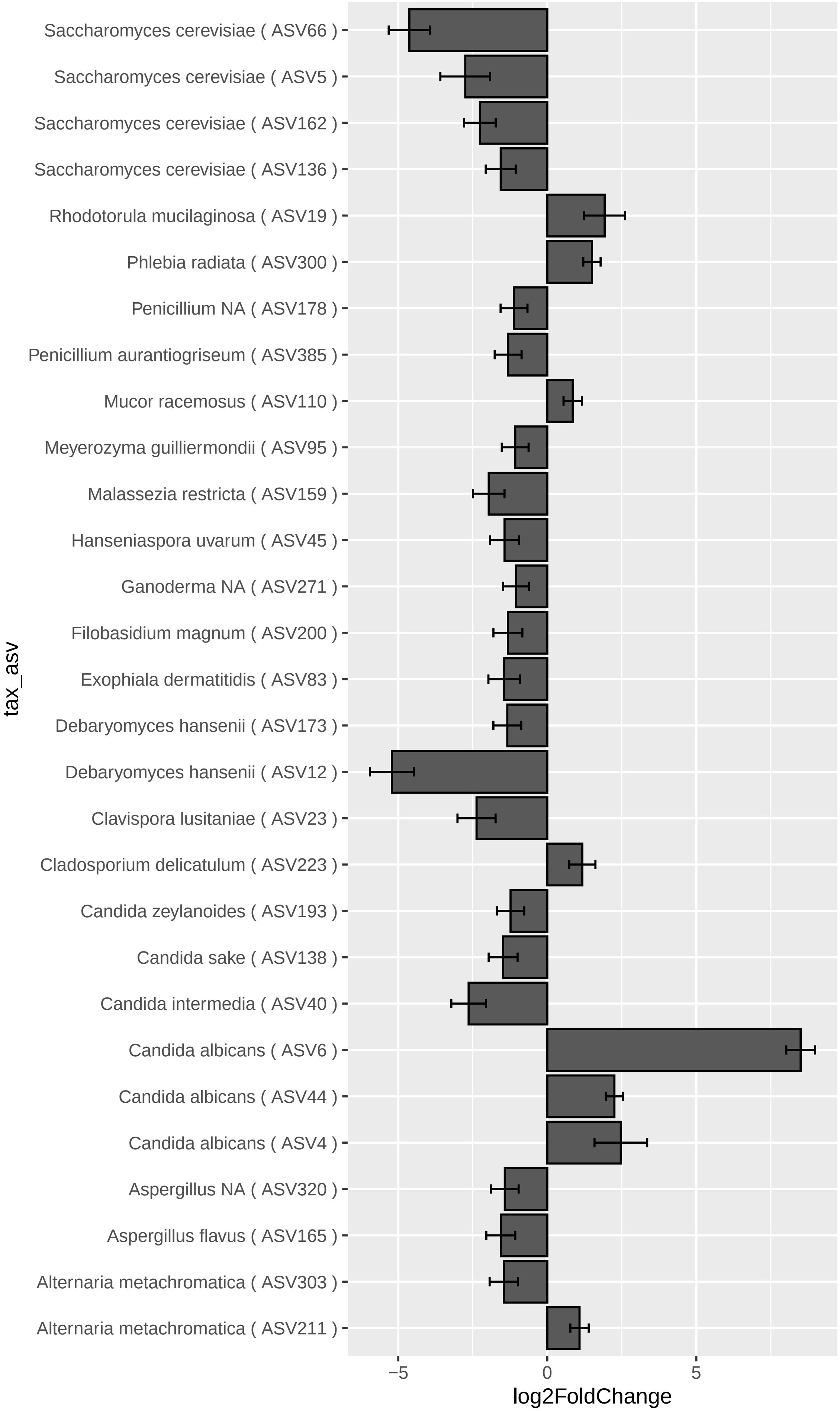
Saccharomyces cerevisiae (ASV66)
Saccharomyces cerevisiae (ASV5)
Saccharomyces cerevisiae (ASV162)
Saccharomyces cerevisiae (ASV136)
Rhodotorula mucilaginosa (ASV19)
Phlebia radiata (ASV300)
Penicillium NA (ASV178)
Penicillium aurantiogriseum (ASV385)
Mucor racemosus (ASV110)
Meyerozyma guilliermondii (ASV95)
Malassezia restricta (ASV159)
Hanseniaspora uvarum (ASV45)
Ganoderma NA (ASV271)
Filobasidium magnum (ASV200)
Exophiala dermatitidis (ASV83)
Debaryomyces hansenii (ASV173)
Debaryomyces hansenii (ASV12)
Clavispora lusitaniae (ASV23)
Cladosporium delicatulum (ASV223)
Candida zeylanoides (ASV193)
Candida sake (ASV138)
Candida intermedia (ASV40)
Candida albicans (ASV6)
Candida albicans (ASV44)
Candida albicans (ASV4)
Aspergillus NA (ASV320)
Aspergillus flavus (ASV165)
Alternaria metachromatica (ASV303)
Alternaria metachromatica (ASV211)
tax_asv
−5
0
5
log2FoldChange

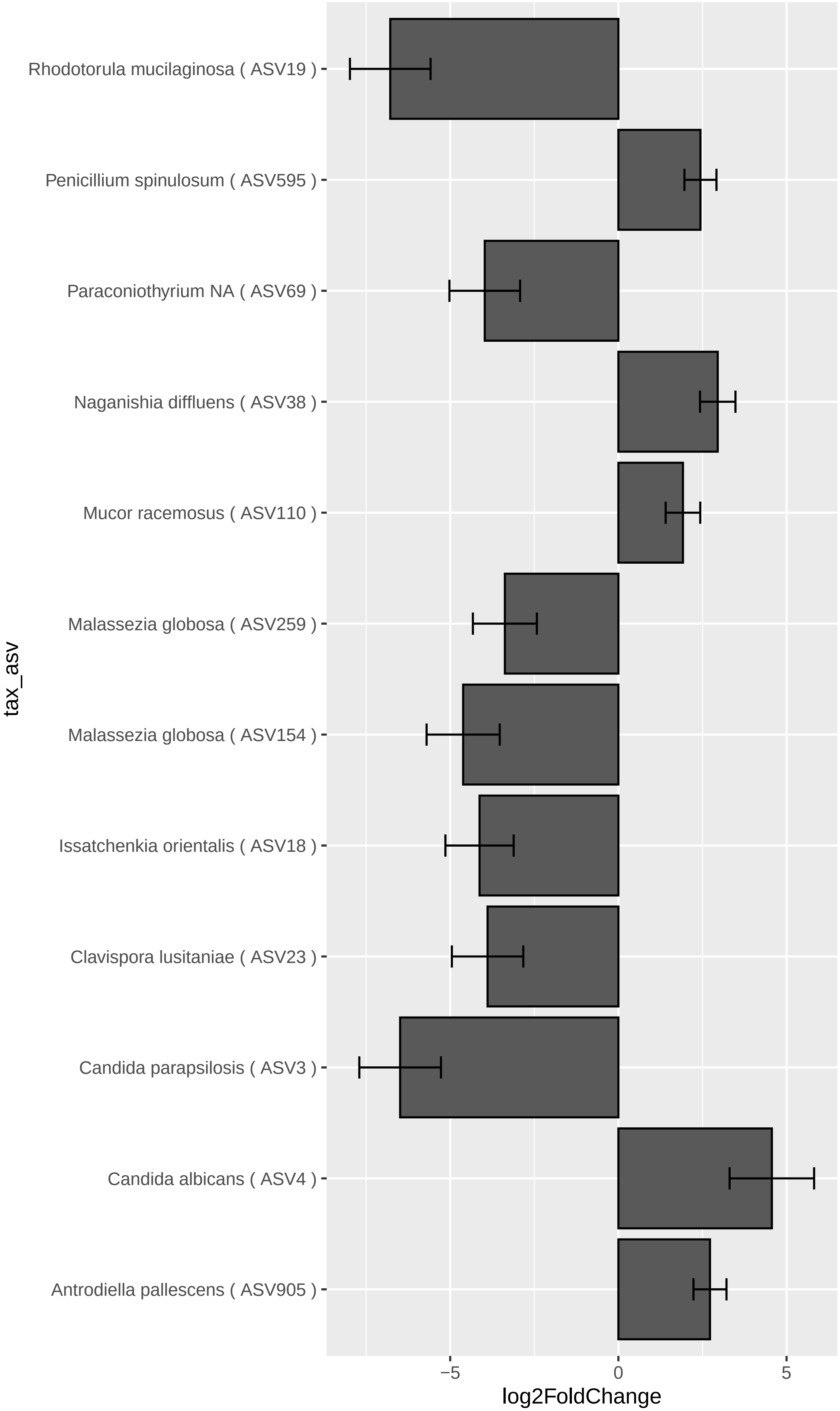

Rhodotorula mucilaginosa (ASV19)
Penicillium spinulosum (ASV595)
Paraconiothyrium NA (ASV69)
Naganishia diffluens (ASV38)
Mucor racemosus (ASV110)
Malassezia globosa (ASV259)
Malassezia globosa (ASV154)
Issatchenkia orientalis (ASV18)
Clavispora lusitaniae (ASV23)
Candida parapsilosis (ASV3)
Candida albicans (ASV4)
Antrodiella pallescens (ASV905)
tax_asv
−5
0
5
log2FoldChange

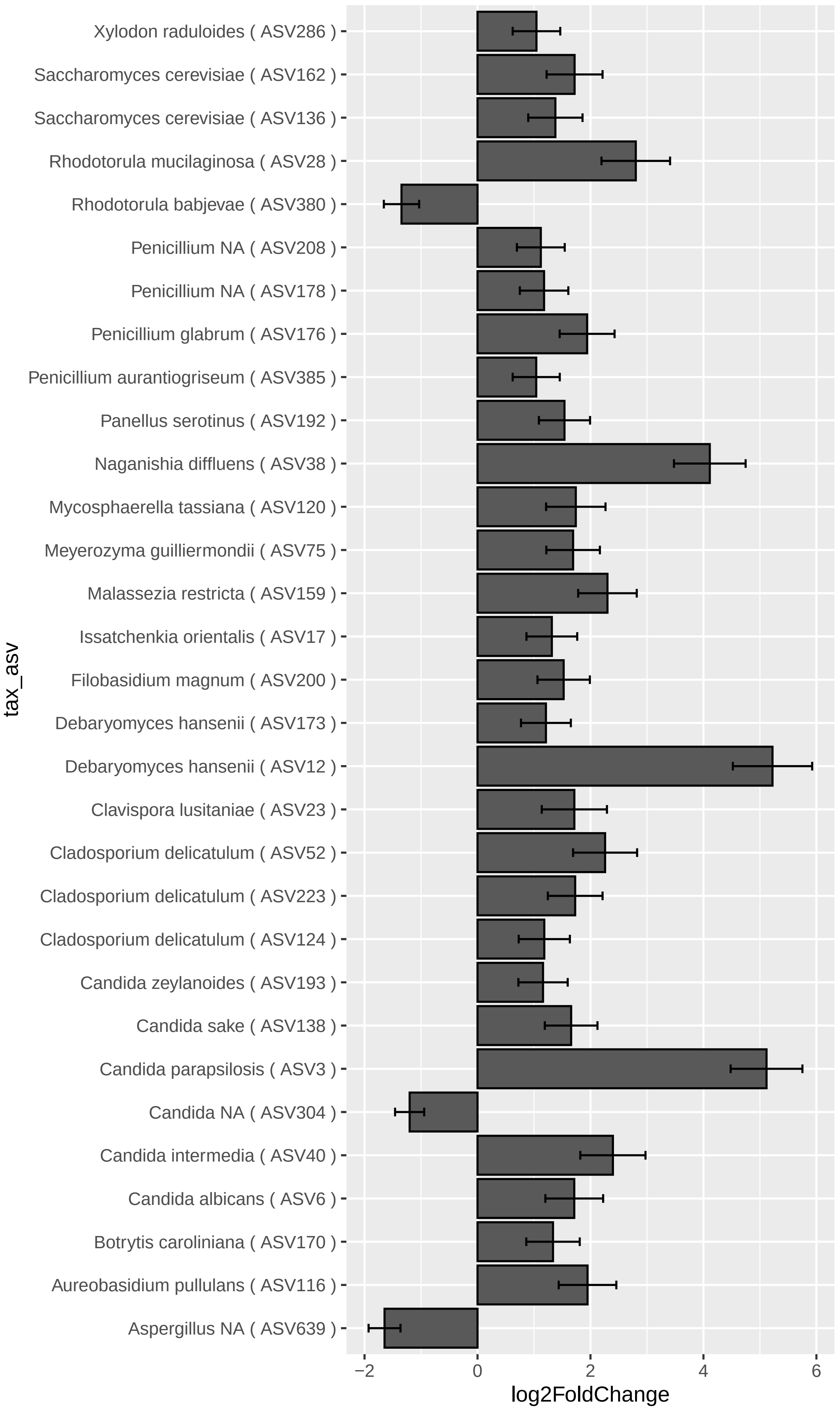

Xylodon raduloides (ASV286)
Saccharomyces cerevisiae (ASV162)
Saccharomyces cerevisiae (ASV136)
Rhodotorula mucilaginosa (ASV28)
Rhodotorula babjevae (ASV380)
Penicillium NA (ASV208)
Penicillium NA (ASV178)
Penicillium glabrum (ASV176)
Penicillium aurantiogriseum (ASV385)
Panellus serotinus (ASV192)
Naganishia diffluens (ASV38)
Mycosphaerella tassiana (ASV120)
Meyerozyma guilliermondii (ASV75)
Malassezia restricta (ASV159)
Issatchenkia orientalis (ASV17)
Filobasidium magnum (ASV200)
Debaryomyces hansenii (ASV173)
Debaryomyces hansenii (ASV12)
Clavispora lusitaniae (ASV23)
Cladosporium delicatulum (ASV52)
Cladosporium delicatulum (ASV223)
Cladosporium delicatulum (ASV124)
Candida zeylanoides (ASV193)
Candida sake (ASV138)
Candida parapsilosis (ASV3)
Candida NA (ASV304)
Candida intermedia (ASV40)
Candida albicans (ASV6)
Botrytis caroliniana (ASV170)
Aureobasidium pullulans (ASV116)
Aspergillus NA (ASV639)
tax_asv
−2
0
2
4
6
log2FoldChange

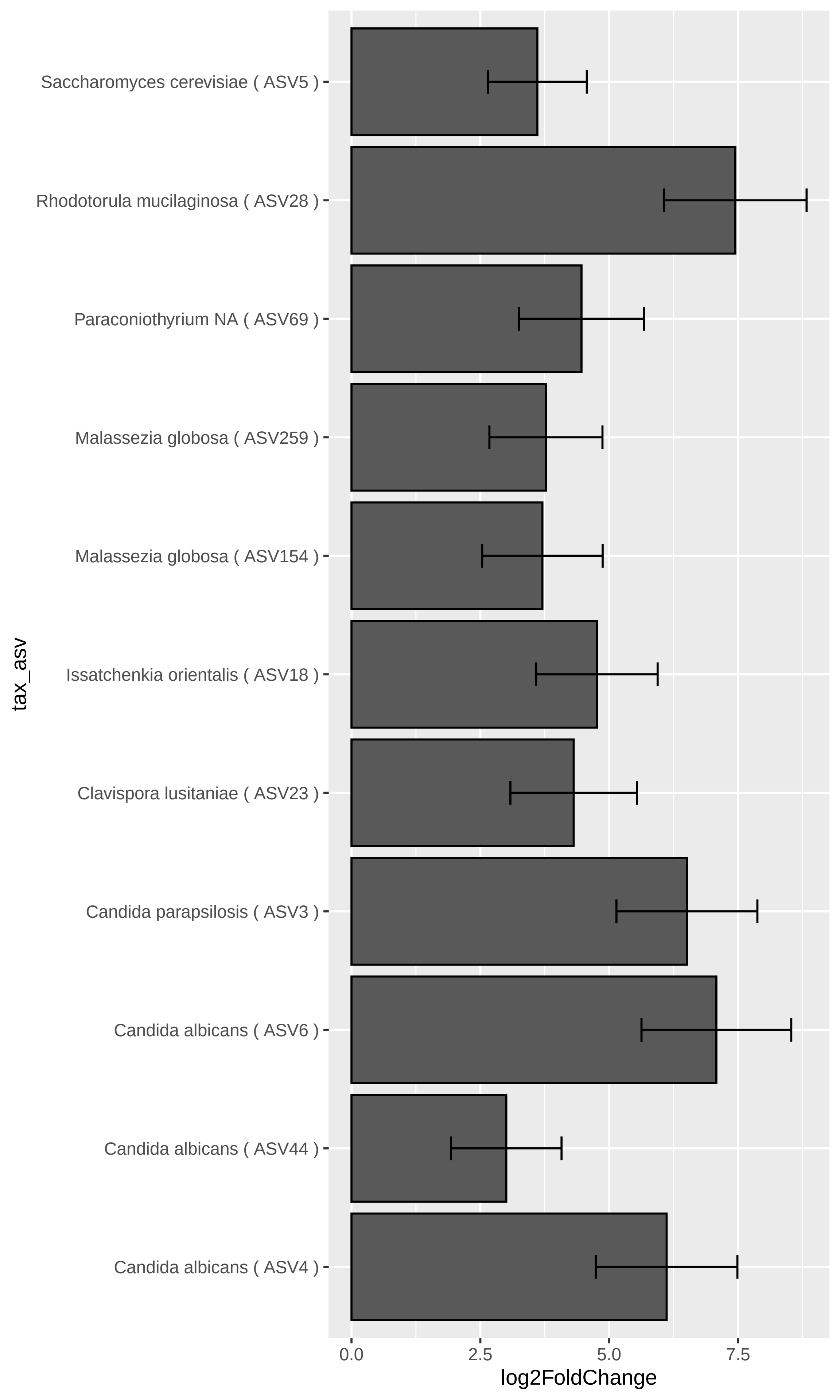
Saccharomyces cerevisiae (ASV5)
Rhodotorula mucilaginosa (ASV28)
Paraconiothyrium NA (ASV69)
Malassezia globosa (ASV259)
Malassezia globosa (ASV154)
Issatchenkia orientalis (ASV18)
Clavispora lusitaniae (ASV23)
Candida parapsilosis (ASV3)
Candida albicans (ASV6)
Candida albicans (ASV44)
Candida albicans (ASV4)
tax_asv
0.0
2.5
5.0
7.5
log2FoldChange

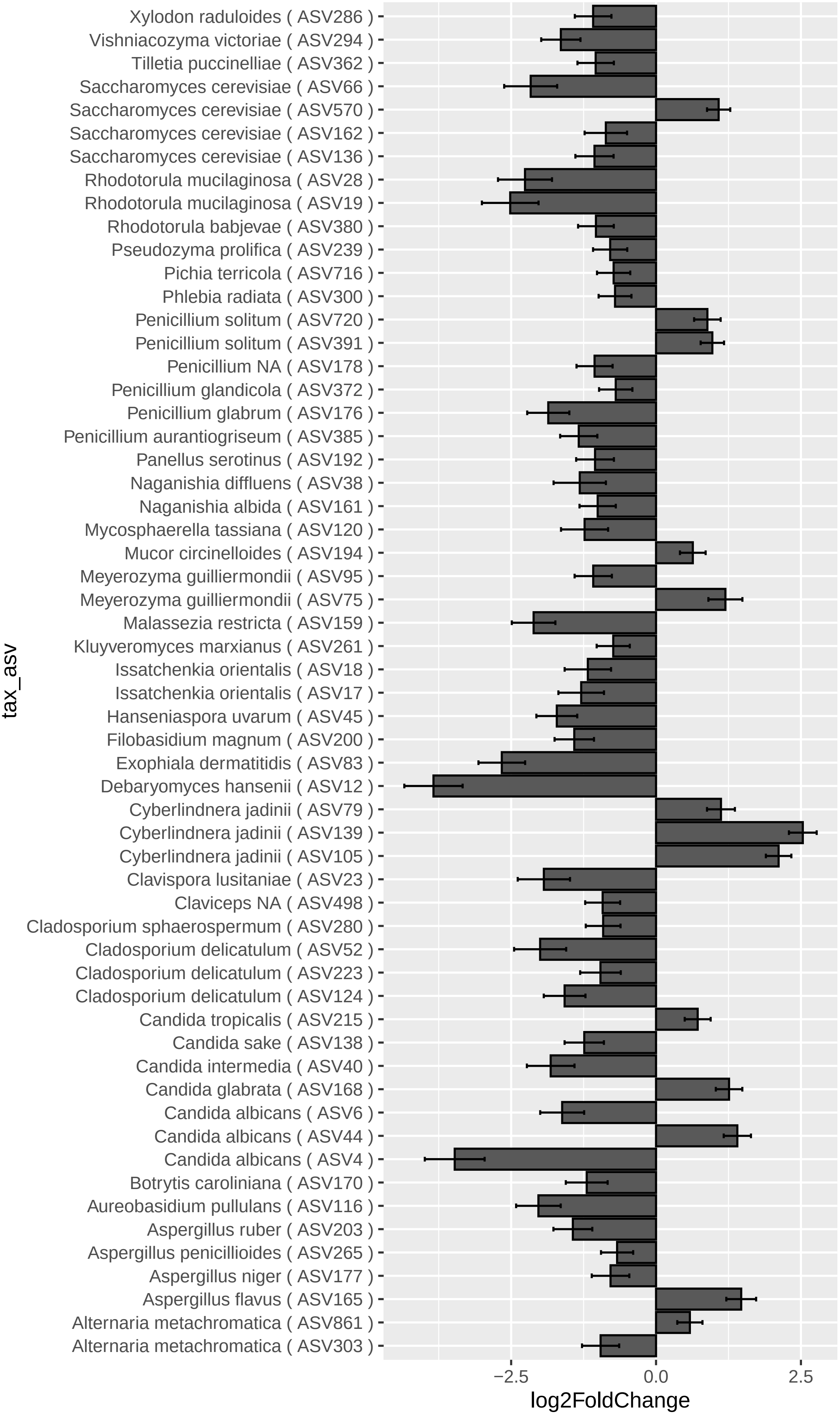
Xylodon raduloides (ASV286)
Vishniacozyma victoriae (ASV294)
Tilletia puccinelliae (ASV362)
Saccharomyces cerevisiae (ASV66)
Saccharomyces cerevisiae (ASV570)
Saccharomyces cerevisiae (ASV162)
Saccharomyces cerevisiae (ASV136)
Rhodotorula mucilaginosa (ASV28)
Rhodotorula mucilaginosa (ASV19)
Rhodotorula babjevae (ASV380)
Pseudozyma prolifica (ASV239)
Pichia terricola (ASV716)
Phlebia radiata (ASV300)
Penicillium solitum (ASV720)
Penicillium solitum (ASV391)
Penicillium NA (ASV178)
Penicillium glandicola (ASV372)
Penicillium glabrum (ASV176)
Penicillium aurantiogriseum (ASV385)
Panellus serotinus (ASV192)
Naganishia diffluens (ASV38)
Naganishia albida (ASV161)
Mycosphaerella tassiana (ASV120)
Mucor circinelloides (ASV194)
Meyerozyma guilliermondii (ASV95)
Meyerozyma guilliermondii (ASV75)
Malassezia restricta (ASV159)
Kluyveromyces marxianus (ASV261)
Issatchenkia orientalis (ASV18)
Issatchenkia orientalis (ASV17)
Hanseniaspora uvarum (ASV45)
Filobasidium magnum (ASV200)
Exophiala dermatitidis (ASV83)
Debaryomyces hansenii (ASV12)
Cyberlindnera jadinii (ASV79)
Cyberlindnera jadinii (ASV139)
Cyberlindnera jadinii (ASV105)
Clavispora lusitaniae (ASV23)
Claviceps NA (ASV498)
Cladosporium sphaerospermum (ASV280)
Cladosporium delicatulum (ASV52)
Cladosporium delicatulum (ASV223)
Cladosporium delicatulum (ASV124)
Candida tropicalis (ASV215)
Candida sake (ASV138)
Candida intermedia (ASV40)
Candida glabrata (ASV168)
Candida albicans (ASV6)
Candida albicans (ASV44)
Candida albicans (ASV4)
Botrytis caroliniana (ASV170)
Aureobasidium pullulans (ASV116)
Aspergillus ruber (ASV203)
Aspergillus penicillioides (ASV265)
Aspergillus niger (ASV177)
Aspergillus flavus (ASV165)
Alternaria metachromatica (ASV861)
Alternaria metachromatica (ASV303)
tax_asv
−2.5
0.0
2.5
log2FoldChange

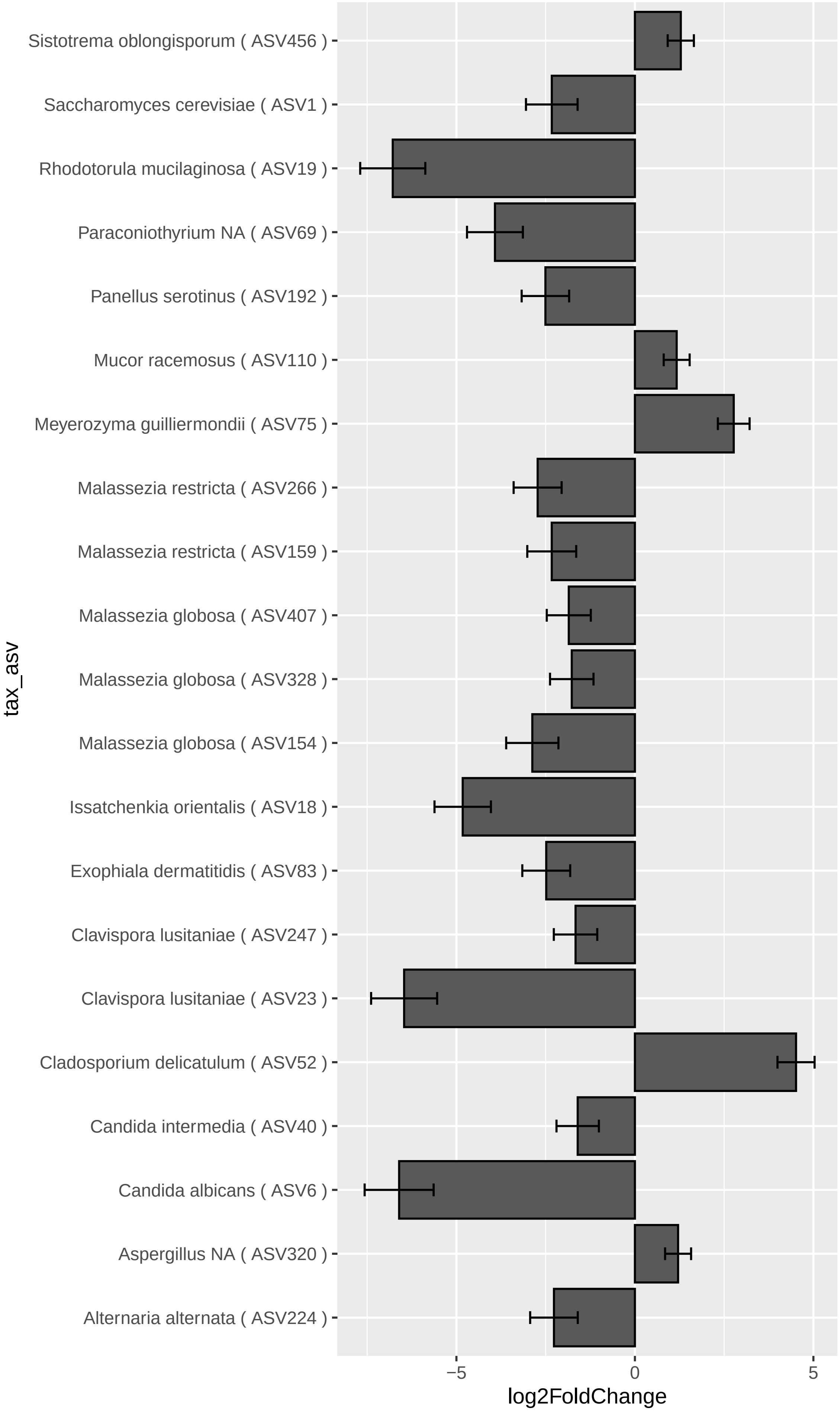

Sistotrema oblongisporum (ASV456)
Saccharomyces cerevisiae (ASV1)
Rhodotorula mucilaginosa (ASV19)
Paraconiothyrium NA (ASV69)
Panellus serotinus (ASV192)
Mucor racemosus (ASV110)
Meyerozyma guilliermondii (ASV75)
Malassezia restricta (ASV266)
Malassezia restricta (ASV159)
Malassezia globosa (ASV407)
Malassezia globosa (ASV328)
Malassezia globosa (ASV154)
Issatchenkia orientalis (ASV18)
Exophiala dermatitidis (ASV83)
Clavispora lusitaniae (ASV247)
Clavispora lusitaniae (ASV23)
Cladosporium delicatulum (ASV52)
Candida intermedia (ASV40)
Candida albicans (ASV6)
Aspergillus NA (ASV320)
Alternaria alternata (ASV224)
tax_asv
−5
0
5
log2FoldChange

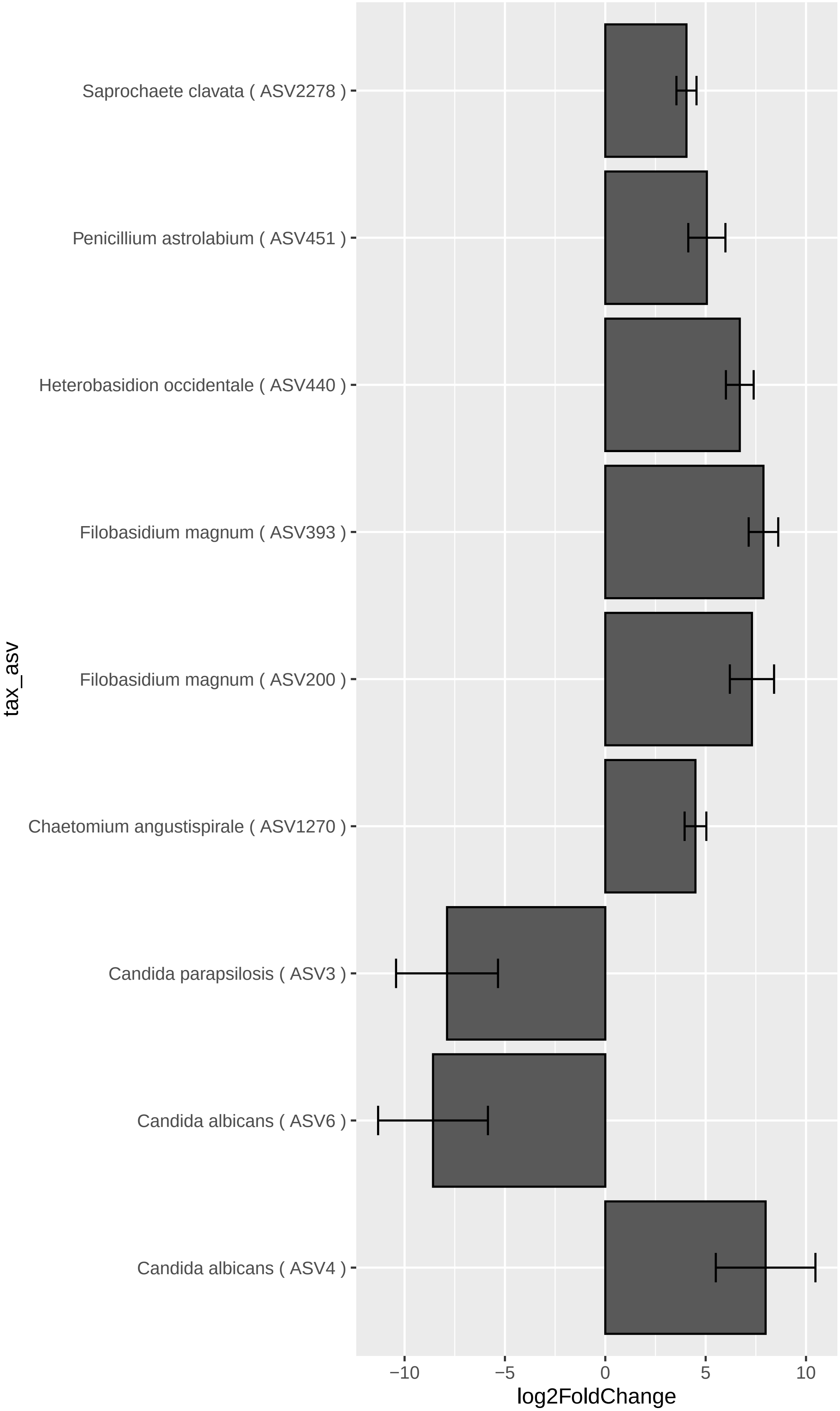
Saprochaete clavata (ASV2278)
Penicillium astrolabium (ASV451)
Heterobasidion occidentale (ASV440)
Filobasidium magnum (ASV393)
Filobasidium magnum (ASV200)
Chaetomium angustispirale (ASV1270)
Candida parapsilosis (ASV3)
Candida albicans (ASV6)
Candida albicans (ASV4)
tax_asv
−10
−5
0
5
10
log2FoldChange

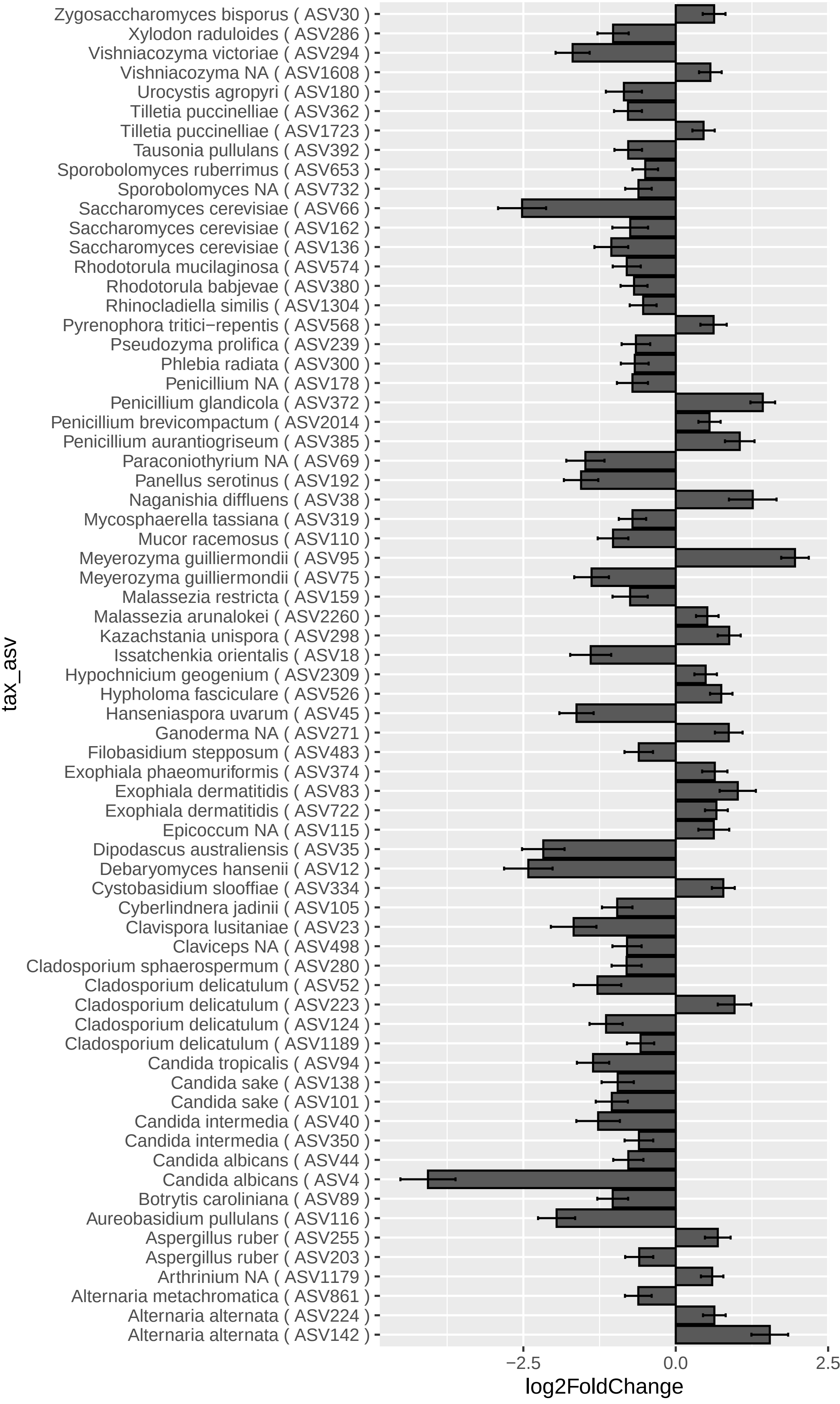

Zygosaccharomyces bisporus (ASV30)
Xylodon raduloides (ASV286)
Vishniacozyma victoriae (ASV294)
Vishniacozyma NA (ASV1608)
Urocystis agropyri (ASV180)
Tilletia puccinelliae (ASV362)
Tilletia puccinelliae (ASV1723)
Tausonia pullulans (ASV392)
Sporobolomyces ruberrimus (ASV653)
Sporobolomyces NA (ASV732)
Saccharomyces cerevisiae (ASV66)
Saccharomyces cerevisiae (ASV162)
Saccharomyces cerevisiae (ASV136)
Rhodotorula mucilaginosa (ASV574)
Rhodotorula babjevae (ASV380)
Rhinocladiella similis (ASV1304)
Pyrenophora tritici−repentis (ASV568)
Pseudozyma prolifica (ASV239)
Phlebia radiata (ASV300)
Penicillium NA (ASV178)
Penicillium glandicola (ASV372)
Penicillium brevicompactum (ASV2014)
Penicillium aurantiogriseum (ASV385)
Paraconiothyrium NA (ASV69)
Panellus serotinus (ASV192)
Naganishia diffluens (ASV38)
Mycosphaerella tassiana (ASV319)
Mucor racemosus (ASV110)
Meyerozyma guilliermondii (ASV95)
Meyerozyma guilliermondii (ASV75)
Malassezia restricta (ASV159)
Malassezia arunalokei (ASV2260)
Kazachstania unispora (ASV298)
Issatchenkia orientalis (ASV18)
Hypochnicium geogenium (ASV2309)
Hypholoma fasciculare (ASV526)
Hanseniaspora uvarum (ASV45)
Ganoderma NA (ASV271)
Filobasidium stepposum (ASV483)
Exophiala phaeomuriformis (ASV374)
Exophiala dermatitidis (ASV83)
Exophiala dermatitidis (ASV722)
Epicoccum NA (ASV115)
Dipodascus australiensis (ASV35)
Debaryomyces hansenii (ASV12)
Cystobasidium slooffiae (ASV334)
Cyberlindnera jadinii (ASV105)
Clavispora lusitaniae (ASV23)
Claviceps NA (ASV498)
Cladosporium sphaerospermum (ASV280)
Cladosporium delicatulum (ASV52)
Cladosporium delicatulum (ASV223)
Cladosporium delicatulum (ASV124)
Cladosporium delicatulum (ASV1189)
Candida tropicalis (ASV94)
Candida sake (ASV138)
Candida sake (ASV101)
Candida intermedia (ASV40)
Candida intermedia (ASV350)
Candida albicans (ASV44)
Candida albicans (ASV4)
Botrytis caroliniana (ASV89)
Aureobasidium pullulans (ASV116)
Aspergillus ruber (ASV255)
Aspergillus ruber (ASV203)
Arthrinium NA (ASV1179)
Alternaria metachromatica (ASV861)
Alternaria alternata (ASV224)
Alternaria alternata (ASV142)
tax_asv
−2.5
0.0
2.5
log2FoldChange

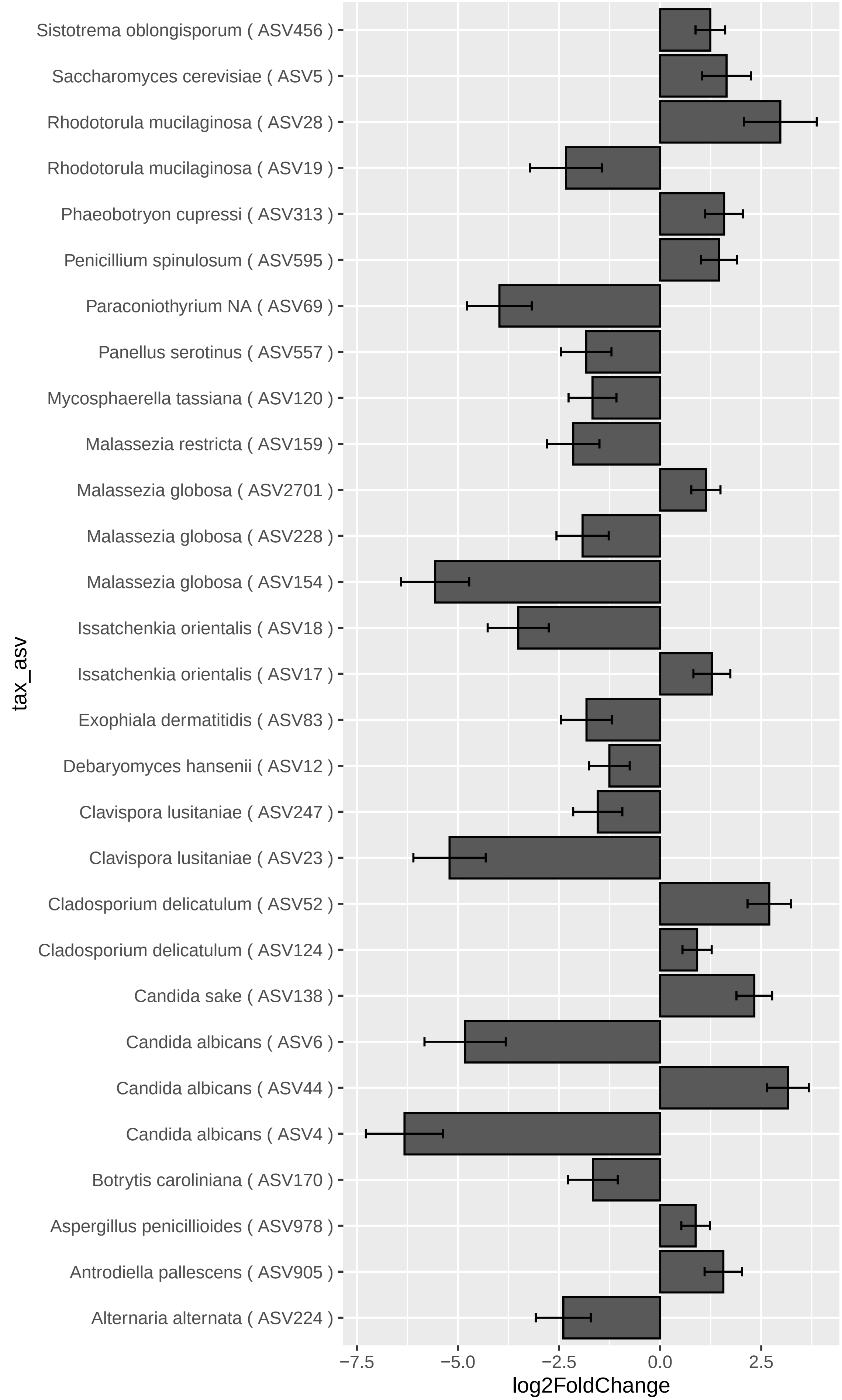
Sistotrema oblongisporum (ASV456)
Saccharomyces cerevisiae (ASV5)
Rhodotorula mucilaginosa (ASV28)
Rhodotorula mucilaginosa (ASV19)
Phaeobotryon cupressi (ASV313)
Penicillium spinulosum (ASV595)
Paraconiothyrium NA (ASV69)
Panellus serotinus (ASV557)
Mycosphaerella tassiana (ASV120)
Malassezia restricta (ASV159)
Malassezia globosa (ASV2701)
Malassezia globosa (ASV228)
Malassezia globosa (ASV154)
Issatchenkia orientalis (ASV18)
Issatchenkia orientalis (ASV17)
Exophiala dermatitidis (ASV83)
Debaryomyces hansenii (ASV12)
Clavispora lusitaniae (ASV247)
Clavispora lusitaniae (ASV23)
Cladosporium delicatulum (ASV52)
Cladosporium delicatulum (ASV124)
Candida sake (ASV138)
Candida albicans (ASV6)
Candida albicans (ASV44)
Candida albicans (ASV4)
Botrytis caroliniana (ASV170)
Aspergillus penicillioides (ASV978)
Antrodiella pallescens (ASV905)
Alternaria alternata (ASV224)
tax_asv
−7.5
−5.0
−2.5
0.0
2.5
log2FoldChange

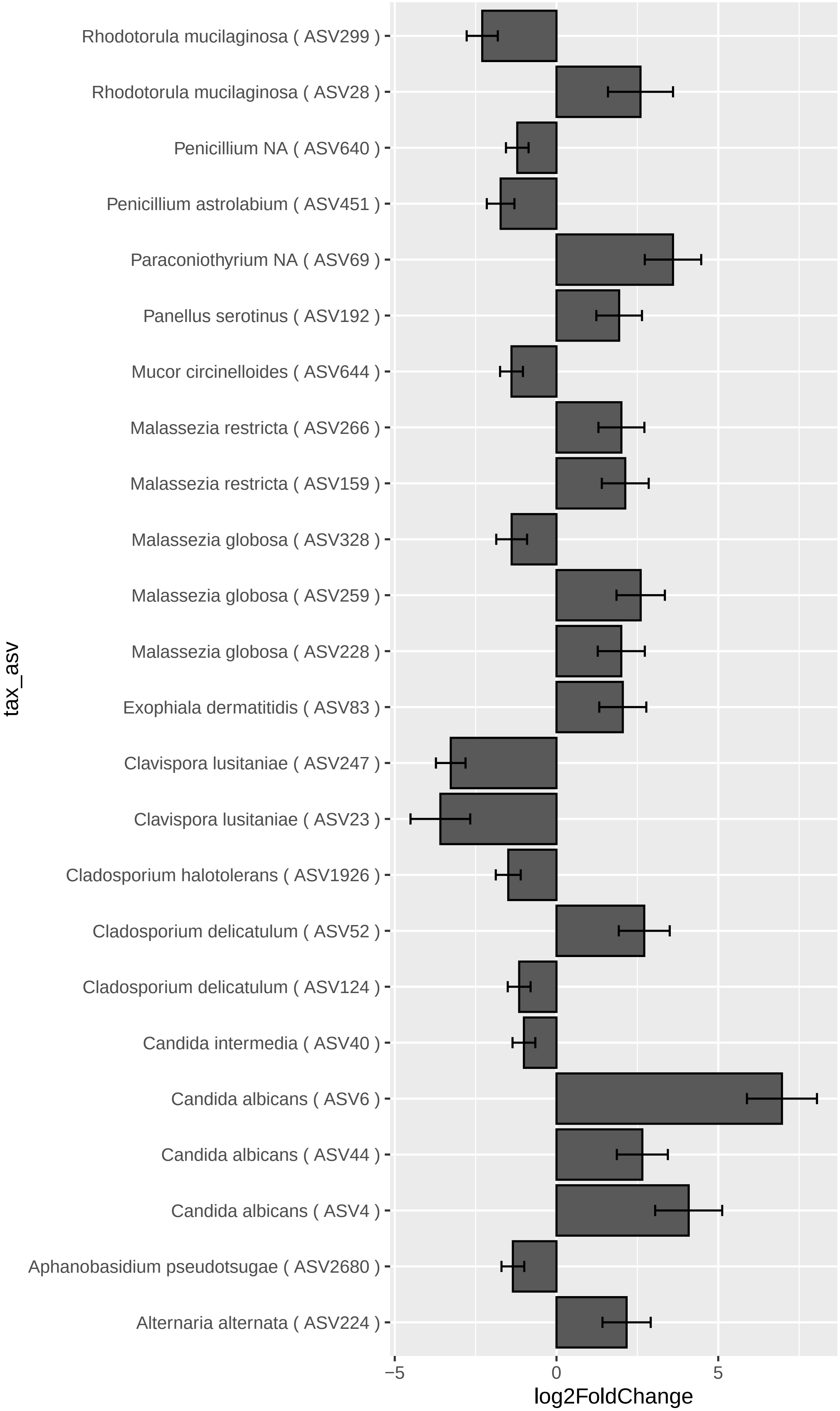
Rhodotorula mucilaginosa (ASV299)
Rhodotorula mucilaginosa (ASV28)
Penicillium NA (ASV640)
Penicillium astrolabium (ASV451)
Paraconiothyrium NA (ASV69)
Panellus serotinus (ASV192)
Mucor circinelloides (ASV644)
Malassezia restricta (ASV266)
Malassezia restricta (ASV159)
Malassezia globosa (ASV328)
Malassezia globosa (ASV259)
Malassezia globosa (ASV228)
Exophiala dermatitidis (ASV83)
Clavispora lusitaniae (ASV247)
Clavispora lusitaniae (ASV23)
Cladosporium halotolerans (ASV1926)
Cladosporium delicatulum (ASV52)
Cladosporium delicatulum (ASV124)
Candida intermedia (ASV40)
Candida albicans (ASV6)
Candida albicans (ASV44)
Candida albicans (ASV4)
Aphanobasidium pseudotsugae (ASV2680)
Alternaria alternata (ASV224)
tax_asv
−5
0
5
log2FoldChange

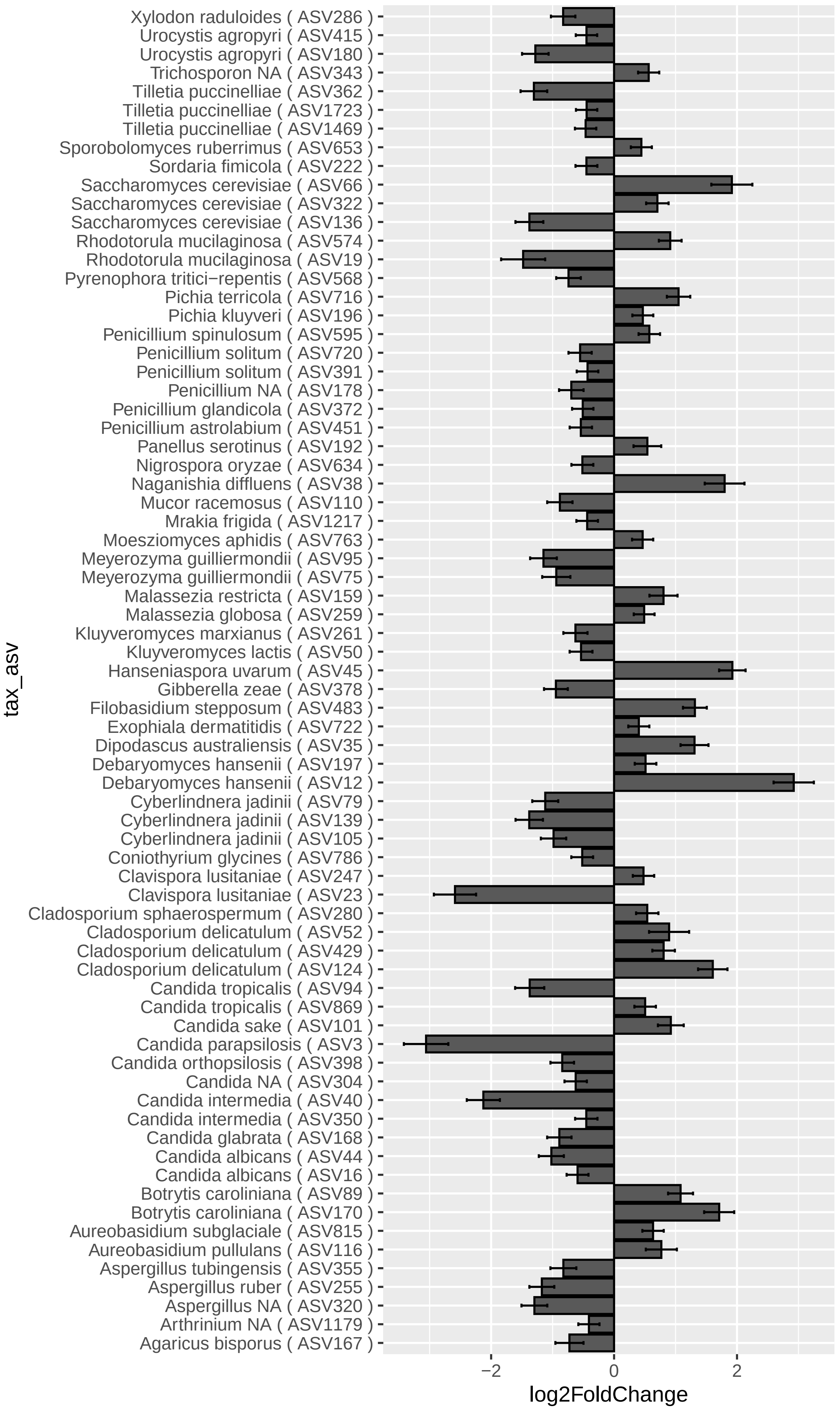

Xylodon raduloides (ASV286)
Urocystis agropyri (ASV415)
Urocystis agropyri (ASV180)
Trichosporon NA (ASV343)
Tilletia puccinelliae (ASV362)
Tilletia puccinelliae (ASV1723)
Tilletia puccinelliae (ASV1469)
Sporobolomyces ruberrimus (ASV653)
Sordaria fimicola (ASV222)
Saccharomyces cerevisiae (ASV66)
Saccharomyces cerevisiae (ASV322)
Saccharomyces cerevisiae (ASV136)
Rhodotorula mucilaginosa (ASV574)
Rhodotorula mucilaginosa (ASV19)
Pyrenophora tritici−repentis (ASV568)
Pichia terricola (ASV716)
Pichia kluyveri (ASV196)
Penicillium spinulosum (ASV595)
Penicillium solitum (ASV720)
Penicillium solitum (ASV391)
Penicillium NA (ASV178)
Penicillium glandicola (ASV372)
Penicillium astrolabium (ASV451)
Panellus serotinus (ASV192)
Nigrospora oryzae (ASV634)
Naganishia diffluens (ASV38)
Mucor racemosus (ASV110)
Mrakia frigida (ASV1217)
Moesziomyces aphidis (ASV763)
Meyerozyma guilliermondii (ASV95)
Meyerozyma guilliermondii (ASV75)
Malassezia restricta (ASV159)
Malassezia globosa (ASV259)
Kluyveromyces marxianus (ASV261)
Kluyveromyces lactis (ASV50)
Hanseniaspora uvarum (ASV45)
Gibberella zeae (ASV378)
Filobasidium stepposum (ASV483)
Exophiala dermatitidis (ASV722)
Dipodascus australiensis (ASV35)
Debaryomyces hansenii (ASV197)
Debaryomyces hansenii (ASV12)
Cyberlindnera jadinii (ASV79)
Cyberlindnera jadinii (ASV139)
Cyberlindnera jadinii (ASV105)
Coniothyrium glycines (ASV786)
Clavispora lusitaniae (ASV247)
Clavispora lusitaniae (ASV23)
Cladosporium sphaerospermum (ASV280)
Cladosporium delicatulum (ASV52)
Cladosporium delicatulum (ASV429)
Cladosporium delicatulum (ASV124)
Candida tropicalis (ASV94)
Candida tropicalis (ASV869)
Candida sake (ASV101)
Candida parapsilosis (ASV3)
Candida orthopsilosis (ASV398)
Candida NA (ASV304)
Candida intermedia (ASV40)
Candida intermedia (ASV350)
Candida glabrata (ASV168)
Candida albicans (ASV44)
Candida albicans (ASV16)
Botrytis caroliniana (ASV89)
Botrytis caroliniana (ASV170)
Aureobasidium subglaciale (ASV815)
Aureobasidium pullulans (ASV116)
Aspergillus tubingensis (ASV355)
Aspergillus ruber (ASV255)
Aspergillus NA (ASV320)
Arthrinium NA (ASV1179)
Agaricus bisporus (ASV167)
tax_asv
−2
0
2
log2FoldChange

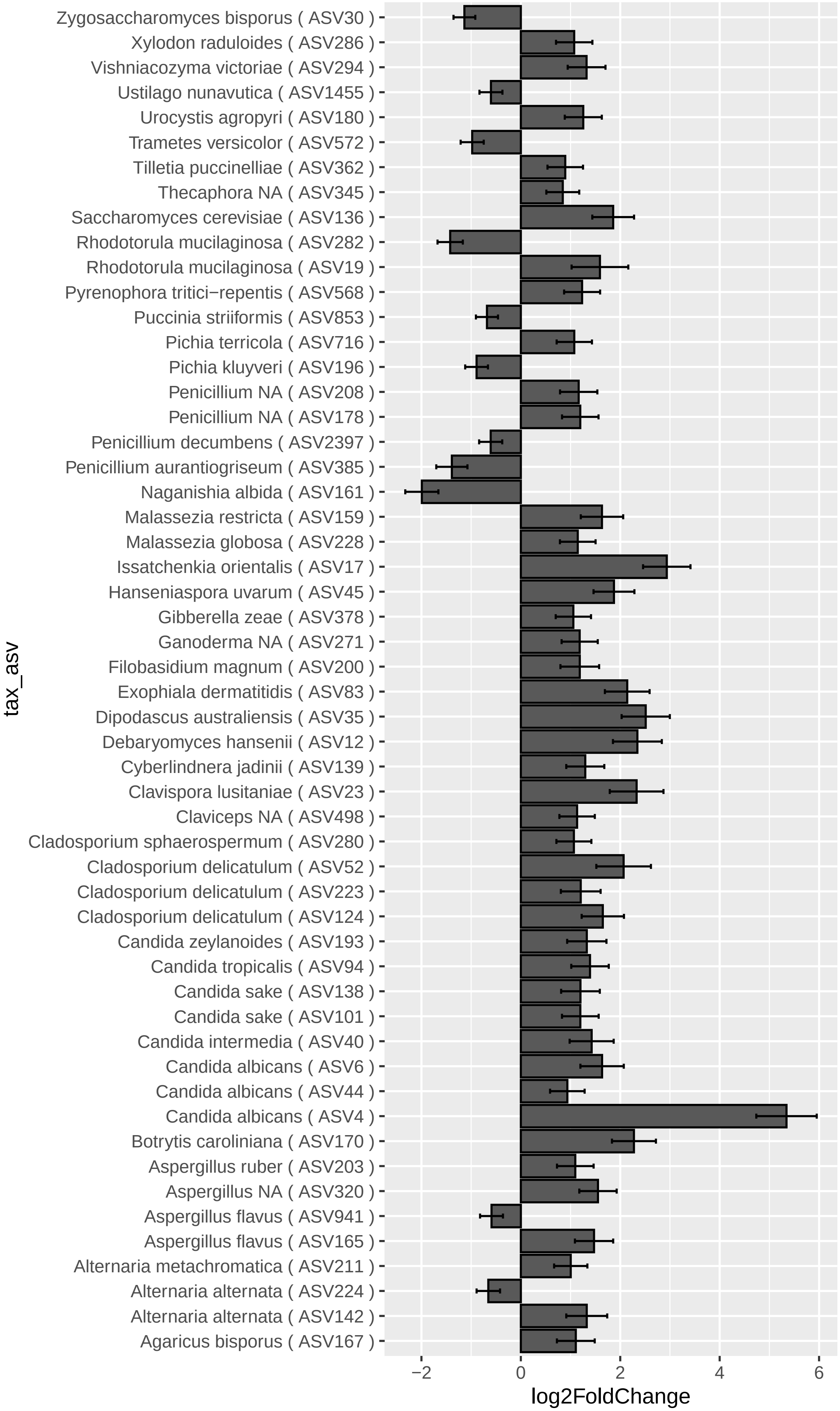

Zygosaccharomyces bisporus (ASV30)
Xylodon raduloides (ASV286)
Vishniacozyma victoriae (ASV294)
Ustilago nunavutica (ASV1455)
Urocystis agropyri (ASV180)
Trametes versicolor (ASV572)
Tilletia puccinelliae (ASV362)
Thecaphora NA (ASV345)
Saccharomyces cerevisiae (ASV136)
Rhodotorula mucilaginosa (ASV282)
Rhodotorula mucilaginosa (ASV19)
Pyrenophora tritici−repentis (ASV568)
Puccinia striiformis (ASV853)
Pichia terricola (ASV716)
Pichia kluyveri (ASV196)
Penicillium NA (ASV208)
Penicillium NA (ASV178)
Penicillium decumbens (ASV2397)
Penicillium aurantiogriseum (ASV385)
Naganishia albida (ASV161)
Malassezia restricta (ASV159)
Malassezia globosa (ASV228)
Issatchenkia orientalis (ASV17)
Hanseniaspora uvarum (ASV45)
Gibberella zeae (ASV378)
Ganoderma NA (ASV271)
Filobasidium magnum (ASV200)
Exophiala dermatitidis (ASV83)
Dipodascus australiensis (ASV35)
Debaryomyces hansenii (ASV12)
Cyberlindnera jadinii (ASV139)
Clavispora lusitaniae (ASV23)
Claviceps NA (ASV498)
Cladosporium sphaerospermum (ASV280)
Cladosporium delicatulum (ASV52)
Cladosporium delicatulum (ASV223)
Cladosporium delicatulum (ASV124)
Candida zeylanoides (ASV193)
Candida tropicalis (ASV94)
Candida sake (ASV138)
Candida sake (ASV101)
Candida intermedia (ASV40)
Candida albicans (ASV6)
Candida albicans (ASV44)
Candida albicans (ASV4)
Botrytis caroliniana (ASV170)
Aspergillus ruber (ASV203)
Aspergillus NA (ASV320)
Aspergillus flavus (ASV941)
Aspergillus flavus (ASV165)
Alternaria metachromatica (ASV211)
Alternaria alternata (ASV224)
Alternaria alternata (ASV142)
Agaricus bisporus (ASV167)
tax_asv
−2
0
2
4
6
log2FoldChange

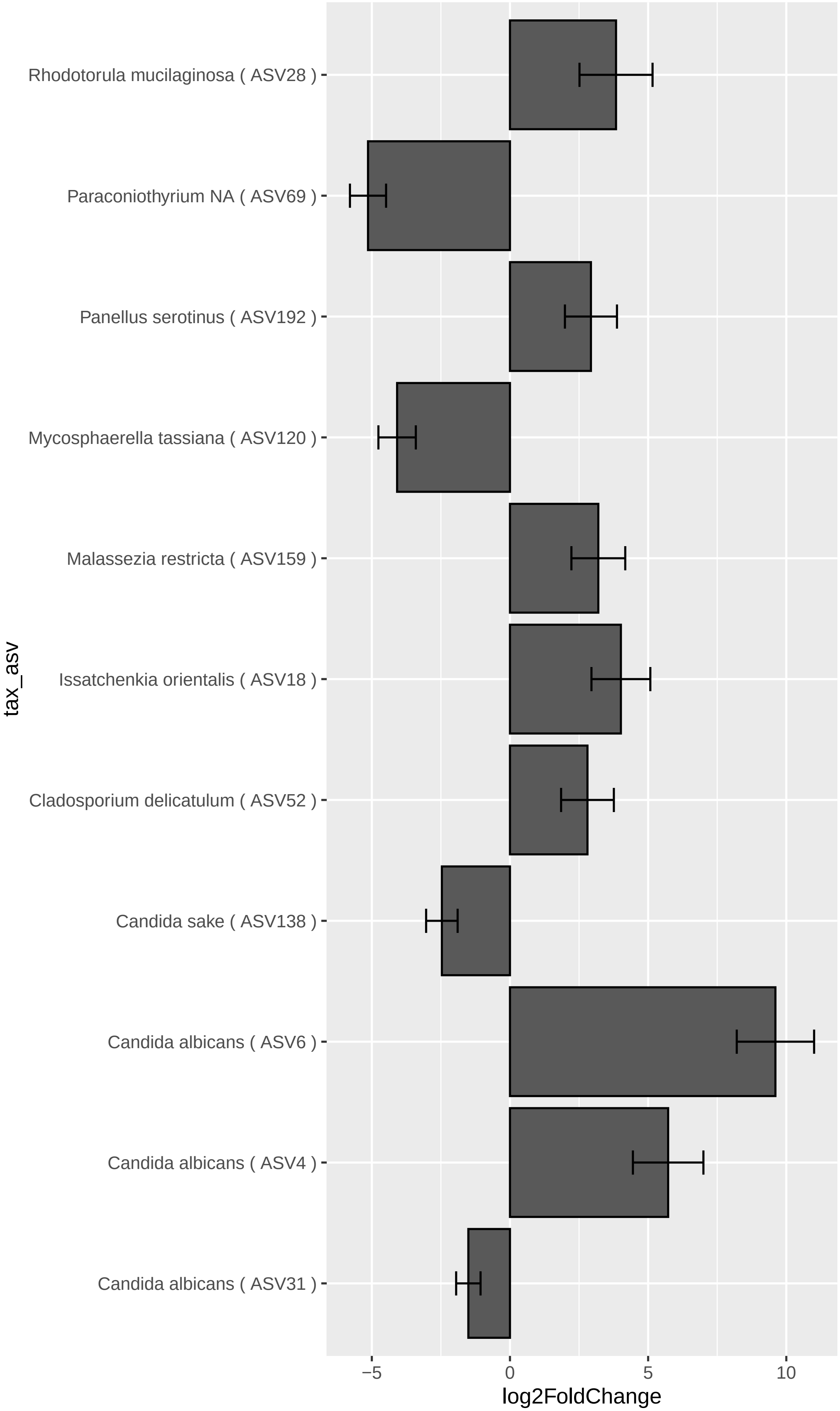

Rhodotorula mucilaginosa (ASV28)
Paraconiothyrium NA (ASV69)
Panellus serotinus (ASV192)
Mycosphaerella tassiana (ASV120)
Malassezia restricta (ASV159)
Issatchenkia orientalis (ASV18)
Cladosporium delicatulum (ASV52)
Candida sake (ASV138)
Candida albicans (ASV6)
Candida albicans (ASV4)
Candida albicans (ASV31)
tax_asv
−5
0
5
10
log2FoldChange

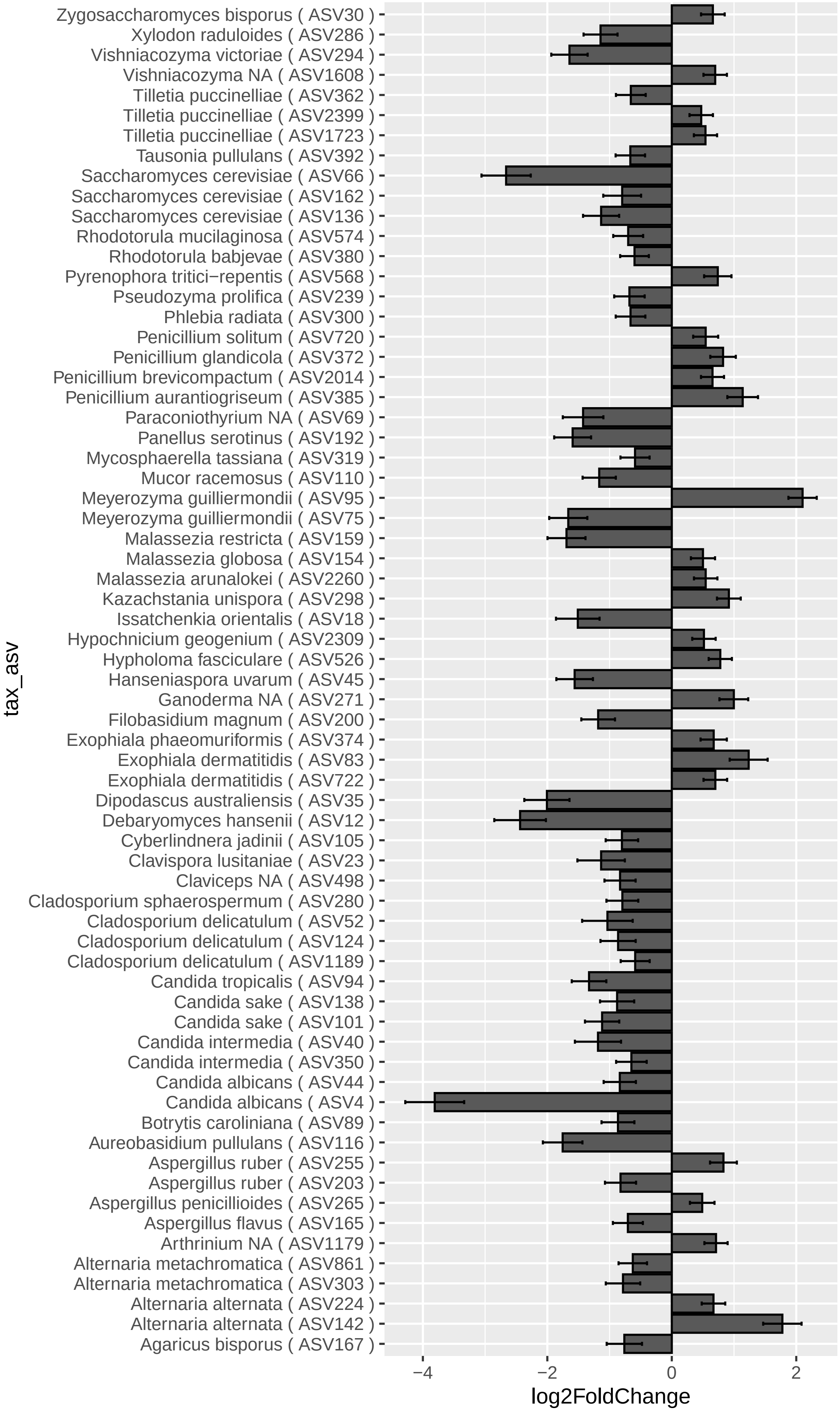
Zygosaccharomyces bisporus (ASV30)
Xylodon raduloides (ASV286)
Vishniacozyma victoriae (ASV294)
Vishniacozyma NA (ASV1608)
Tilletia puccinelliae (ASV362)
Tilletia puccinelliae (ASV2399)
Tilletia puccinelliae (ASV1723)
Tausonia pullulans (ASV392)
Saccharomyces cerevisiae (ASV66)
Saccharomyces cerevisiae (ASV162)
Saccharomyces cerevisiae (ASV136)
Rhodotorula mucilaginosa (ASV574)
Rhodotorula babjevae (ASV380)
Pyrenophora tritici−repentis (ASV568)
Pseudozyma prolifica (ASV239)
Phlebia radiata (ASV300)
Penicillium solitum (ASV720)
Penicillium glandicola (ASV372)
Penicillium brevicompactum (ASV2014)
Penicillium aurantiogriseum (ASV385)
Paraconiothyrium NA (ASV69)
Panellus serotinus (ASV192)
Mycosphaerella tassiana (ASV319)
Mucor racemosus (ASV110)
Meyerozyma guilliermondii (ASV95)
Meyerozyma guilliermondii (ASV75)
Malassezia restricta (ASV159)
Malassezia globosa (ASV154)
Malassezia arunalokei (ASV2260)
Kazachstania unispora (ASV298)
Issatchenkia orientalis (ASV18)
Hypochnicium geogenium (ASV2309)
Hypholoma fasciculare (ASV526)
Hanseniaspora uvarum (ASV45)
Ganoderma NA (ASV271)
Filobasidium magnum (ASV200)
Exophiala phaeomuriformis (ASV374)
Exophiala dermatitidis (ASV83)
Exophiala dermatitidis (ASV722)
Dipodascus australiensis (ASV35)
Debaryomyces hansenii (ASV12)
Cyberlindnera jadinii (ASV105)
Clavispora lusitaniae (ASV23)
Claviceps NA (ASV498)
Cladosporium sphaerospermum (ASV280)
Cladosporium delicatulum (ASV52)
Cladosporium delicatulum (ASV124)
Cladosporium delicatulum (ASV1189)
Candida tropicalis (ASV94)
Candida sake (ASV138)
Candida sake (ASV101)
Candida intermedia (ASV40)
Candida intermedia (ASV350)
Candida albicans (ASV44)
Candida albicans (ASV4)
Botrytis caroliniana (ASV89)
Aureobasidium pullulans (ASV116)
Aspergillus ruber (ASV255)
Aspergillus ruber (ASV203)
Aspergillus penicillioides (ASV265)
Aspergillus flavus (ASV165)
Arthrinium NA (ASV1179)
Alternaria metachromatica (ASV861)
Alternaria metachromatica (ASV303)
Alternaria alternata (ASV224)
Alternaria alternata (ASV142)
Agaricus bisporus (ASV167)
tax_asv
−4
−2
0
2
log2FoldChange

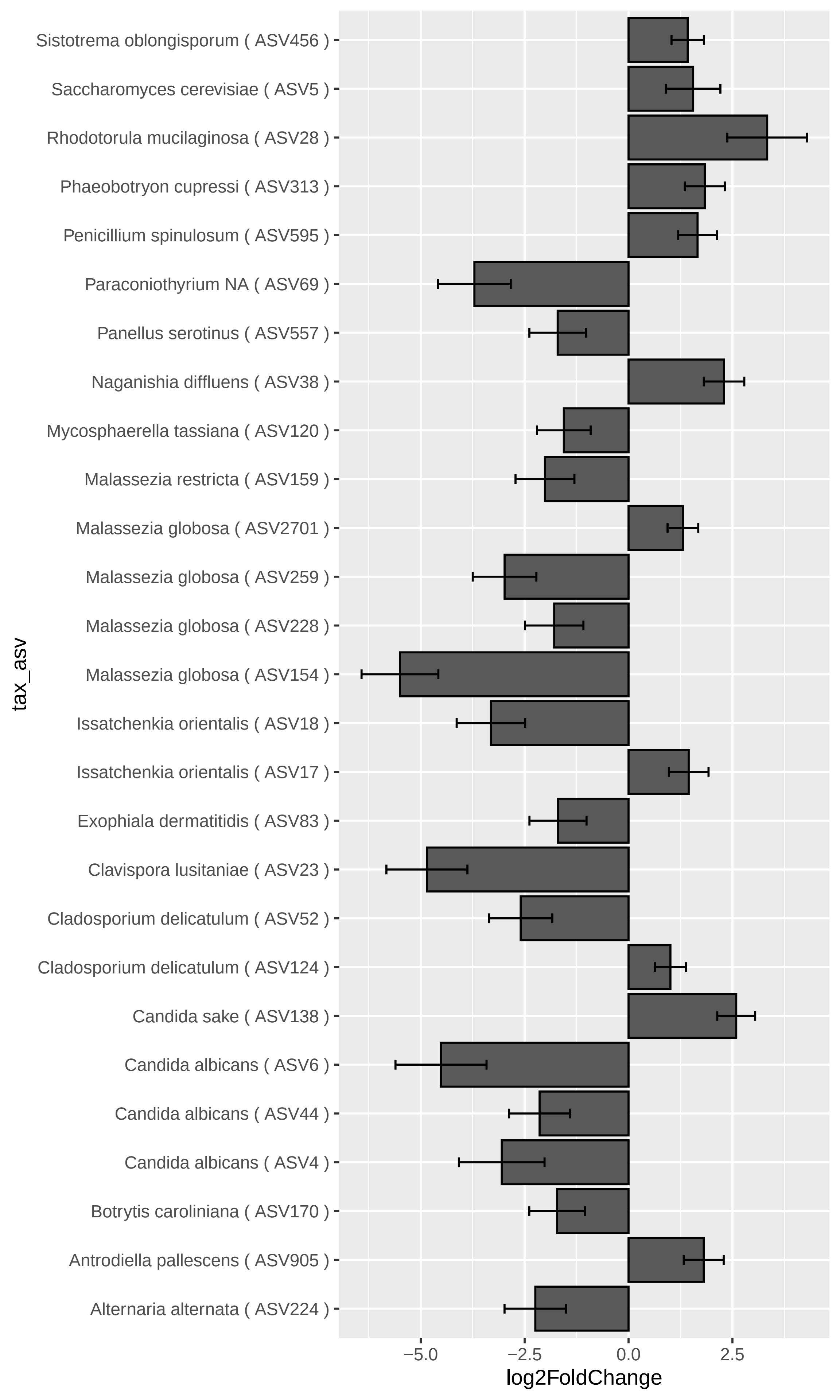
Sistotrema oblongisporum (ASV456)
Saccharomyces cerevisiae (ASV5)
Rhodotorula mucilaginosa (ASV28)
Phaeobotryon cupressi (ASV313)
Penicillium spinulosum (ASV595)
Paraconiothyrium NA (ASV69)
Panellus serotinus (ASV557)
Naganishia diffluens (ASV38)
Mycosphaerella tassiana (ASV120)
Malassezia restricta (ASV159)
Malassezia globosa (ASV2701)
Malassezia globosa (ASV259)
Malassezia globosa (ASV228)
Malassezia globosa (ASV154)
Issatchenkia orientalis (ASV18)
Issatchenkia orientalis (ASV17)
Exophiala dermatitidis (ASV83)
Clavispora lusitaniae (ASV23)
Cladosporium delicatulum (ASV52)
Cladosporium delicatulum (ASV124)
Candida sake (ASV138)
Candida albicans (ASV6)
Candida albicans (ASV44)
Candida albicans (ASV4)
Botrytis caroliniana (ASV170)
Antrodiella pallescens (ASV905)
Alternaria alternata (ASV224)
tax_asv
−5.0
−2.5
0.0
2.5
log2FoldChange

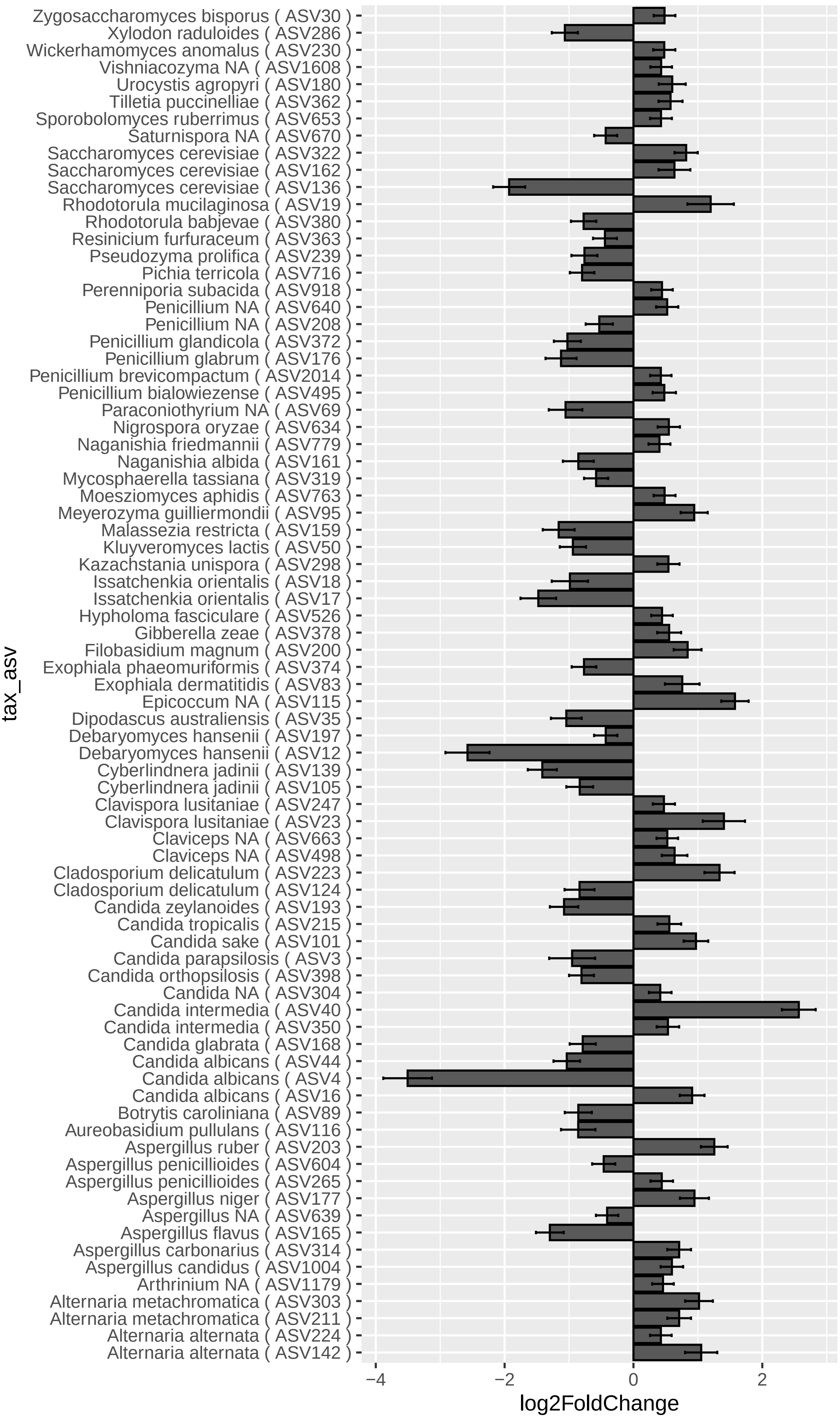
Zygosaccharomyces bisporus (ASV30)
Xylodon raduloides (ASV286)
Wickerhamomyces anomalus (ASV230)
Vishniacozyma NA (ASV1608)
Urocystis agropyri (ASV180)
Tilletia puccinelliae (ASV362)
Sporobolomyces ruberrimus (ASV653)
Saturnispora NA (ASV670)
Saccharomyces cerevisiae (ASV322)
Saccharomyces cerevisiae (ASV162)
Saccharomyces cerevisiae (ASV136)
Rhodotorula mucilaginosa (ASV19)
Rhodotorula babjevae (ASV380)
Resinicium furfuraceum (ASV363)
Pseudozyma prolifica (ASV239)
Pichia terricola (ASV716)
Perenniporia subacida (ASV918)
Penicillium NA (ASV640)
Penicillium NA (ASV208)
Penicillium glandicola (ASV372)
Penicillium glabrum (ASV176)
Penicillium brevicompactum (ASV2014)
Penicillium bialowiezense (ASV495)
Paraconiothyrium NA (ASV69)
Nigrospora oryzae (ASV634)
Naganishia friedmannii (ASV779)
Naganishia albida (ASV161)
Mycosphaerella tassiana (ASV319)
Moesziomyces aphidis (ASV763)
Meyerozyma guilliermondii (ASV95)
Malassezia restricta (ASV159)
Kluyveromyces lactis (ASV50)
Kazachstania unispora (ASV298)
Issatchenkia orientalis (ASV18)
Issatchenkia orientalis (ASV17)
Hypholoma fasciculare (ASV526)
Gibberella zeae (ASV378)
Filobasidium magnum (ASV200)
Exophiala phaeomuriformis (ASV374)
Exophiala dermatitidis (ASV83)
Epicoccum NA (ASV115)
Dipodascus australiensis (ASV35)
Debaryomyces hansenii (ASV197)
Debaryomyces hansenii (ASV12)
Cyberlindnera jadinii (ASV139)
Cyberlindnera jadinii (ASV105)
Clavispora lusitaniae (ASV247)
Clavispora lusitaniae (ASV23)
Claviceps NA (ASV663)
Claviceps NA (ASV498)
Cladosporium delicatulum (ASV223)
Cladosporium delicatulum (ASV124)
Candida zeylanoides (ASV193)
Candida tropicalis (ASV215)
Candida sake (ASV101)
Candida parapsilosis (ASV3)
Candida orthopsilosis (ASV398)
Candida NA (ASV304)
Candida intermedia (ASV40)
Candida intermedia (ASV350)
Candida glabrata (ASV168)
Candida albicans (ASV44)
Candida albicans (ASV4)
Candida albicans (ASV16)
Botrytis caroliniana (ASV89)
Aureobasidium pullulans (ASV116)
Aspergillus ruber (ASV203)
Aspergillus penicillioides (ASV604)
Aspergillus penicillioides (ASV265)
Aspergillus niger (ASV177)
Aspergillus NA (ASV639)
Aspergillus flavus (ASV165)
Aspergillus carbonarius (ASV314)
Aspergillus candidus (ASV1004)
Arthrinium NA (ASV1179)
Alternaria metachromatica (ASV303)
Alternaria metachromatica (ASV211)
Alternaria alternata (ASV224)
Alternaria alternata (ASV142)
tax_asv
−4
−2
0
2
log2FoldChange

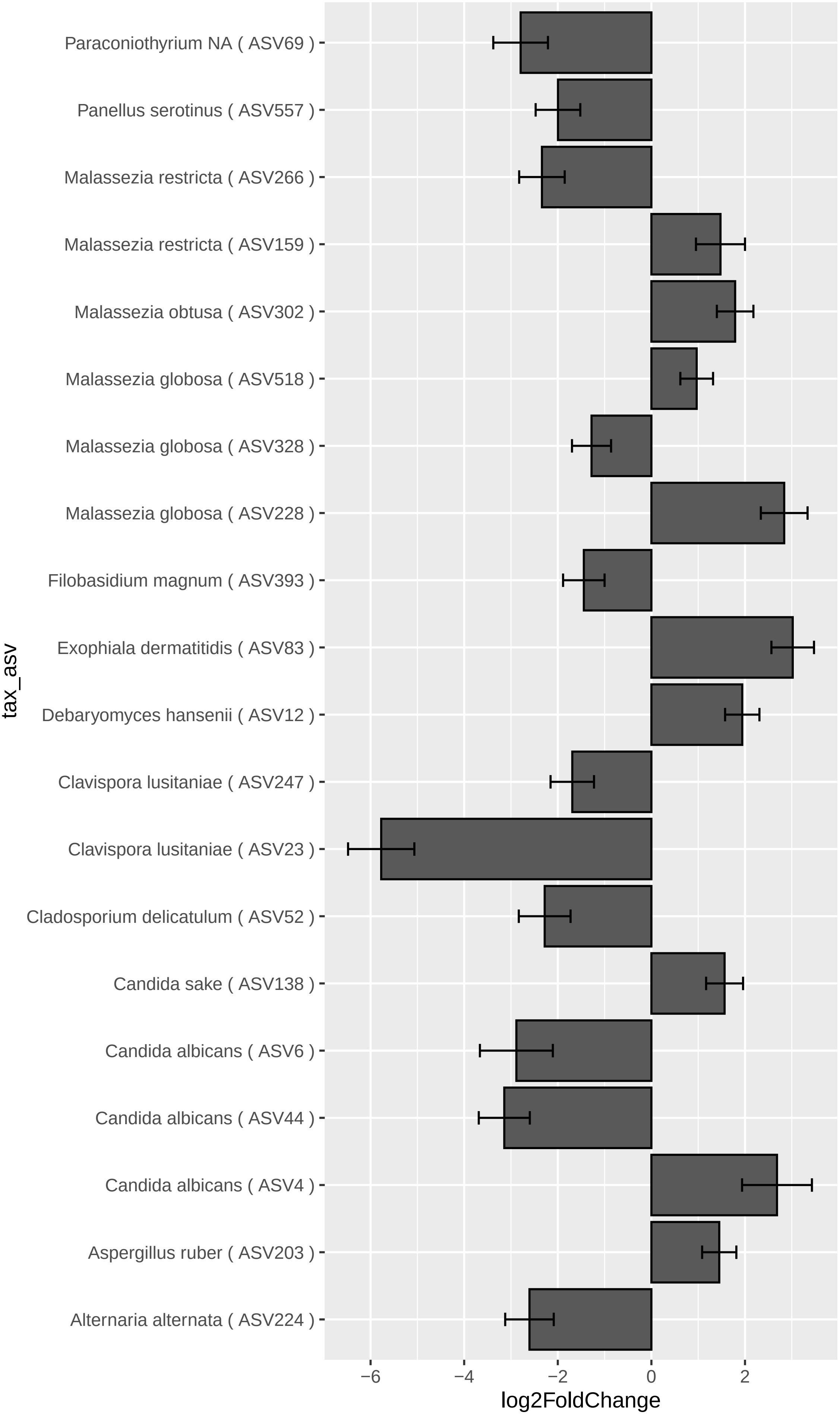
Paraconiothyrium NA (ASV69)
Panellus serotinus (ASV557)
Malassezia restricta (ASV266)
Malassezia restricta (ASV159)
Malassezia obtusa (ASV302)
Malassezia globosa (ASV518)
Malassezia globosa (ASV328)
Malassezia globosa (ASV228)
Filobasidium magnum (ASV393)
Exophiala dermatitidis (ASV83)
Debaryomyces hansenii (ASV12)
Clavispora lusitaniae (ASV247)
Clavispora lusitaniae (ASV23)
Cladosporium delicatulum (ASV52)
Candida sake (ASV138)
Candida albicans (ASV6)
Candida albicans (ASV44)
Candida albicans (ASV4)
Aspergillus ruber (ASV203)
Alternaria alternata (ASV224)
tax_asv
−6
−4
−2
0
2
log2FoldChange

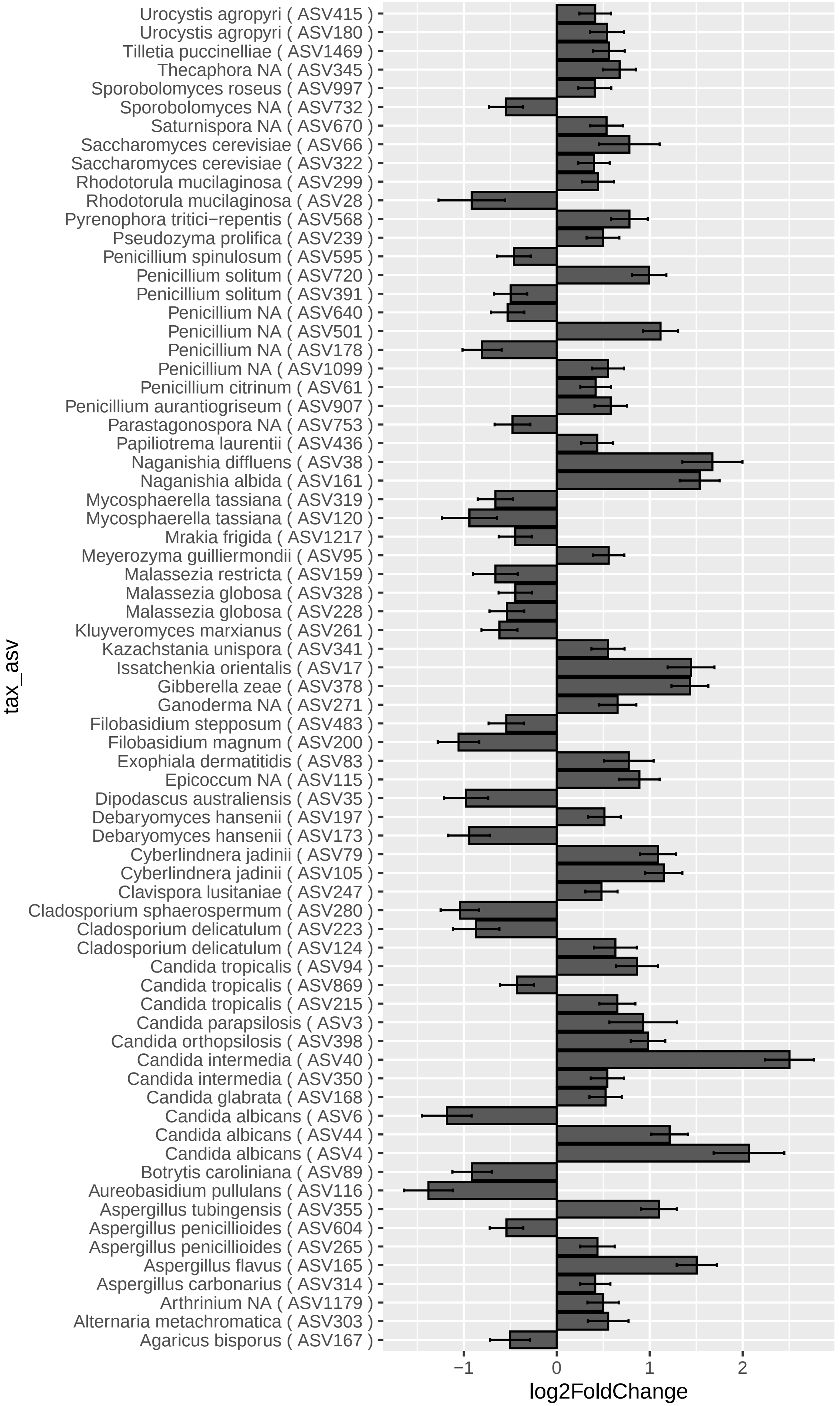
Urocystis agropyri (ASV415)
Urocystis agropyri (ASV180)
Tilletia puccinelliae (ASV1469)
Thecaphora NA (ASV345)
Sporobolomyces roseus (ASV997)
Sporobolomyces NA (ASV732)
Saturnispora NA (ASV670)
Saccharomyces cerevisiae (ASV66)
Saccharomyces cerevisiae (ASV322)
Rhodotorula mucilaginosa (ASV299)
Rhodotorula mucilaginosa (ASV28)
Pyrenophora tritici−repentis (ASV568)
Pseudozyma prolifica (ASV239)
Penicillium spinulosum (ASV595)
Penicillium solitum (ASV720)
Penicillium solitum (ASV391)
Penicillium NA (ASV640)
Penicillium NA (ASV501)
Penicillium NA (ASV178)
Penicillium NA (ASV1099)
Penicillium citrinum (ASV61)
Penicillium aurantiogriseum (ASV907)
Parastagonospora NA (ASV753)
Papiliotrema laurentii (ASV436)
Naganishia diffluens (ASV38)
Naganishia albida (ASV161)
Mycosphaerella tassiana (ASV319)
Mycosphaerella tassiana (ASV120)
Mrakia frigida (ASV1217)
Meyerozyma guilliermondii (ASV95)
Malassezia restricta (ASV159)
Malassezia globosa (ASV328)
Malassezia globosa (ASV228)
Kluyveromyces marxianus (ASV261)
Kazachstania unispora (ASV341)
Issatchenkia orientalis (ASV17)
Gibberella zeae (ASV378)
Ganoderma NA (ASV271)
Filobasidium stepposum (ASV483)
Filobasidium magnum (ASV200)
Exophiala dermatitidis (ASV83)
Epicoccum NA (ASV115)
Dipodascus australiensis (ASV35)
Debaryomyces hansenii (ASV197)
Debaryomyces hansenii (ASV173)
Cyberlindnera jadinii (ASV79)
Cyberlindnera jadinii (ASV105)
Clavispora lusitaniae (ASV247)
Cladosporium sphaerospermum (ASV280)
Cladosporium delicatulum (ASV223)
Cladosporium delicatulum (ASV124)
Candida tropicalis (ASV94)
Candida tropicalis (ASV869)
Candida tropicalis (ASV215)
Candida parapsilosis (ASV3)
Candida orthopsilosis (ASV398)
Candida intermedia (ASV40)
Candida intermedia (ASV350)
Candida glabrata (ASV168)
Candida albicans (ASV6)
Candida albicans (ASV44)
Candida albicans (ASV4)
Botrytis caroliniana (ASV89)
Aureobasidium pullulans (ASV116)
Aspergillus tubingensis (ASV355)
Aspergillus penicillioides (ASV604)
Aspergillus penicillioides (ASV265)
Aspergillus flavus (ASV165)
Aspergillus carbonarius (ASV314)
Arthrinium NA (ASV1179)
Alternaria metachromatica (ASV303)
Agaricus bisporus (ASV167)
tax_asv
−1
0
1
2
log2FoldChange

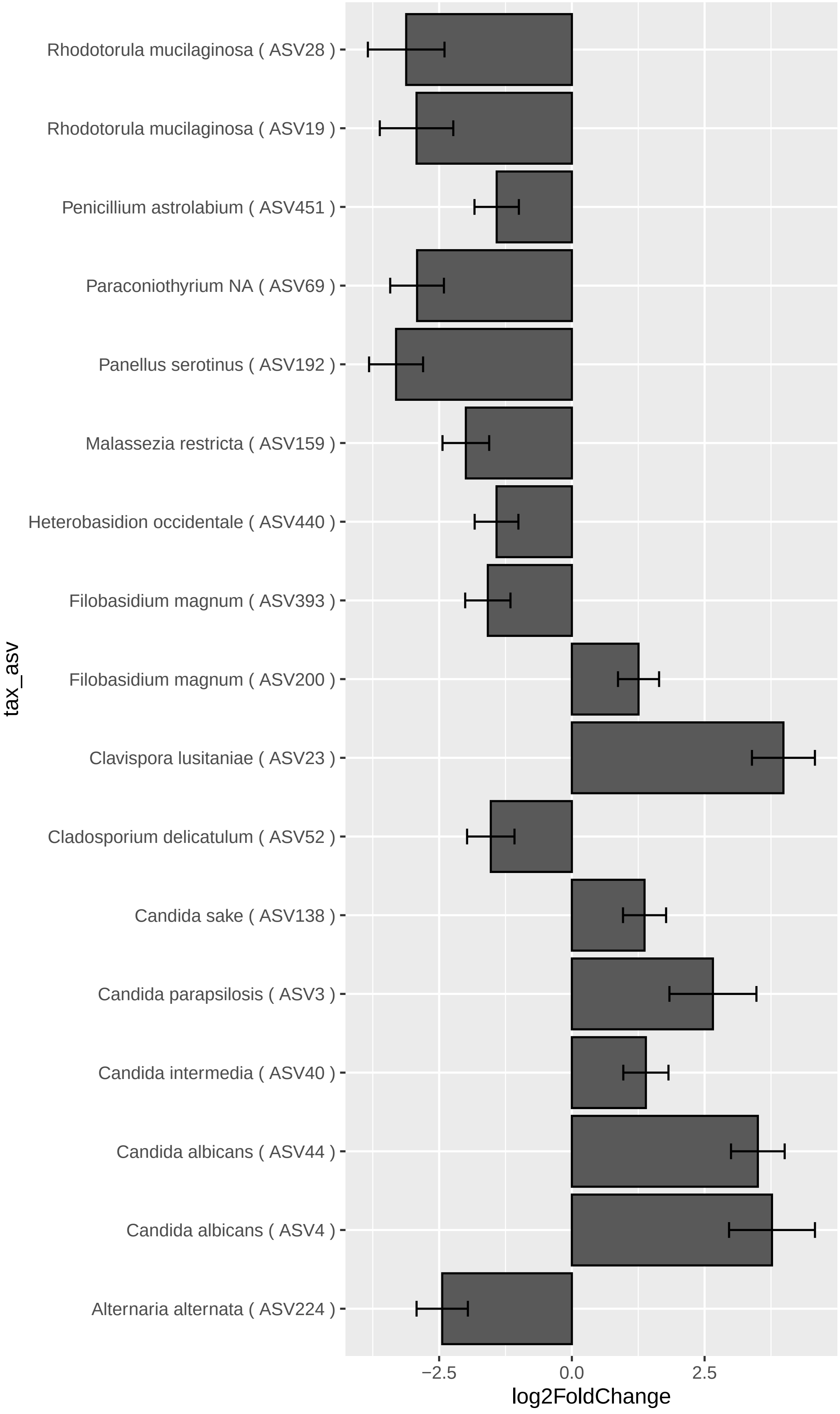
Rhodotorula mucilaginosa (ASV28)
Rhodotorula mucilaginosa (ASV19)
Penicillium astrolabium (ASV451)
Paraconiothyrium NA (ASV69)
Panellus serotinus (ASV192)
Malassezia restricta (ASV159)
Heterobasidion occidentale (ASV440)
Filobasidium magnum (ASV393)
Filobasidium magnum (ASV200)
Clavispora lusitaniae (ASV23)
Cladosporium delicatulum (ASV52)
Candida sake (ASV138)
Candida parapsilosis (ASV3)
Candida intermedia (ASV40)
Candida albicans (ASV44)
Candida albicans (ASV4)
Alternaria alternata (ASV224)
tax_asv
−2.5
0.0
2.5
log2FoldChange

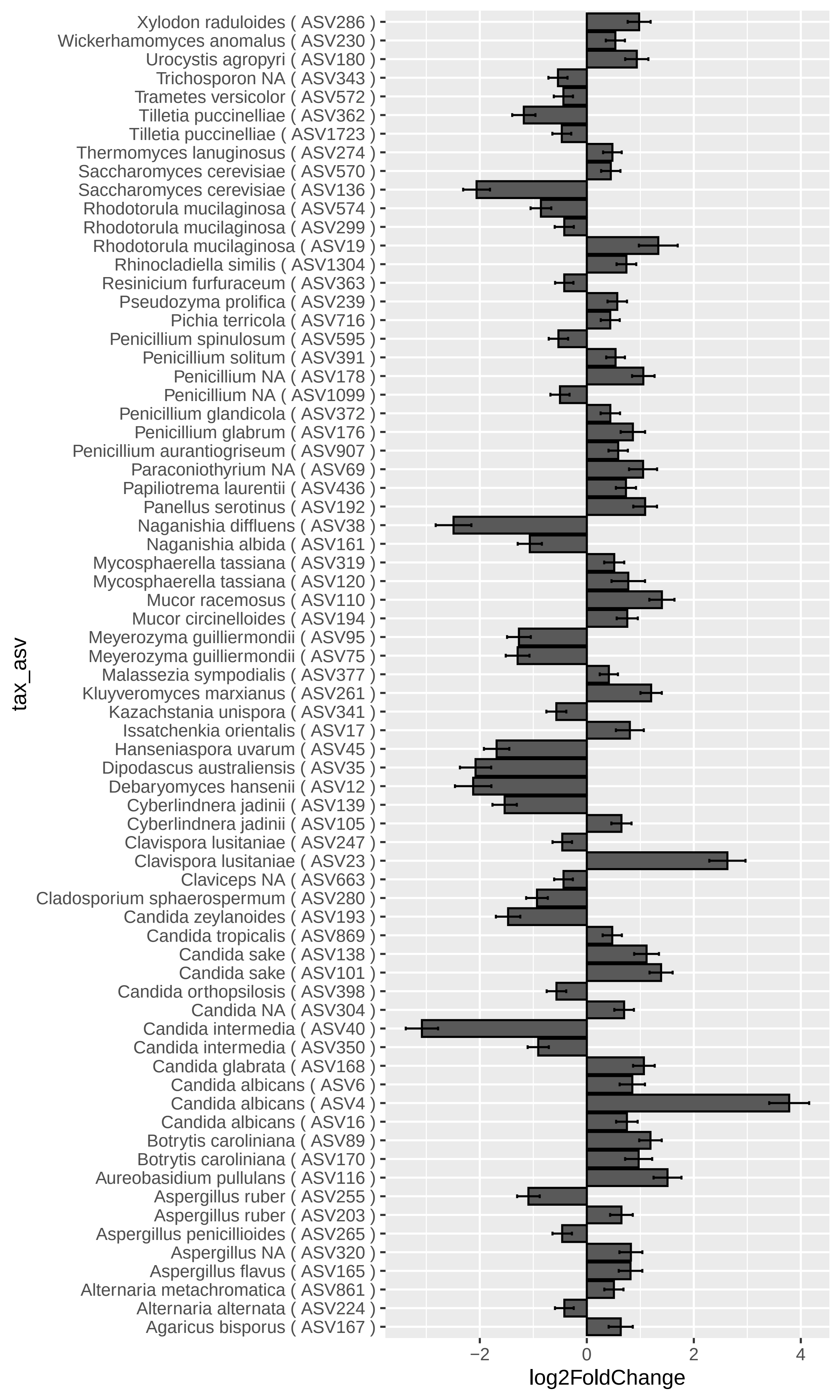
Xylodon raduloides (ASV286)
Wickerhamomyces anomalus (ASV230)
Urocystis agropyri (ASV180)
Trichosporon NA (ASV343)
Trametes versicolor (ASV572)
Tilletia puccinelliae (ASV362)
Tilletia puccinelliae (ASV1723)
Thermomyces lanuginosus (ASV274)
Saccharomyces cerevisiae (ASV570)
Saccharomyces cerevisiae (ASV136)
Rhodotorula mucilaginosa (ASV574)
Rhodotorula mucilaginosa (ASV299)
Rhodotorula mucilaginosa (ASV19)
Rhinocladiella similis (ASV1304)
Resinicium furfuraceum (ASV363)
Pseudozyma prolifica (ASV239)
Pichia terricola (ASV716)
Penicillium spinulosum (ASV595)
Penicillium solitum (ASV391)
Penicillium NA (ASV178)
Penicillium NA (ASV1099)
Penicillium glandicola (ASV372)
Penicillium glabrum (ASV176)
Penicillium aurantiogriseum (ASV907)
Paraconiothyrium NA (ASV69)
Papiliotrema laurentii (ASV436)
Panellus serotinus (ASV192)
Naganishia diffluens (ASV38)
Naganishia albida (ASV161)
Mycosphaerella tassiana (ASV319)
Mycosphaerella tassiana (ASV120)
Mucor racemosus (ASV110)
Mucor circinelloides (ASV194)
Meyerozyma guilliermondii (ASV95)
Meyerozyma guilliermondii (ASV75)
Malassezia sympodialis (ASV377)
Kluyveromyces marxianus (ASV261)
Kazachstania unispora (ASV341)
Issatchenkia orientalis (ASV17)
Hanseniaspora uvarum (ASV45)
Dipodascus australiensis (ASV35)
Debaryomyces hansenii (ASV12)
Cyberlindnera jadinii (ASV139)
Cyberlindnera jadinii (ASV105)
Clavispora lusitaniae (ASV247)
Clavispora lusitaniae (ASV23)
Claviceps NA (ASV663)
Cladosporium sphaerospermum (ASV280)
Candida zeylanoides (ASV193)
Candida tropicalis (ASV869)
Candida sake (ASV138)
Candida sake (ASV101)
Candida orthopsilosis (ASV398)
Candida NA (ASV304)
Candida intermedia (ASV40)
Candida intermedia (ASV350)
Candida glabrata (ASV168)
Candida albicans (ASV6)
Candida albicans (ASV4)
Candida albicans (ASV16)
Botrytis caroliniana (ASV89)
Botrytis caroliniana (ASV170)
Aureobasidium pullulans (ASV116)
Aspergillus ruber (ASV255)
Aspergillus ruber (ASV203)
Aspergillus penicillioides (ASV265)
Aspergillus NA (ASV320)
Aspergillus flavus (ASV165)
Alternaria metachromatica (ASV861)
Alternaria alternata (ASV224)
Agaricus bisporus (ASV167)
tax_asv
−2
0
2
4
log2FoldChange

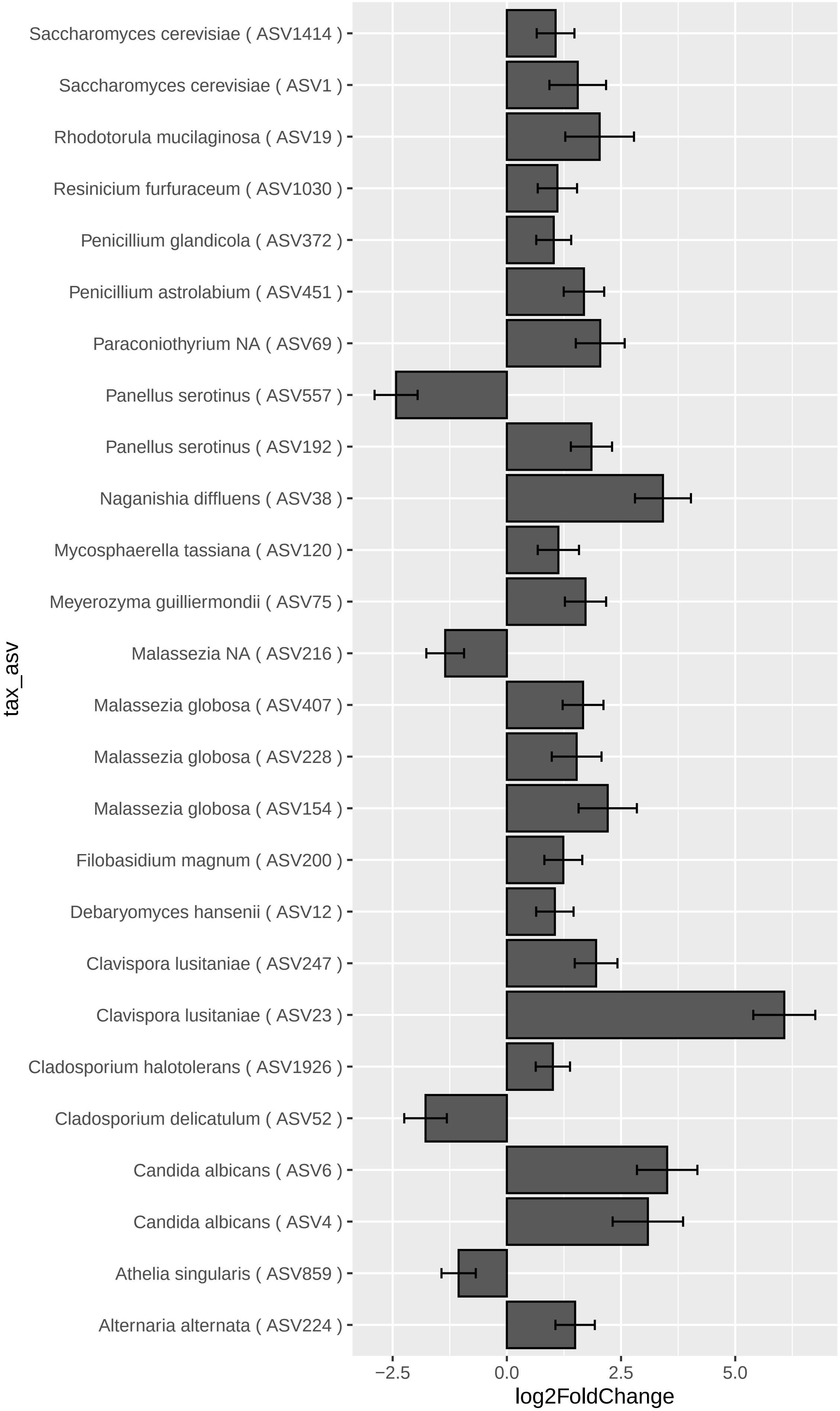

Saccharomyces cerevisiae (ASV1414)
Saccharomyces cerevisiae (ASV1)
Rhodotorula mucilaginosa (ASV19)
Resinicium furfuraceum (ASV1030)
Penicillium glandicola (ASV372)
Penicillium astrolabium (ASV451)
Paraconiothyrium NA (ASV69)
Panellus serotinus (ASV557)
Panellus serotinus (ASV192)
Naganishia diffluens (ASV38)
Mycosphaerella tassiana (ASV120)
Meyerozyma guilliermondii (ASV75)
Malassezia NA (ASV216)
Malassezia globosa (ASV407)
Malassezia globosa (ASV228)
Malassezia globosa (ASV154)
Filobasidium magnum (ASV200)
Debaryomyces hansenii (ASV12)
Clavispora lusitaniae (ASV247)
Clavispora lusitaniae (ASV23)
Cladosporium halotolerans (ASV1926)
Cladosporium delicatulum (ASV52)
Candida albicans (ASV6)
Candida albicans (ASV4)
Athelia singularis (ASV859)
Alternaria alternata (ASV224)
tax_asv
−2.5
0.0
2.5
5.0
log2FoldChange

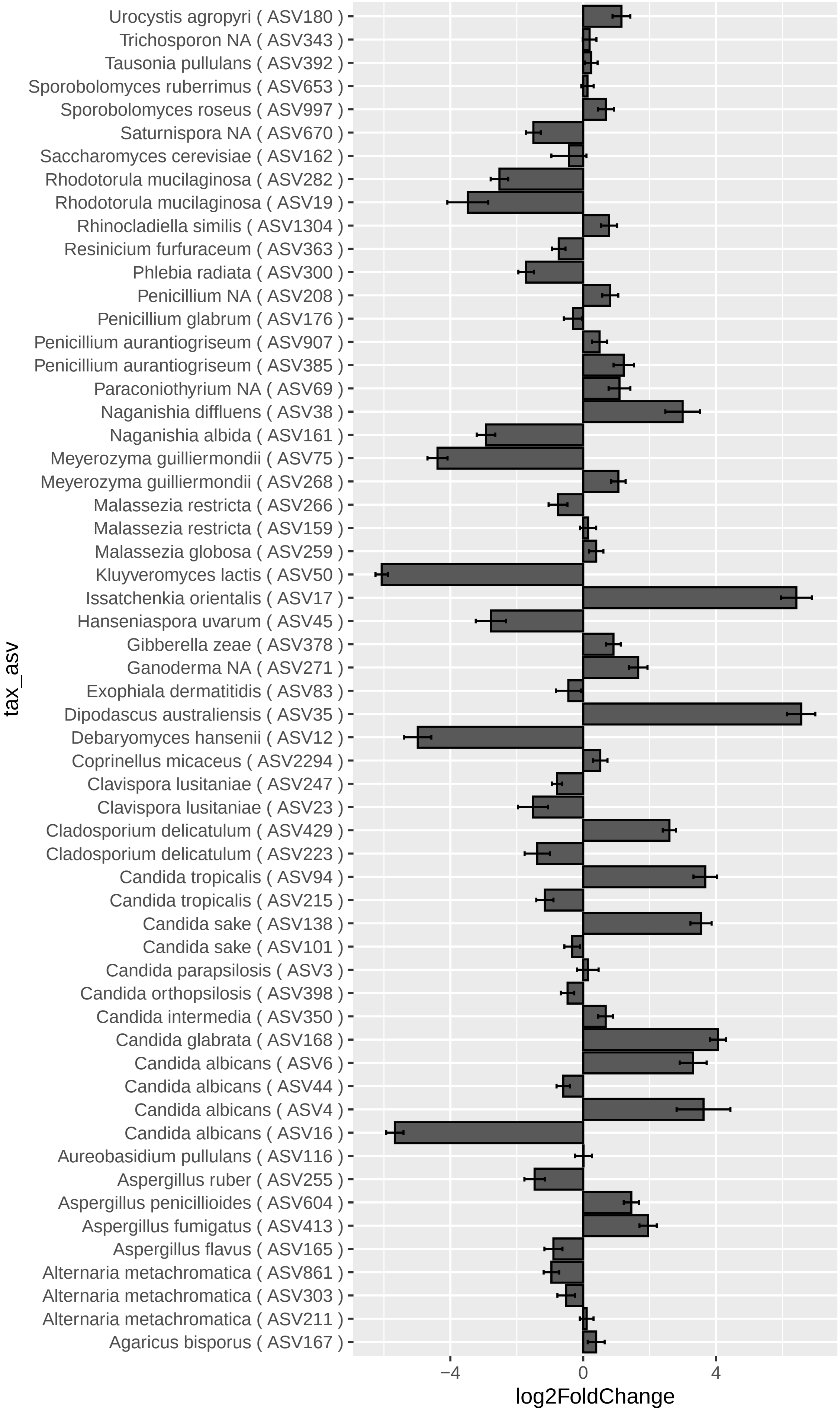
Urocystis agropyri (ASV180)
Trichosporon NA (ASV343)
Tausonia pullulans (ASV392)
Sporobolomyces ruberrimus (ASV653)
Sporobolomyces roseus (ASV997)
Saturnispora NA (ASV670)
Saccharomyces cerevisiae (ASV162)
Rhodotorula mucilaginosa (ASV282)
Rhodotorula mucilaginosa (ASV19)
Rhinocladiella similis (ASV1304)
Resinicium furfuraceum (ASV363)
Phlebia radiata (ASV300)
Penicillium NA (ASV208)
Penicillium glabrum (ASV176)
Penicillium aurantiogriseum (ASV907)
Penicillium aurantiogriseum (ASV385)
Paraconiothyrium NA (ASV69)
Naganishia diffluens (ASV38)
Naganishia albida (ASV161)
Meyerozyma guilliermondii (ASV75)
Meyerozyma guilliermondii (ASV268)
Malassezia restricta (ASV266)
Malassezia restricta (ASV159)
Malassezia globosa (ASV259)
Kluyveromyces lactis (ASV50)
Issatchenkia orientalis (ASV17)
Hanseniaspora uvarum (ASV45)
Gibberella zeae (ASV378)
Ganoderma NA (ASV271)
Exophiala dermatitidis (ASV83)
Dipodascus australiensis (ASV35)
Debaryomyces hansenii (ASV12)
Coprinellus micaceus (ASV2294)
Clavispora lusitaniae (ASV247)
Clavispora lusitaniae (ASV23)
Cladosporium delicatulum (ASV429)
Cladosporium delicatulum (ASV223)
Candida tropicalis (ASV94)
Candida tropicalis (ASV215)
Candida sake (ASV138)
Candida sake (ASV101)
Candida parapsilosis (ASV3)
Candida orthopsilosis (ASV398)
Candida intermedia (ASV350)
Candida glabrata (ASV168)
Candida albicans (ASV6)
Candida albicans (ASV44)
Candida albicans (ASV4)
Candida albicans (ASV16)
Aureobasidium pullulans (ASV116)
Aspergillus ruber (ASV255)
Aspergillus penicillioides (ASV604)
Aspergillus fumigatus (ASV413)
Aspergillus flavus (ASV165)
Alternaria metachromatica (ASV861)
Alternaria metachromatica (ASV303)
Alternaria metachromatica (ASV211)
Agaricus bisporus (ASV167)
tax_asv
−4
0
4
log2FoldChange

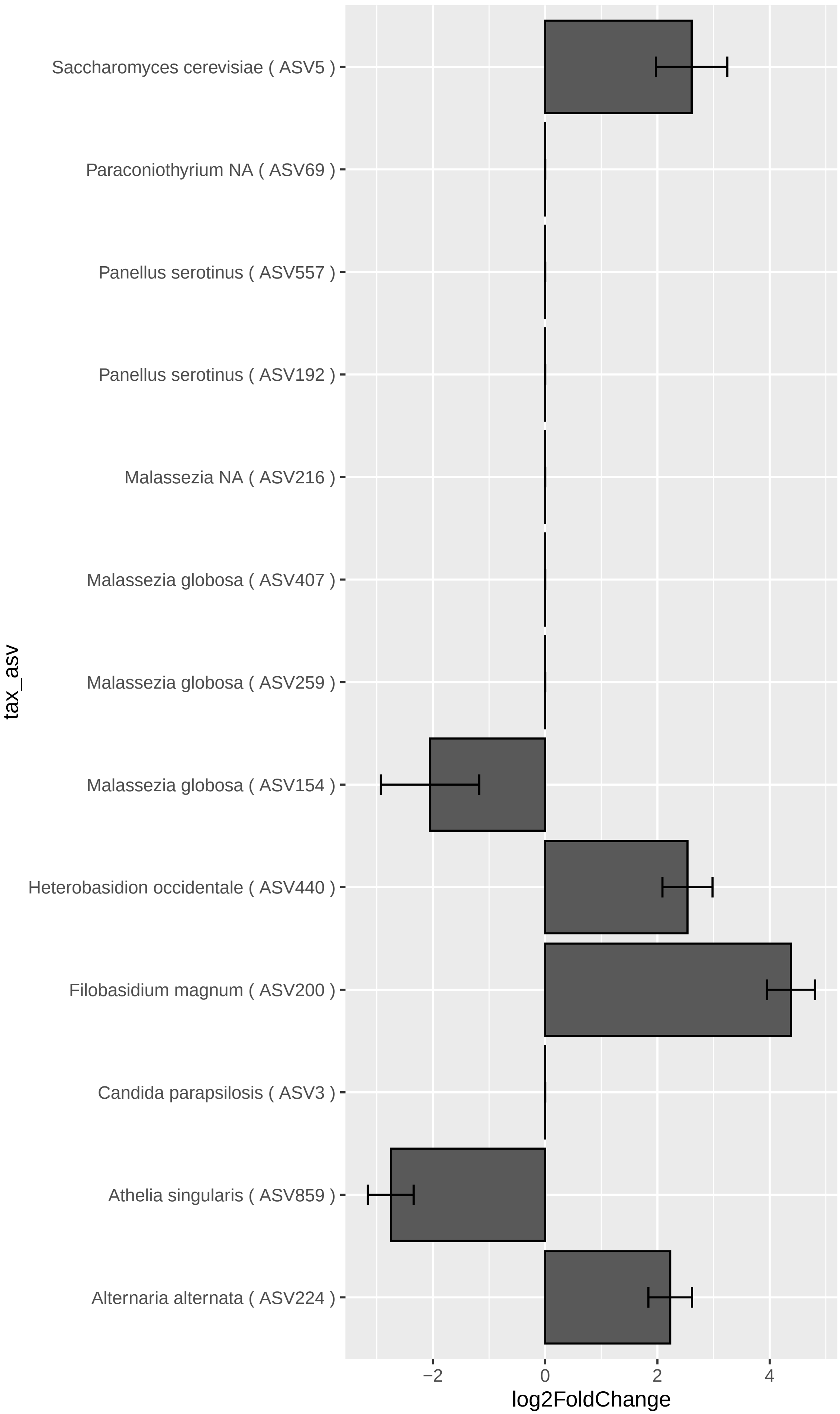

Saccharomyces cerevisiae (ASV5)
Paraconiothyrium NA (ASV69)
Panellus serotinus (ASV557)
Panellus serotinus (ASV192)
Malassezia NA (ASV216)
Malassezia globosa (ASV407)
Malassezia globosa (ASV259)
Malassezia globosa (ASV154)
Heterobasidion occidentale (ASV440)
Filobasidium magnum (ASV200)
Candida parapsilosis (ASV3)
Athelia singularis (ASV859)
Alternaria alternata (ASV224)
tax_asv
−2
0
2
4
log2FoldChange

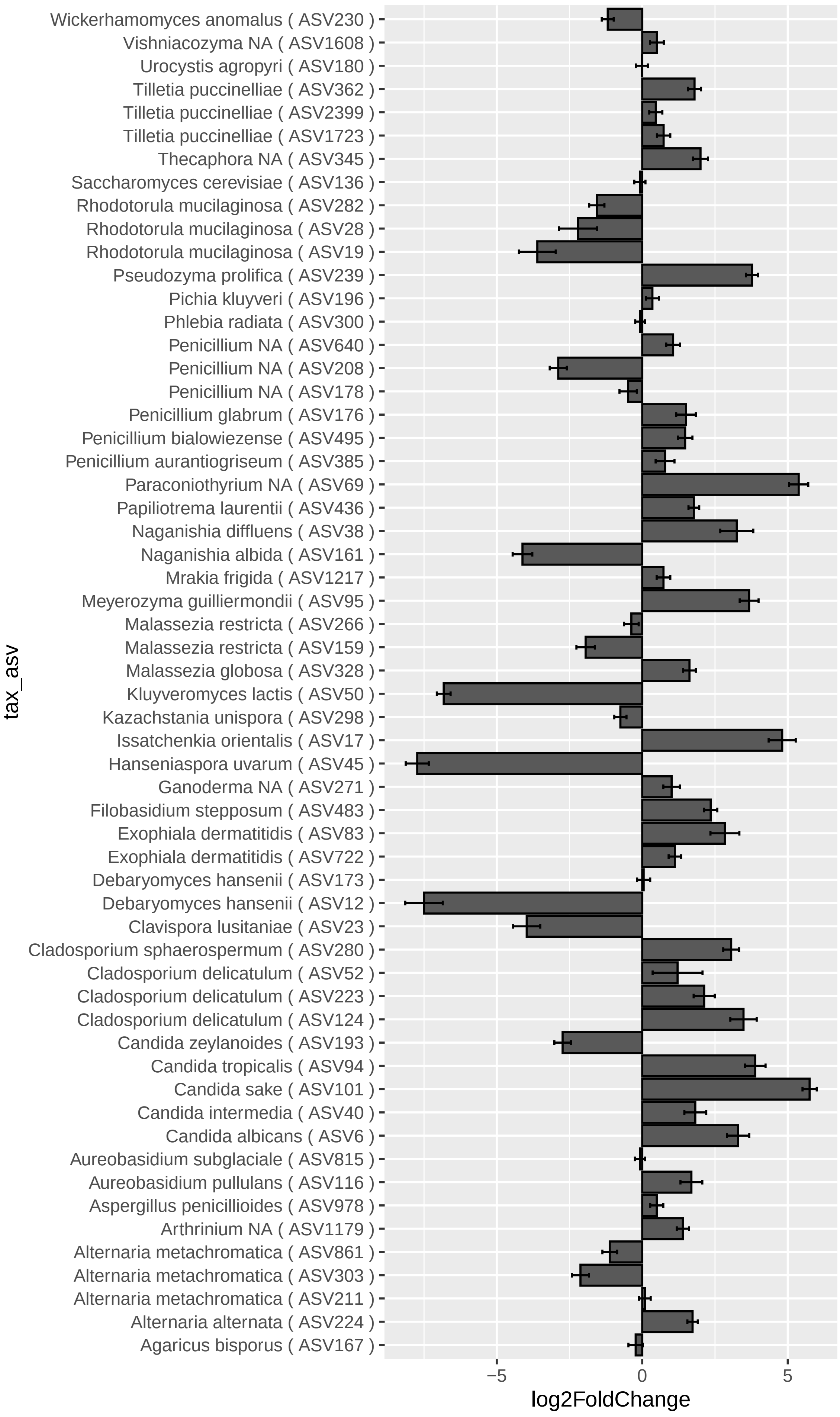

Wickerhamomyces anomalus (ASV230)
Vishniacozyma NA (ASV1608)
Urocystis agropyri (ASV180)
Tilletia puccinelliae (ASV362)
Tilletia puccinelliae (ASV2399)
Tilletia puccinelliae (ASV1723)
Thecaphora NA (ASV345)
Saccharomyces cerevisiae (ASV136)
Rhodotorula mucilaginosa (ASV282)
Rhodotorula mucilaginosa (ASV28)
Rhodotorula mucilaginosa (ASV19)
Pseudozyma prolifica (ASV239)
Pichia kluyveri (ASV196)
Phlebia radiata (ASV300)
Penicillium NA (ASV640)
Penicillium NA (ASV208)
Penicillium NA (ASV178)
Penicillium glabrum (ASV176)
Penicillium bialowiezense (ASV495)
Penicillium aurantiogriseum (ASV385)
Paraconiothyrium NA (ASV69)
Papiliotrema laurentii (ASV436)
Naganishia diffluens (ASV38)
Naganishia albida (ASV161)
Mrakia frigida (ASV1217)
Meyerozyma guilliermondii (ASV95)
Malassezia restricta (ASV266)
Malassezia restricta (ASV159)
Malassezia globosa (ASV328)
Kluyveromyces lactis (ASV50)
Kazachstania unispora (ASV298)
Issatchenkia orientalis (ASV17)
Hanseniaspora uvarum (ASV45)
Ganoderma NA (ASV271)
Filobasidium stepposum (ASV483)
Exophiala dermatitidis (ASV83)
Exophiala dermatitidis (ASV722)
Debaryomyces hansenii (ASV173)
Debaryomyces hansenii (ASV12)
Clavispora lusitaniae (ASV23)
Cladosporium sphaerospermum (ASV280)
Cladosporium delicatulum (ASV52)
Cladosporium delicatulum (ASV223)
Cladosporium delicatulum (ASV124)
Candida zeylanoides (ASV193)
Candida tropicalis (ASV94)
Candida sake (ASV101)
Candida intermedia (ASV40)
Candida albicans (ASV6)
Aureobasidium subglaciale (ASV815)
Aureobasidium pullulans (ASV116)
Aspergillus penicillioides (ASV978)
Arthrinium NA (ASV1179)
Alternaria metachromatica (ASV861)
Alternaria metachromatica (ASV303)
Alternaria metachromatica (ASV211)
Alternaria alternata (ASV224)
Agaricus bisporus (ASV167)
tax_asv
−5
0
5
log2FoldChange

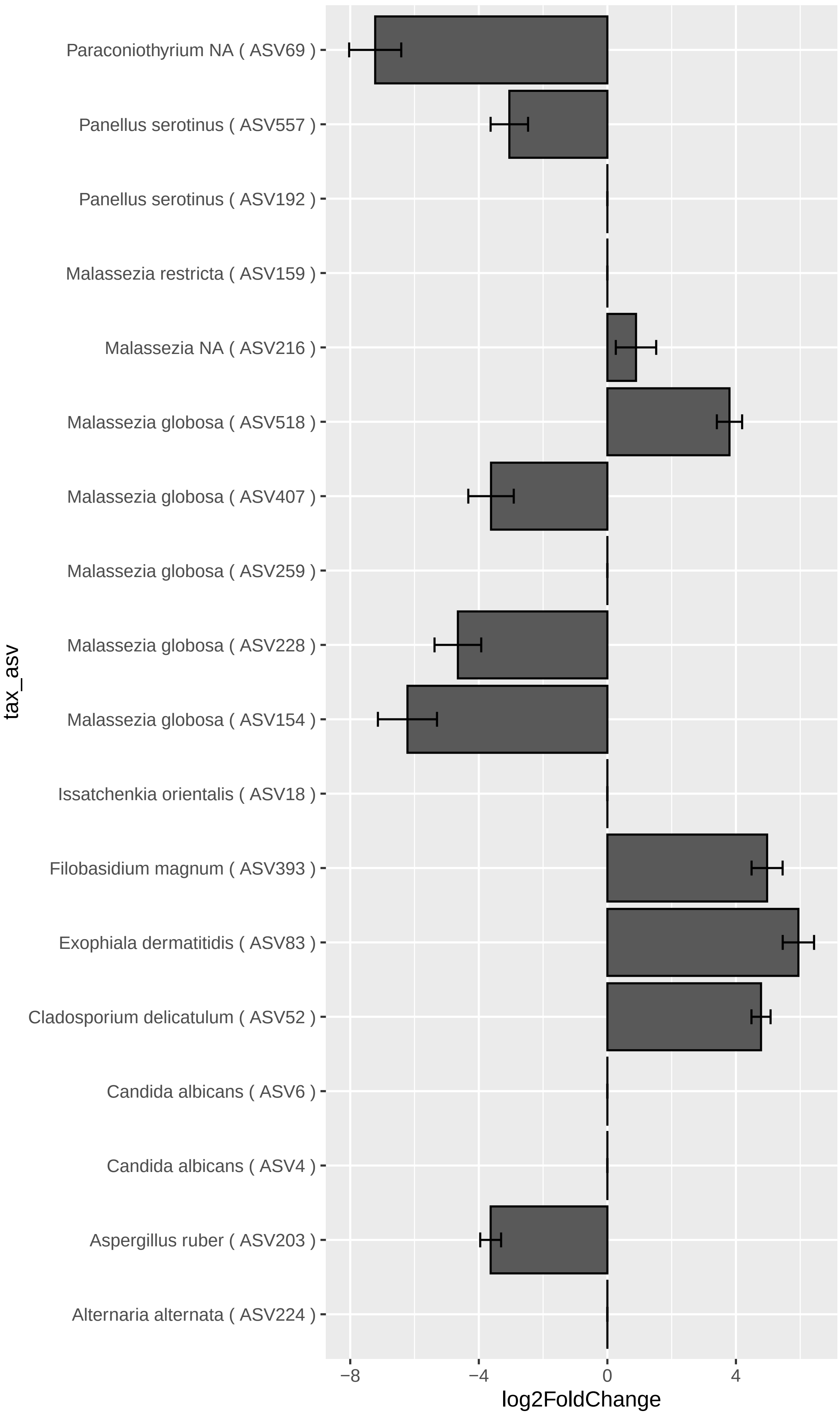

Paraconiothyrium NA (ASV69)
Panellus serotinus (ASV557)
Panellus serotinus (ASV192)
Malassezia restricta (ASV159)
Malassezia NA (ASV216)
Malassezia globosa (ASV518)
Malassezia globosa (ASV407)
Malassezia globosa (ASV259)
Malassezia globosa (ASV228)
Malassezia globosa (ASV154)
Issatchenkia orientalis (ASV18)
Filobasidium magnum (ASV393)
Exophiala dermatitidis (ASV83)
Cladosporium delicatulum (ASV52)
Candida albicans (ASV6)
Candida albicans (ASV4)
Aspergillus ruber (ASV203)
Alternaria alternata (ASV224)
tax_asv
−8
−4
0
4
log2FoldChange

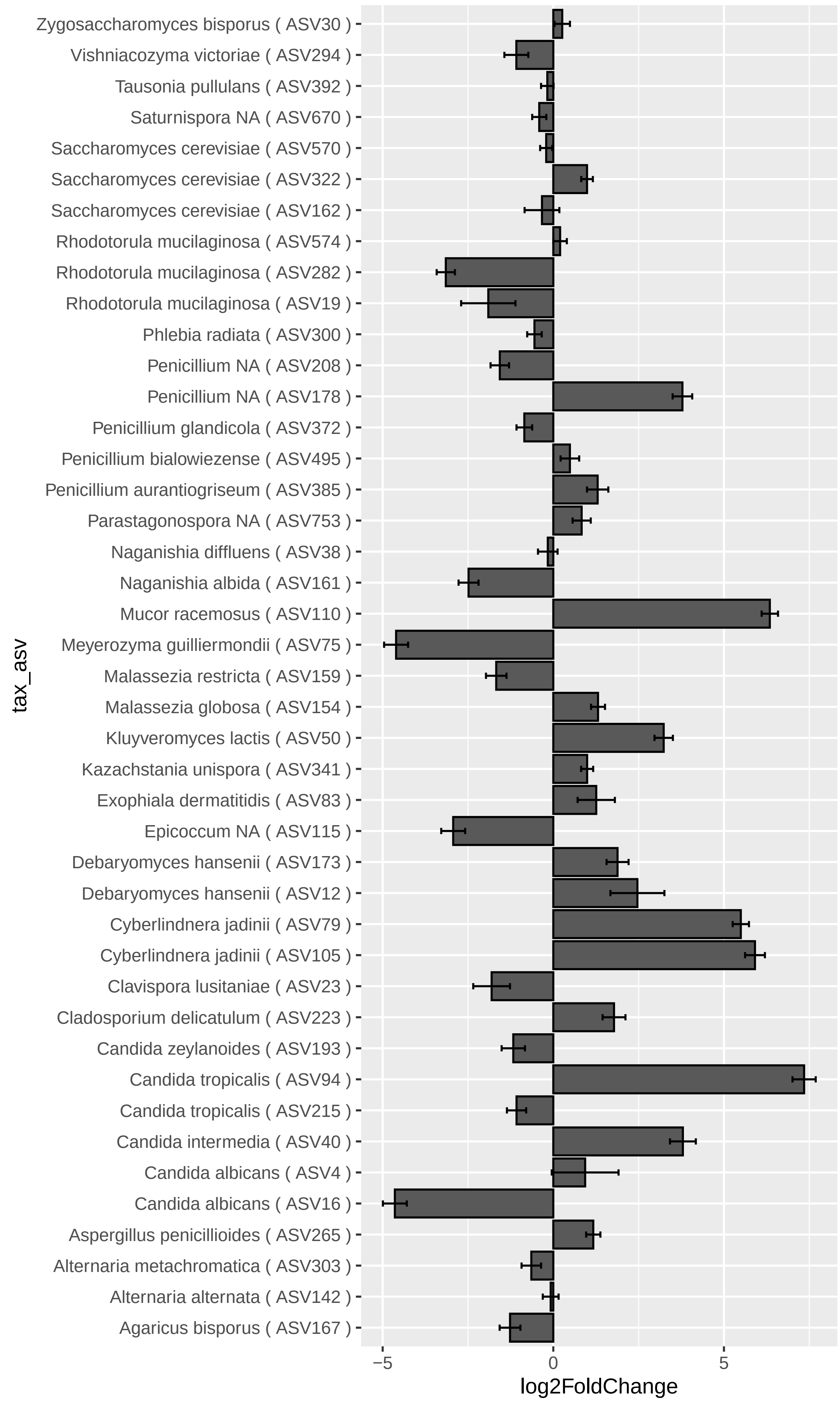
Zygosaccharomyces bisporus (ASV30)
Vishniacozyma victoriae (ASV294)
Tausonia pullulans (ASV392)
Saturnispora NA (ASV670)
Saccharomyces cerevisiae (ASV570)
Saccharomyces cerevisiae (ASV322)
Saccharomyces cerevisiae (ASV162)
Rhodotorula mucilaginosa (ASV574)
Rhodotorula mucilaginosa (ASV282)
Rhodotorula mucilaginosa (ASV19)
Phlebia radiata (ASV300)
Penicillium NA (ASV208)
Penicillium NA (ASV178)
Penicillium glandicola (ASV372)
Penicillium bialowiezense (ASV495)
Penicillium aurantiogriseum (ASV385)
Parastagonospora NA (ASV753)
Naganishia diffluens (ASV38)
Naganishia albida (ASV161)
Mucor racemosus (ASV110)
Meyerozyma guilliermondii (ASV75)
Malassezia restricta (ASV159)
Malassezia globosa (ASV154)
Kluyveromyces lactis (ASV50)
Kazachstania unispora (ASV341)
Exophiala dermatitidis (ASV83)
Epicoccum NA (ASV115)
Debaryomyces hansenii (ASV173)
Debaryomyces hansenii (ASV12)
Cyberlindnera jadinii (ASV79)
Cyberlindnera jadinii (ASV105)
Clavispora lusitaniae (ASV23)
Cladosporium delicatulum (ASV223)
Candida zeylanoides (ASV193)
Candida tropicalis (ASV94)
Candida tropicalis (ASV215)
Candida intermedia (ASV40)
Candida albicans (ASV4)
Candida albicans (ASV16)
Aspergillus penicillioides (ASV265)
Alternaria metachromatica (ASV303)
Alternaria alternata (ASV142)
Agaricus bisporus (ASV167)
tax_asv
−5
0
5
log2FoldChange

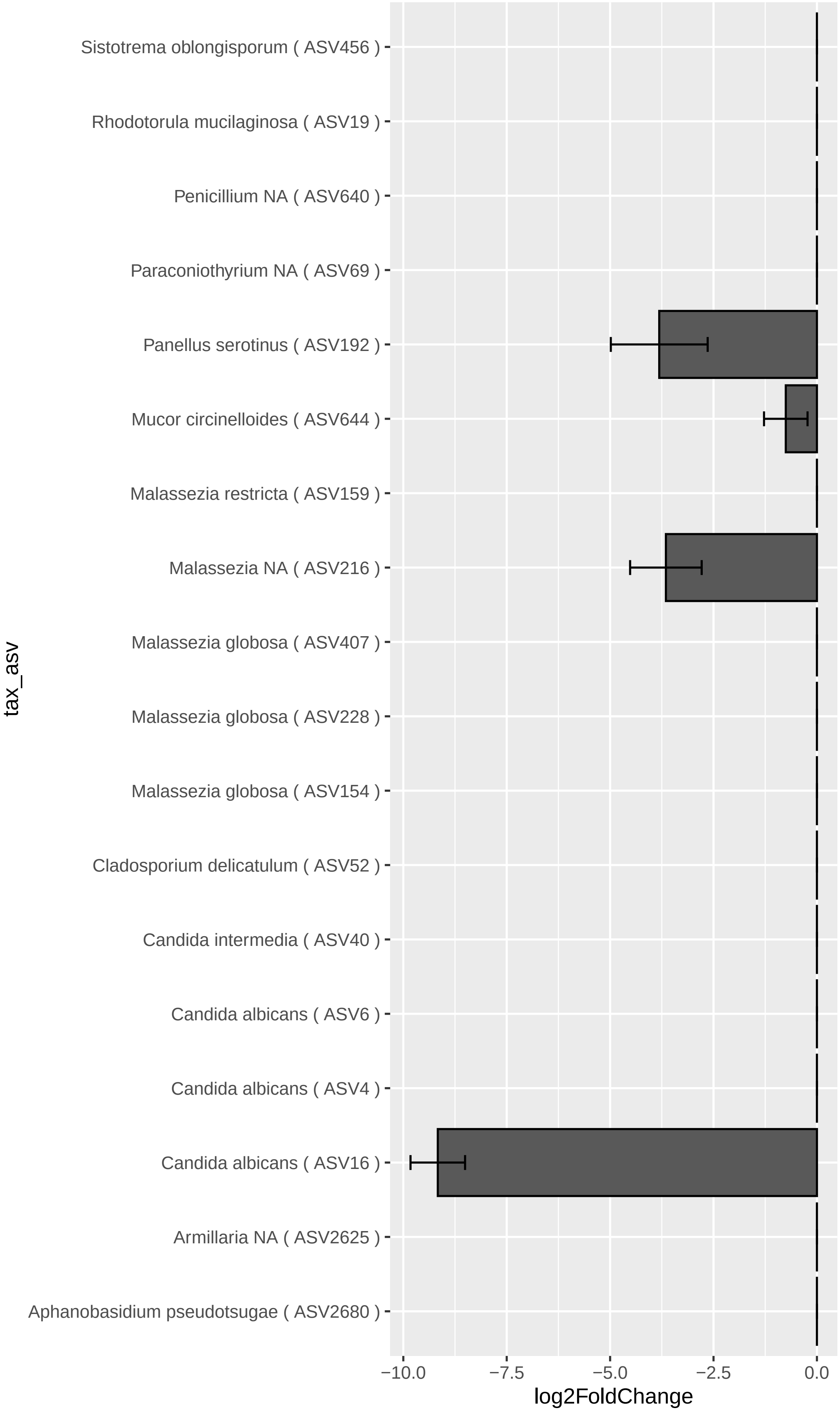
Sistotrema oblongisporum (ASV456)
Rhodotorula mucilaginosa (ASV19)
Penicillium NA (ASV640)
Paraconiothyrium NA (ASV69)
Panellus serotinus (ASV192)
Mucor circinelloides (ASV644)
Malassezia restricta (ASV159)
Malassezia NA (ASV216)
Malassezia globosa (ASV407)
Malassezia globosa (ASV228)
Malassezia globosa (ASV154)
Cladosporium delicatulum (ASV52)
Candida intermedia (ASV40)
Candida albicans (ASV6)
Candida albicans (ASV4)
Candida albicans (ASV16)
Armillaria NA (ASV2625)
Aphanobasidium pseudotsugae (ASV2680)
tax_asv
−10.0
−7.5
−5.0
−2.5
0.0
log2FoldChange

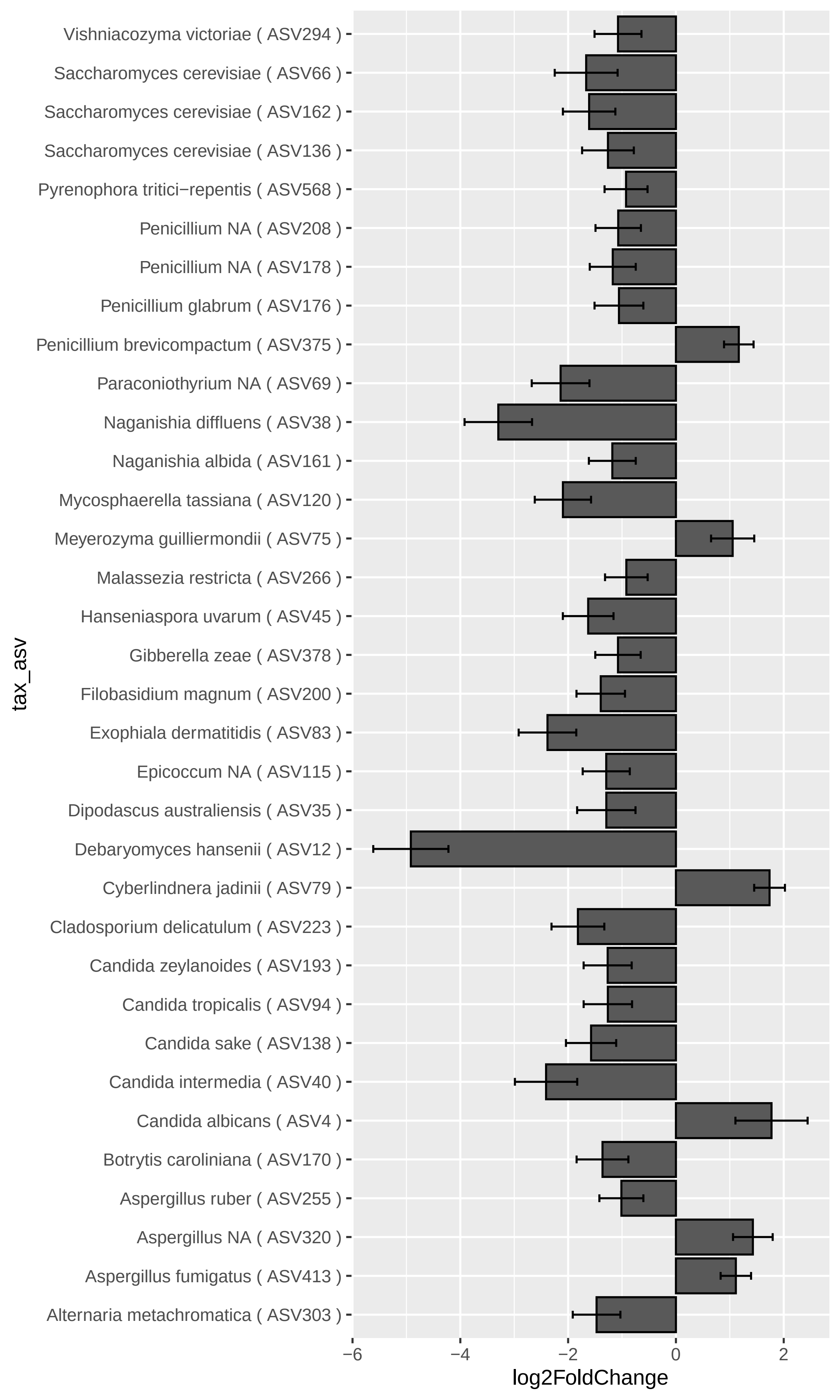

Vishniacozyma victoriae (ASV294)
Saccharomyces cerevisiae (ASV66)
Saccharomyces cerevisiae (ASV162)
Saccharomyces cerevisiae (ASV136)
Pyrenophora tritici−repentis (ASV568)
Penicillium NA (ASV208)
Penicillium NA (ASV178)
Penicillium glabrum (ASV176)
Penicillium brevicompactum (ASV375)
Paraconiothyrium NA (ASV69)
Naganishia diffluens (ASV38)
Naganishia albida (ASV161)
Mycosphaerella tassiana (ASV120)
Meyerozyma guilliermondii (ASV75)
Malassezia restricta (ASV266)
Hanseniaspora uvarum (ASV45)
Gibberella zeae (ASV378)
Filobasidium magnum (ASV200)
Exophiala dermatitidis (ASV83)
Epicoccum NA (ASV115)
Dipodascus australiensis (ASV35)
Debaryomyces hansenii (ASV12)
Cyberlindnera jadinii (ASV79)
Cladosporium delicatulum (ASV223)
Candida zeylanoides (ASV193)
Candida tropicalis (ASV94)
Candida sake (ASV138)
Candida intermedia (ASV40)
Candida albicans (ASV4)
Botrytis caroliniana (ASV170)
Aspergillus ruber (ASV255)
Aspergillus NA (ASV320)
Aspergillus fumigatus (ASV413)
Alternaria metachromatica (ASV303)
tax_asv
−6
−4
−2
0
2
log2FoldChange

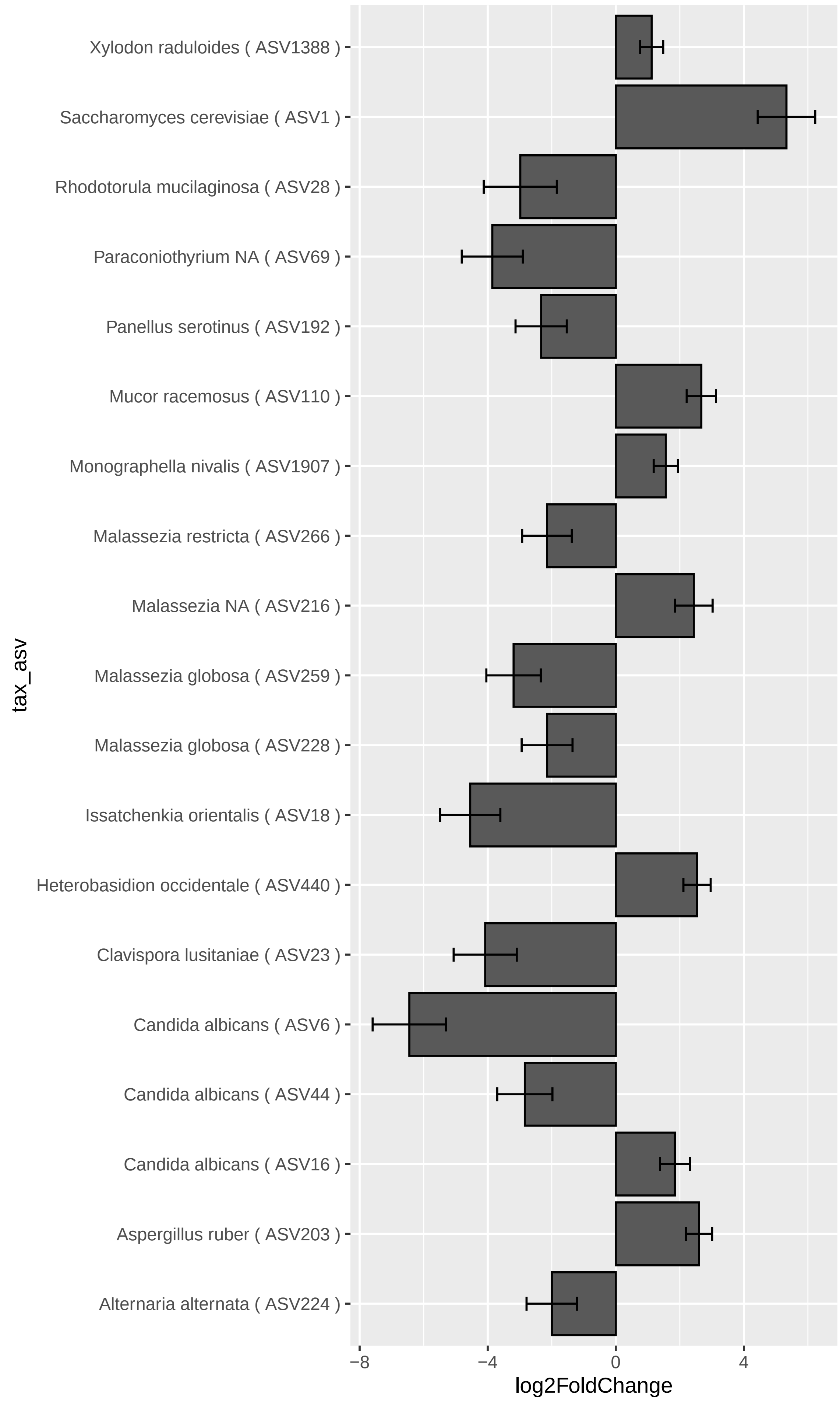
Xylodon raduloides (ASV1388)
Saccharomyces cerevisiae (ASV1)
Rhodotorula mucilaginosa (ASV28)
Paraconiothyrium NA (ASV69)
Panellus serotinus (ASV192)
Mucor racemosus (ASV110)
Monographella nivalis (ASV1907)
Malassezia restricta (ASV266)
Malassezia NA (ASV216)
Malassezia globosa (ASV259)
Malassezia globosa (ASV228)
Issatchenkia orientalis (ASV18)
Heterobasidion occidentale (ASV440)
Clavispora lusitaniae (ASV23)
Candida albicans (ASV6)
Candida albicans (ASV44)
Candida albicans (ASV16)
Aspergillus ruber (ASV203)
Alternaria alternata (ASV224)
tax_asv
−8
−4
0
4
log2FoldChange

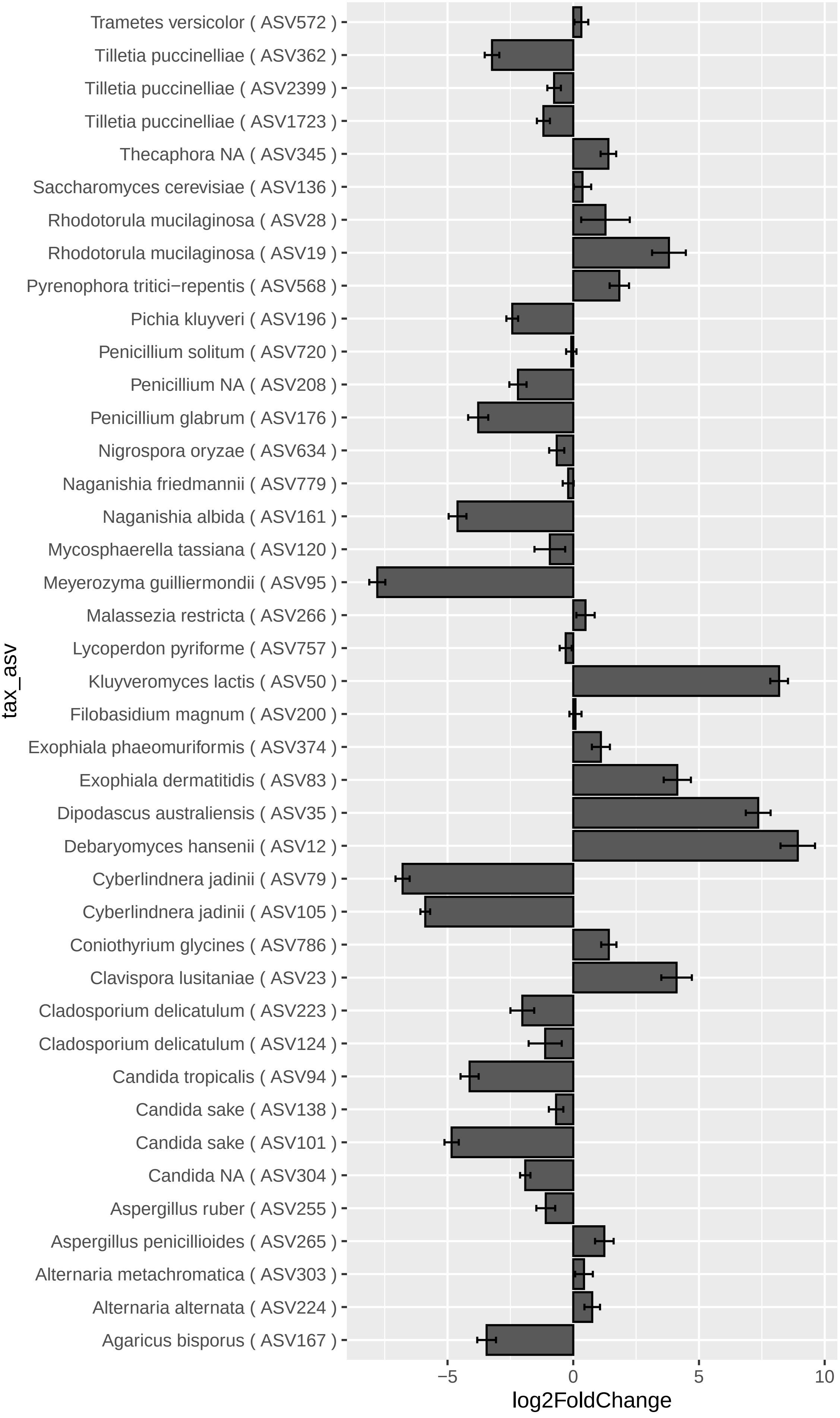

Trametes versicolor (ASV572)
Tilletia puccinelliae (ASV362)
Tilletia puccinelliae (ASV2399)
Tilletia puccinelliae (ASV1723)
Thecaphora NA (ASV345)
Saccharomyces cerevisiae (ASV136)
Rhodotorula mucilaginosa (ASV28)
Rhodotorula mucilaginosa (ASV19)
Pyrenophora tritici−repentis (ASV568)
Pichia kluyveri (ASV196)
Penicillium solitum (ASV720)
Penicillium NA (ASV208)
Penicillium glabrum (ASV176)
Nigrospora oryzae (ASV634)
Naganishia friedmannii (ASV779)
Naganishia albida (ASV161)
Mycosphaerella tassiana (ASV120)
Meyerozyma guilliermondii (ASV95)
Malassezia restricta (ASV266)
Lycoperdon pyriforme (ASV757)
Kluyveromyces lactis (ASV50)
Filobasidium magnum (ASV200)
Exophiala phaeomuriformis (ASV374)
Exophiala dermatitidis (ASV83)
Dipodascus australiensis (ASV35)
Debaryomyces hansenii (ASV12)
Cyberlindnera jadinii (ASV79)
Cyberlindnera jadinii (ASV105)
Coniothyrium glycines (ASV786)
Clavispora lusitaniae (ASV23)
Cladosporium delicatulum (ASV223)
Cladosporium delicatulum (ASV124)
Candida tropicalis (ASV94)
Candida sake (ASV138)
Candida sake (ASV101)
Candida NA (ASV304)
Aspergillus ruber (ASV255)
Aspergillus penicillioides (ASV265)
Alternaria metachromatica (ASV303)
Alternaria alternata (ASV224)
Agaricus bisporus (ASV167)
tax_asv
−5
0
5
10
log2FoldChange

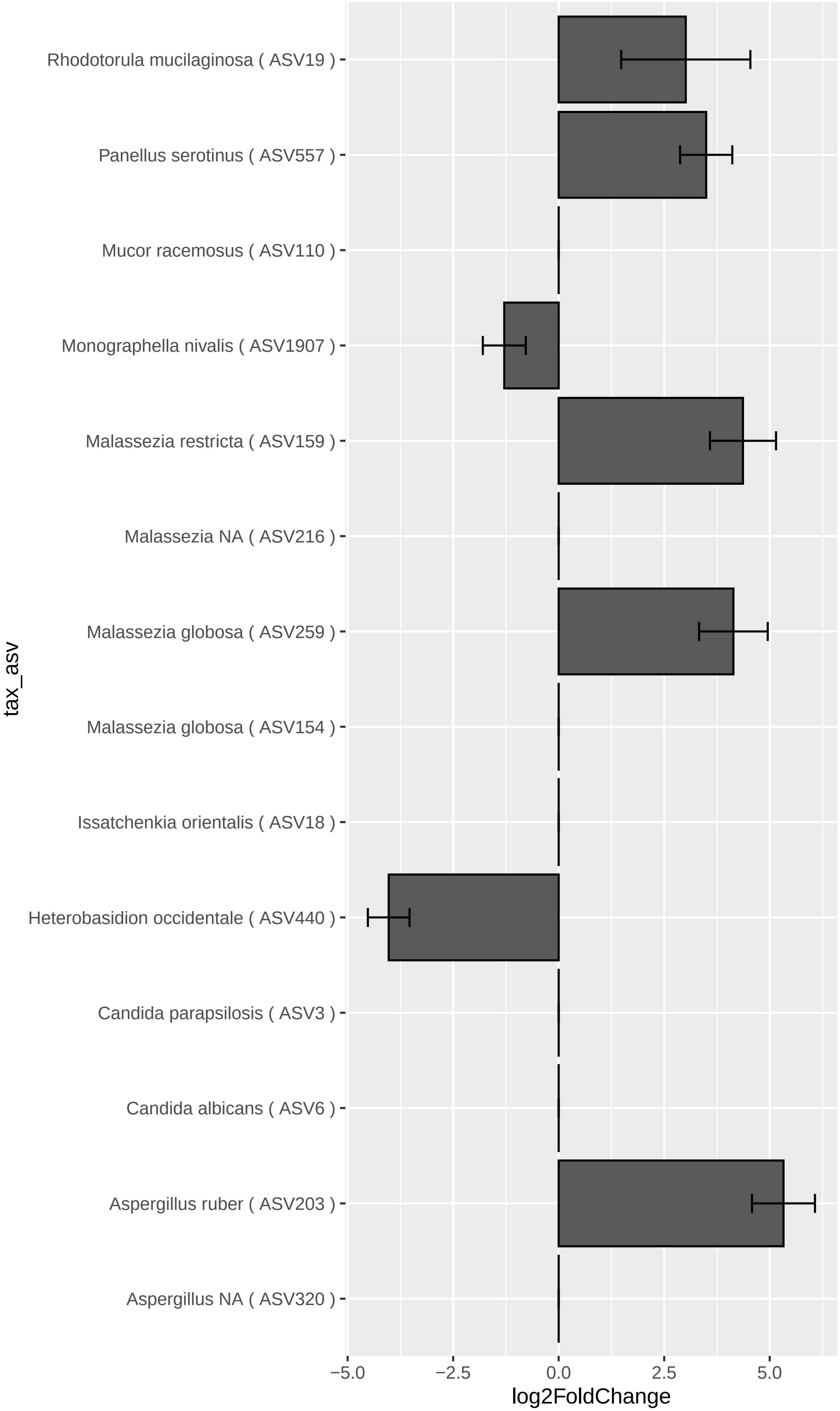
Rhodotorula mucilaginosa (ASV19)
Panellus serotinus (ASV557)
Mucor racemosus (ASV110)
Monographella nivalis (ASV1907)
Malassezia restricta (ASV159)
Malassezia NA (ASV216)
Malassezia globosa (ASV259)
Malassezia globosa (ASV154)
Issatchenkia orientalis (ASV18)
Heterobasidion occidentale (ASV440)
Candida parapsilosis (ASV3)
Candida albicans (ASV6)
Aspergillus ruber (ASV203)
Aspergillus NA (ASV320)
tax_asv
−5.0
−2.5
0.0
2.5
5.0
log2FoldChange

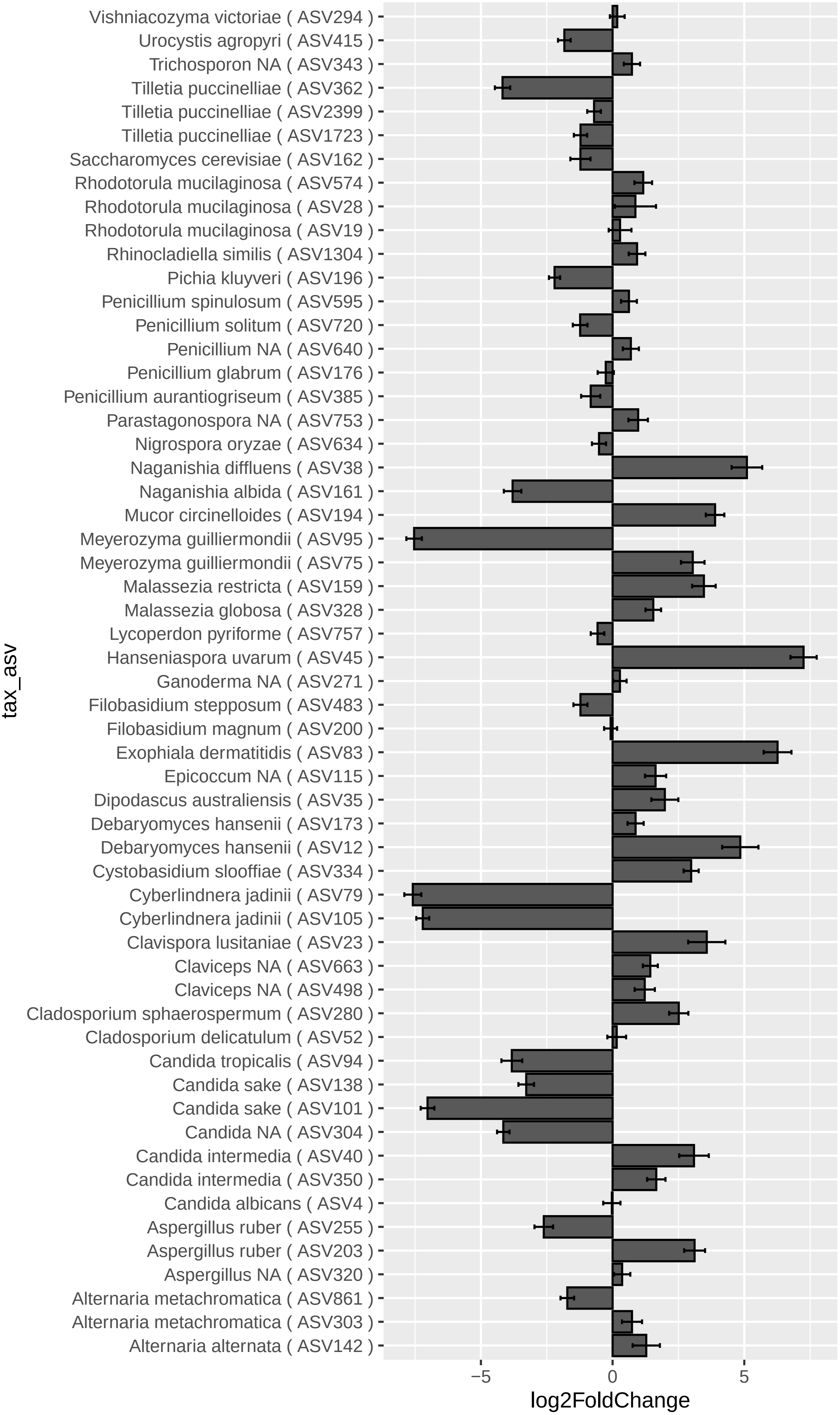

Vishniacozyma victoriae (ASV294)
Urocystis agropyri (ASV415)
Trichosporon NA (ASV343)
Tilletia puccinelliae (ASV362)
Tilletia puccinelliae (ASV2399)
Tilletia puccinelliae (ASV1723)
Saccharomyces cerevisiae (ASV162)
Rhodotorula mucilaginosa (ASV574)
Rhodotorula mucilaginosa (ASV28)
Rhodotorula mucilaginosa (ASV19)
Rhinocladiella similis (ASV1304)
Pichia kluyveri (ASV196)
Penicillium spinulosum (ASV595)
Penicillium solitum (ASV720)
Penicillium NA (ASV640)
Penicillium glabrum (ASV176)
Penicillium aurantiogriseum (ASV385)
Parastagonospora NA (ASV753)
Nigrospora oryzae (ASV634)
Naganishia diffluens (ASV38)
Naganishia albida (ASV161)
Mucor circinelloides (ASV194)
Meyerozyma guilliermondii (ASV95)
Meyerozyma guilliermondii (ASV75)
Malassezia restricta (ASV159)
Malassezia globosa (ASV328)
Lycoperdon pyriforme (ASV757)
Hanseniaspora uvarum (ASV45)
Ganoderma NA (ASV271)
Filobasidium stepposum (ASV483)
Filobasidium magnum (ASV200)
Exophiala dermatitidis (ASV83)
Epicoccum NA (ASV115)
Dipodascus australiensis (ASV35)
Debaryomyces hansenii (ASV173)
Debaryomyces hansenii (ASV12)
Cystobasidium slooffiae (ASV334)
Cyberlindnera jadinii (ASV79)
Cyberlindnera jadinii (ASV105)
Clavispora lusitaniae (ASV23)
Claviceps NA (ASV663)
Claviceps NA (ASV498)
Cladosporium sphaerospermum (ASV280)
Cladosporium delicatulum (ASV52)
Candida tropicalis (ASV94)
Candida sake (ASV138)
Candida sake (ASV101)
Candida NA (ASV304)
Candida intermedia (ASV40)
Candida intermedia (ASV350)
Candida albicans (ASV4)
Aspergillus ruber (ASV255)
Aspergillus ruber (ASV203)
Aspergillus NA (ASV320)
Alternaria metachromatica (ASV861)
Alternaria metachromatica (ASV303)
Alternaria alternata (ASV142)
tax_asv
−5
0
5
log2FoldChange

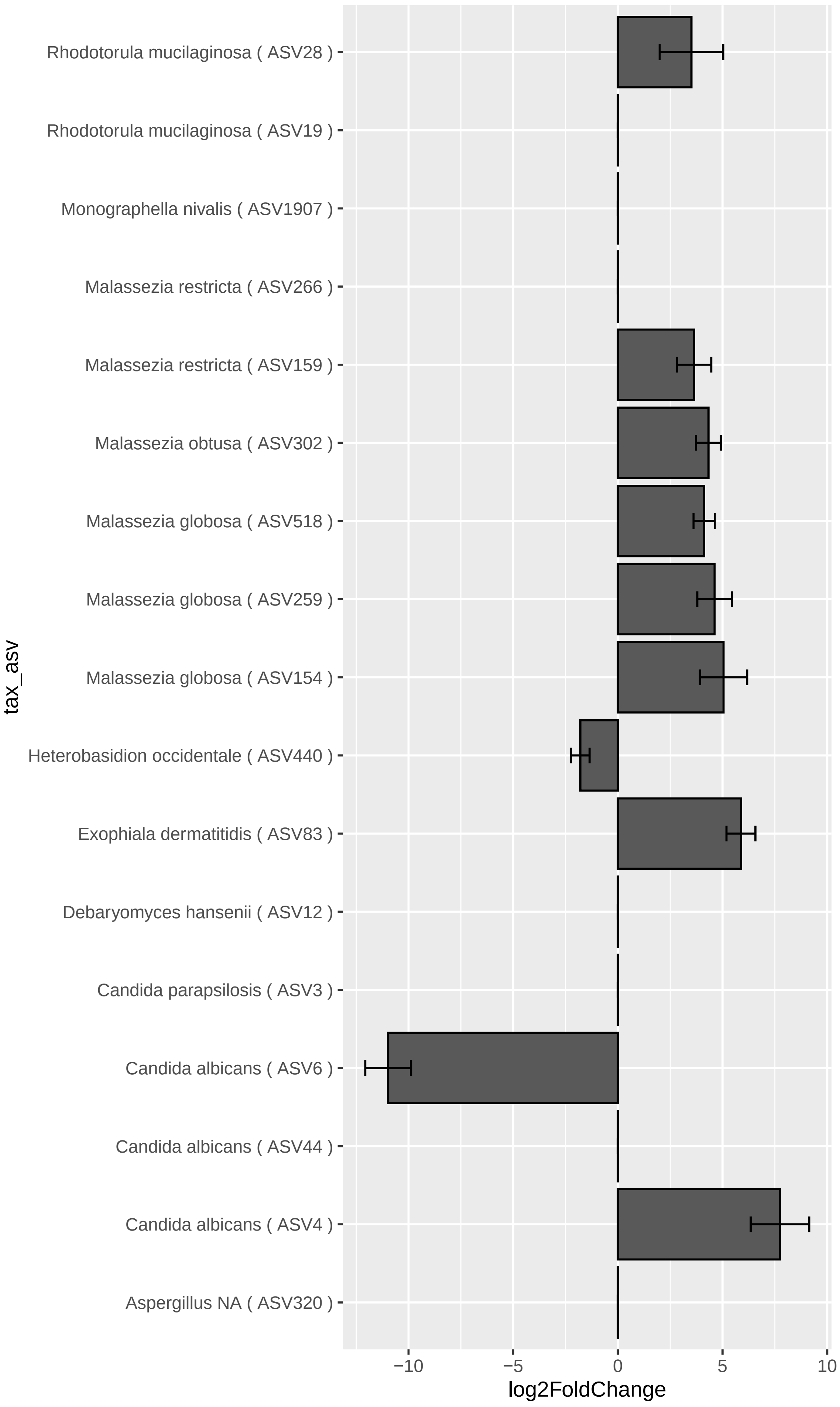

Rhodotorula mucilaginosa (ASV28)
Rhodotorula mucilaginosa (ASV19)
Monographella nivalis (ASV1907)
Malassezia restricta (ASV266)
Malassezia restricta (ASV159)
Malassezia obtusa (ASV302)
Malassezia globosa (ASV518)
Malassezia globosa (ASV259)
Malassezia globosa (ASV154)
Heterobasidion occidentale (ASV440)
Exophiala dermatitidis (ASV83)
Debaryomyces hansenii (ASV12)
Candida parapsilosis (ASV3)
Candida albicans (ASV6)
Candida albicans (ASV44)
Candida albicans (ASV4)
Aspergillus NA (ASV320)
tax_asv
−10
−5
0
5
10
log2FoldChange

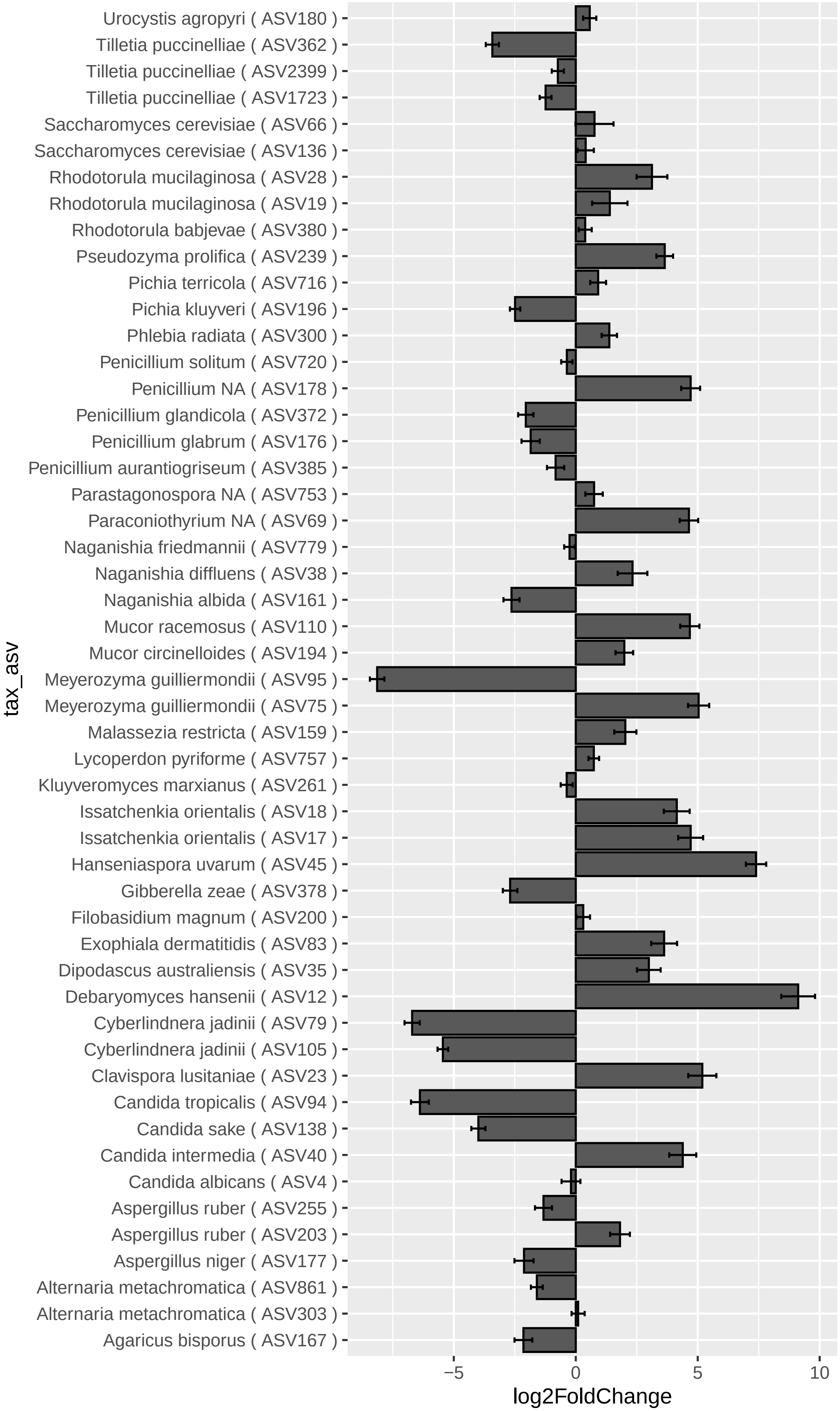

Urocystis agropyri (ASV180)
Tilletia puccinelliae (ASV362)
Tilletia puccinelliae (ASV2399)
Tilletia puccinelliae (ASV1723)
Saccharomyces cerevisiae (ASV66)
Saccharomyces cerevisiae (ASV136)
Rhodotorula mucilaginosa (ASV28)
Rhodotorula mucilaginosa (ASV19)
Rhodotorula babjevae (ASV380)
Pseudozyma prolifica (ASV239)
Pichia terricola (ASV716)
Pichia kluyveri (ASV196)
Phlebia radiata (ASV300)
Penicillium solitum (ASV720)
Penicillium NA (ASV178)
Penicillium glandicola (ASV372)
Penicillium glabrum (ASV176)
Penicillium aurantiogriseum (ASV385)
Parastagonospora NA (ASV753)
Paraconiothyrium NA (ASV69)
Naganishia friedmannii (ASV779)
Naganishia diffluens (ASV38)
Naganishia albida (ASV161)
Mucor racemosus (ASV110)
Mucor circinelloides (ASV194)
Meyerozyma guilliermondii (ASV95)
Meyerozyma guilliermondii (ASV75)
Malassezia restricta (ASV159)
Lycoperdon pyriforme (ASV757)
Kluyveromyces marxianus (ASV261)
Issatchenkia orientalis (ASV18)
Issatchenkia orientalis (ASV17)
Hanseniaspora uvarum (ASV45)
Gibberella zeae (ASV378)
Filobasidium magnum (ASV200)
Exophiala dermatitidis (ASV83)
Dipodascus australiensis (ASV35)
Debaryomyces hansenii (ASV12)
Cyberlindnera jadinii (ASV79)
Cyberlindnera jadinii (ASV105)
Clavispora lusitaniae (ASV23)
Candida tropicalis (ASV94)
Candida sake (ASV138)
Candida intermedia (ASV40)
Candida albicans (ASV4)
Aspergillus ruber (ASV255)
Aspergillus ruber (ASV203)
Aspergillus niger (ASV177)
Alternaria metachromatica (ASV861)
Alternaria metachromatica (ASV303)
Agaricus bisporus (ASV167)
tax_asv
−5
0
5
10
log2FoldChange

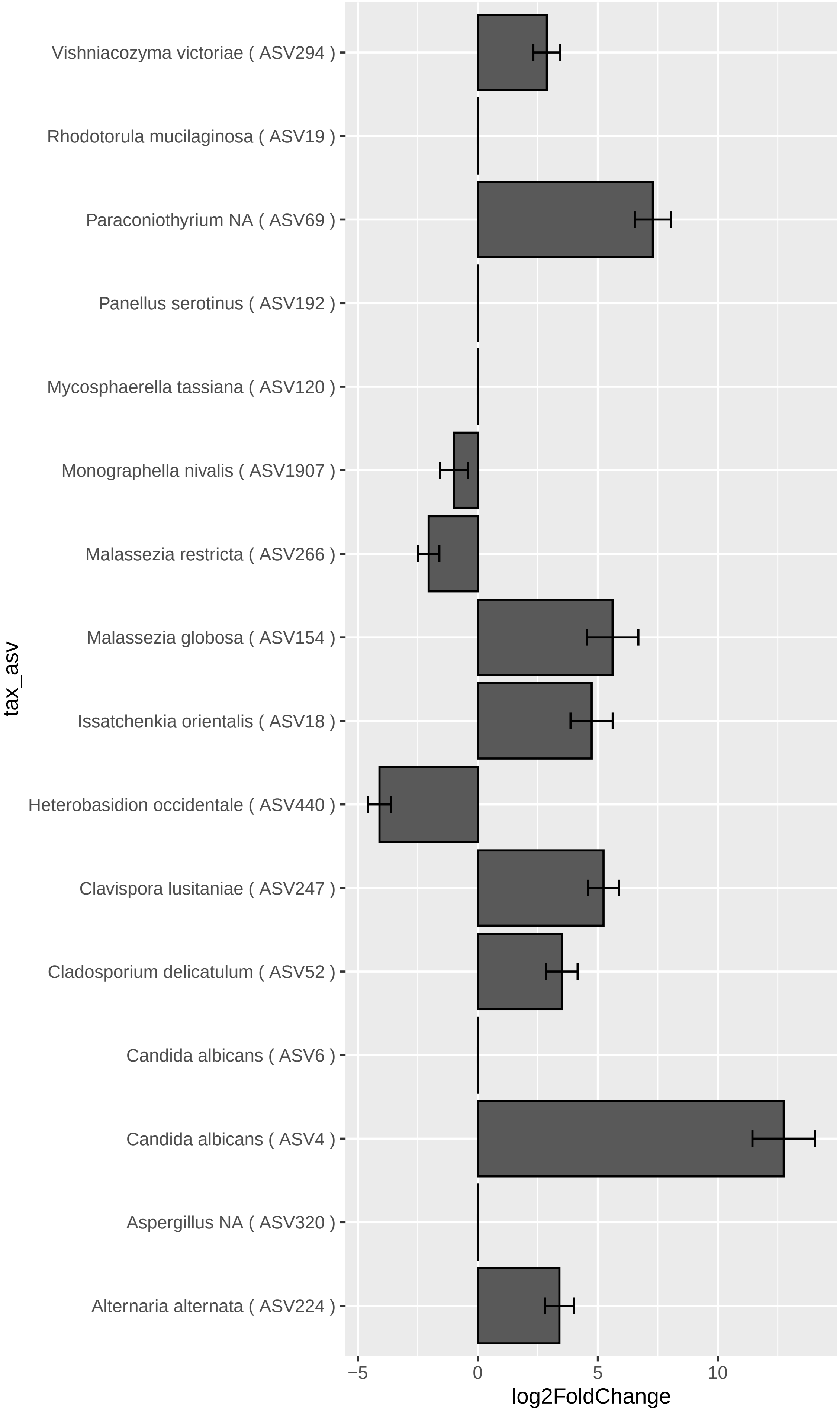

Vishniacozyma victoriae (ASV294)
Rhodotorula mucilaginosa (ASV19)
Paraconiothyrium NA (ASV69)
Panellus serotinus (ASV192)
Mycosphaerella tassiana (ASV120)
Monographella nivalis (ASV1907)
Malassezia restricta (ASV266)
Malassezia globosa (ASV154)
Issatchenkia orientalis (ASV18)
Heterobasidion occidentale (ASV440)
Clavispora lusitaniae (ASV247)
Cladosporium delicatulum (ASV52)
Candida albicans (ASV6)
Candida albicans (ASV4)
Aspergillus NA (ASV320)
Alternaria alternata (ASV224)
tax_asv
−5
0
5
10
log2FoldChange

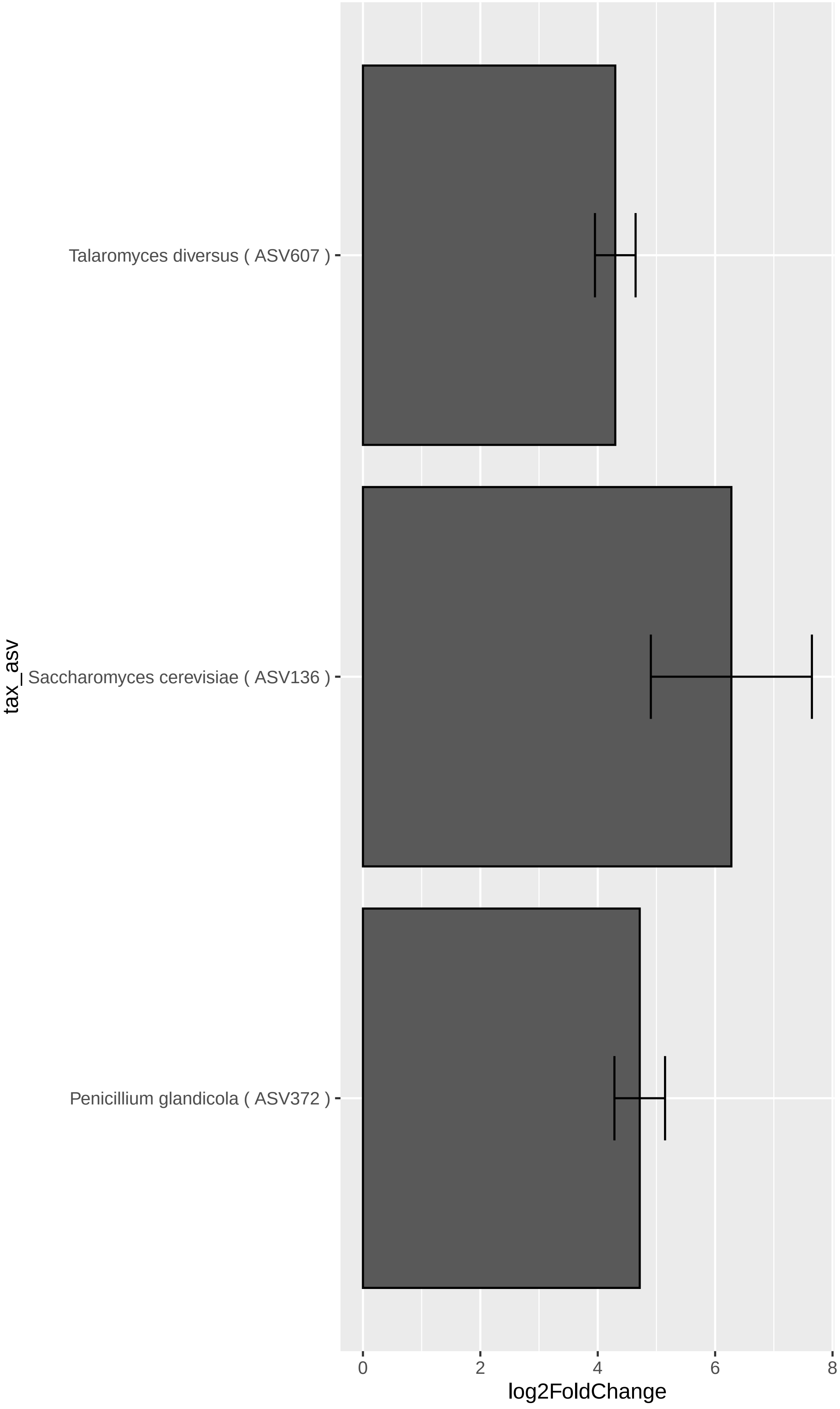
Talaromyces diversus (ASV607)
Saccharomyces cerevisiae (ASV136)
Penicillium glandicola (ASV372)
tax_asv
0
2
4
6
8
log2FoldChange
