# Supplementary material for: Composition and Associations of the Infant Gut Fungal Microbiota with Environmental Factors and Childhood Allergic Outcomes
Source: mBio. 2021 Jun 1;12(3):e03396-20. doi: 10.1128/mBio.03396-20 (PMC8263004; doi:10.1128/mBio.03396-20)
Supplement: TABLE S2 [file mbio.03396-20-st002.docx]

Table S2A. Demographic and clinical characteristics of CHILD cohort subjects with and without inhalant atopy (inhalant allergen sensitization) at age five years with available three-month stool ITS-2 rDNA sequencing data. Wilcoxon rank sum test and Fisher's exact test were used for continuous and categorical variables, respectively.

| **Variable** | **Cohort** | **Inhalant atopy** | **No inhalant atopy** | **P-value** |
| --- | --- | --- | --- | --- |
| No. patients | 81 | 14 | 56 |  |
| Institution, n(%) |  |  |  | 0.02 |
| Edmonton | 14 (17.3%) | 4 (28.6%) | 7 (12.5%) |  |
| Toronto | 24 (29.6%) | 6 (42.9%) | 16 (28.6%) |  |
| Vancouver | 19 (23.5%) | 4 (28.6%) | 13 (23.2%) |  |
| Winnipeg | 24 (29.6%) |  | 20 (35.7%) |  |
| Antibiotics use by age 3 months, n(%) |  |  |  | 1 |
|  | 2 (2.5%) | 0 (0%) | 1 (1.8%) |  |
| Unknown | 1 (1.2%) |  | 1 (1.8%) |  |
| Mode of Delivery, n(%) |  |  |  | 0.02 |
| C-Section with labour | 12 (14.8%) | 5 (35.7%) | 5 (8.9%) |  |
| C-Section without labour | 8 (9.9%) | 2 (14.3%) | 5 (8.9%) |  |
| Vaginal | 60 (74.1%) | 7 (50%) | 45 (80.4%) |  |
| Unknown | 1 (1.2%) |  | 1 (1.8%) |  |
| Having Older Sibling, n(%) |  |  |  | 0.56 |
|  | 38 (46.9%) | 8 (57.1%) | 26 (46.4%) |  |
| Unknown | 1 (1.2%) |  | 1 (1.8%) |  |
| Season of Birth, n(%) |  |  |  | 0.29 |
| Spring | 29 (35.8%) | 3 (21.4%) | 23 (41.1%) |  |
| Summer | 24 (29.6%) | 3 (21.4%) | 16 (28.6%) |  |
| Fall | 17 (21%) | 5 (35.7%) | 10 (17.9%) |  |
| Winter | 11 (13.6%) | 3 (21.4%) | 7 (12.5%) |  |
| Area type (Rural), n(%) |  |  |  | 1 |
|  | 7 (8.6%) | 0 (0%) | 3 (5.4%) |  |
| Unknown | 2 (2.5%) | 1 (7.1%) | 1 (1.8%) |  |
| Breastfeeding status at age 3 months, n(%) |  |  |  | 0.67 |
|  | 67 (82.7%) | 13 (92.9%) | 46 (82.1%) |  |
| Unknown | 1 (1.2%) |  | 1 (1.8%) |  |
| Perinatal pet exposure, n(%) |  |  |  | 0.2 |
|  | 37 (45.7%) | 4 (28.6%) | 30 (53.6%) |  |
| Unknown | 7 (8.6%) | 2 (14.3%) | 4 (7.1%) |  |
| Presence of mould in home, n(%) |  |  |  | 0.22 |
|  | 32 (39.5%) | 8 (57.1%) | 20 (35.7%) |  |
| Antifungal use by age 3 months, n(%) |  |  |  | 0.68 |
|  | 9 (11.1%) | 1 (7.1%) | 8 (14.3%) |  |
| Solid food by age 3 months, n(%) |  |  |  | 1 |
|  | 11 (13.6%) | 2 (14.3%) | 6 (10.7%) |  |
| Unknown | 5 (6.2%) |  | 5 (8.9%) |  |

Table S2B. Demographic and clinical characteristics of CHILD cohort subjects with and without inhalant atopy (inhalant allergen sensitization) at age five years with available one-year stool ITS-2 rDNA sequencing data. Wilcoxon rank sum test and Fisher's exact test were used for continuous and categorical variables, respectively.

| **Variable** | **Cohort** | **Inhalant atopy** | **No inhalant atopy** | **P-value** |
| --- | --- | --- | --- | --- |
| No. patients | 308 | 70 | 208 |  |
| Institution, n(%) |  |  |  | <0.001 |
| Edmonton | 37 (12%) | 9 (12.9%) | 19 (9.1%) |  |
| Toronto | 80 (26%) | 28 (40%) | 40 (19.2%) |  |
| Vancouver | 86 (27.9%) | 24 (34.3%) | 58 (27.9%) |  |
| Winnipeg | 105 (34.1%) | 9 (12.9%) | 91 (43.8%) |  |
| Antibiotics use by age 1 year, n(%) | |  |  | 1 |
|  | 5 (1.6%) | 1 (1.4%) | 3 (1.4%) |  |
| Unknown | 1 (0.3%) |  | 1 (0.5%) |  |
| Mode of Delivery, n(%) |  |  |  | 0.15 |
| C-Section with labour | 44 (14.3%) | 13 (18.6%) | 25 (12%) |  |
| C-Section without labour | 34 (11%) | 11 (15.7%) | 21 (10.1%) |  |
| Vaginal | 226 (73.4%) | 46 (65.7%) | 158 (76%) |  |
| Unknown | 4 (1.3%) |  | 4 (1.9%) |  |
| Having Older Sibling, n(%) | |  |  | 0.78 |
|  | 129 (41.9%) | 32 (45.7%) | 87 (41.8%) |  |
| Unknown | 6 (1.9%) |  | 6 (2.9%) |  |
| Breastfeeding status at age 1 year, n(%) | | |  | 0.16 |
|  | 139 (45.1%) | 26 (37.1%) | 100 (48.1%) |  |
| Unknown | 7 (2.3%) | 3 (4.3%) | 3 (1.4%) |  |
| Season of Birth, n(%) |  |  |  | 0.028 |
| Spring | 95 (30.8%) | 19 (27.1%) | 66 (31.7%) |  |
| Summer | 80 (26%) | 13 (18.6%) | 60 (28.8%) |  |
| Fall | 68 (22.1%) | 24 (34.3%) | 36 (17.3%) |  |
| Winter | 65 (21.1%) | 14 (20%) | 46 (22.1%) |  |
| Area Type (Rural), n(%) |  |  |  | 0.12 |
|  | 26 (8.4%) | 2 (2.9%) | 18 (8.7%) |  |
| Unknown | 13 (4.2%) | 3 (4.3%) | 10 (4.8%) |  |
| Perinatal pet exposure, n(%) | |  |  | 0.013 |
|  | 138 (44.8%) | 21 (30%) | 104 (50%) |  |
| Unknown | 21 (6.8%) | 8 (11.4%) | 10 (4.8%) |  |
| Presence of mould in home, n(%) | |  |  | 0.019 |
|  | 130 (42.2%) | 39 (55.7%) | 82 (39.4%) |  |
| Antifungal use by age 13 months, n(%) | | |  | 0.21 |
|  | 23 (7.5%) | 3 (4.3%) | 20 (9.6%) |  |
| Solid food by age 3 months, n(%) | |  |  | 1 |
|  | 26 (8.4%) | 5 (7.1%) | 17 (8.2%) |  |
| Unknown | 7 (2.3%) |  | 7 (3.4%) |  |
