# Supplementary material for: Composition and Associations of the Infant Gut Fungal Microbiota with Environmental Factors and Childhood Allergic Outcomes
Source: mBio. 2021 Jun 1;12(3):e03396-20. doi: 10.1128/mBio.03396-20 (PMC8263004; doi:10.1128/mBio.03396-20)
Supplement: TABLE S3 [file mbio.03396-20-st003.docx]

Table S3. Univariate analysis of differences between gut microbiota fungal community composition at three months and one year of age according to health outcomes assessed at age five years based on principal coordinate analysis using unweighted Unifrac and determined by permutational analysis of variance. R^2^ and p-values are adjusted for sequencing batch (batch R^2^=0.0104, p=0.0030 in entire dataset). Single stars indicate p<0.1, double stars indicate p<0.05.

| **Health outcome** | **Three months** | | **One year** | |
| --- | --- | --- | --- | --- |
|  | **R^2^** | **p-value** | **R^2^** | **p-value** |
| Inhalant atopy | 0.0299 | 0.16 | 0.00504 | 0.31 |
| Atopy | 0.0159 | 0.61 | 0.00472 | 0.37 |
| Asthma | 0.0119 | 0.80 | 0.00390 | 0.59 |
